# Supplementary material for: Experimental Study of Body-Fin Interaction and Vortex Dynamics Generated by a Two Degree-Of-Freedom Fish Model
Source: Biomimetics (Basel). 2019 Oct 8;4(4):67. doi: 10.3390/biomimetics4040067 (PMC6963735; doi:10.3390/biomimetics4040067)
Supplement: Supplementary file 1 [file biomimetics-04-00067-s001.zip › Brooks_Green_Supplemental_Materials/Supplemental_Materials.pdf]

Supplementary Materials: Experimental Study of Body-Fin Interaction and Vortex Dynamics Generated by a Two Degree-of-Freedom Fish Model

S.A. Brooks [1,*] and M.A. Green [1]

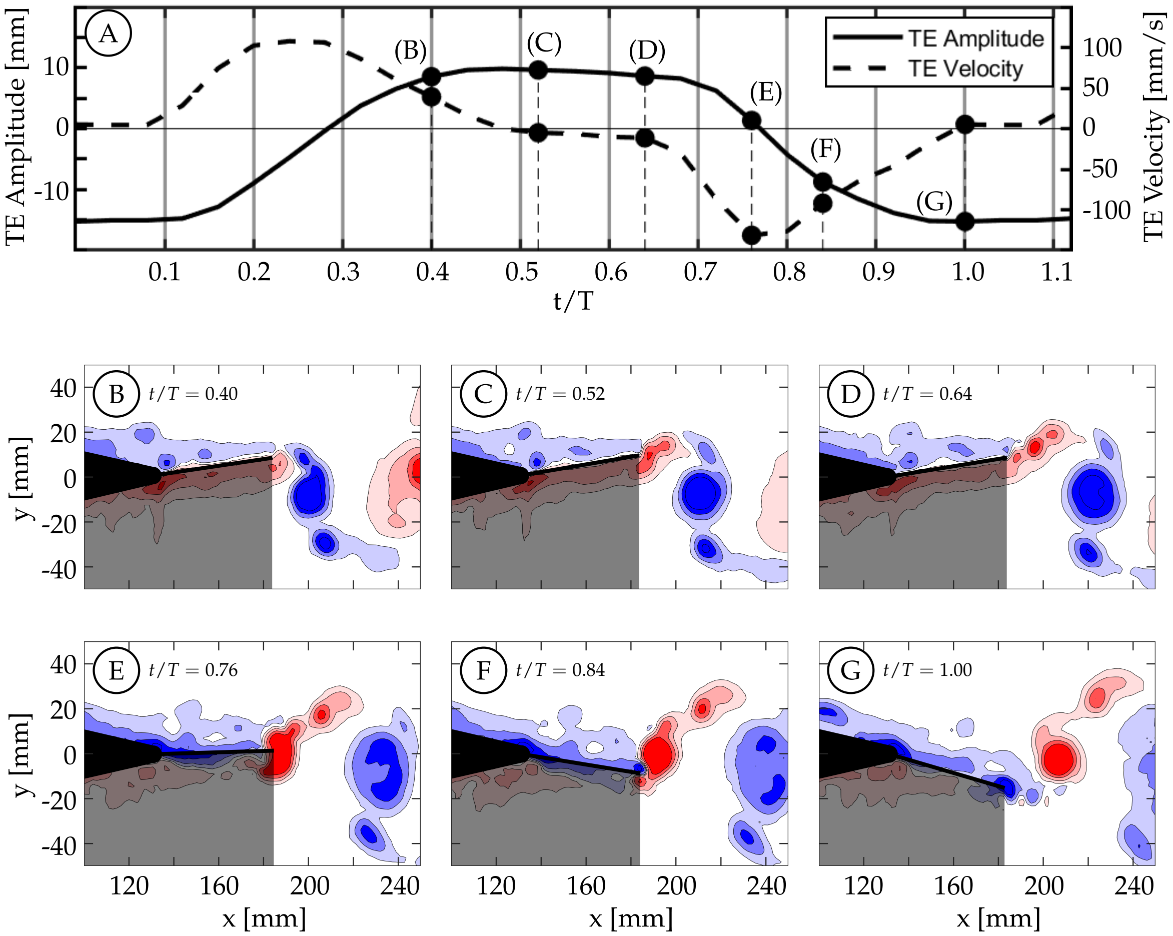

Figure S1. (Case 1: SG1, KG1) Spanwise vorticity ($\omega_z = \pm[1, 4, 9, 16]s^{-1}$) contours are shown here for case 1 which has $\theta_{T,o} = 0.24°$, $\theta_{C,o} = 12.90°$, and $St = 0.308$. Positive spanwise vorticity is shown in red and negative in blue: (**A**) The trailing edge motion profile where the solid curve represents the trailing edge amplitude and the dashed curve represents the trailing edge velocity. (**B**) $t/T = 0.40$. (**C**) $t/T = 0.52$. (**D**) $t/T = 0.64$. (**E**) $t/T = 0.76$. (**F**) $t/T = 0.84$. (**G**) $t/T = 1.00$.

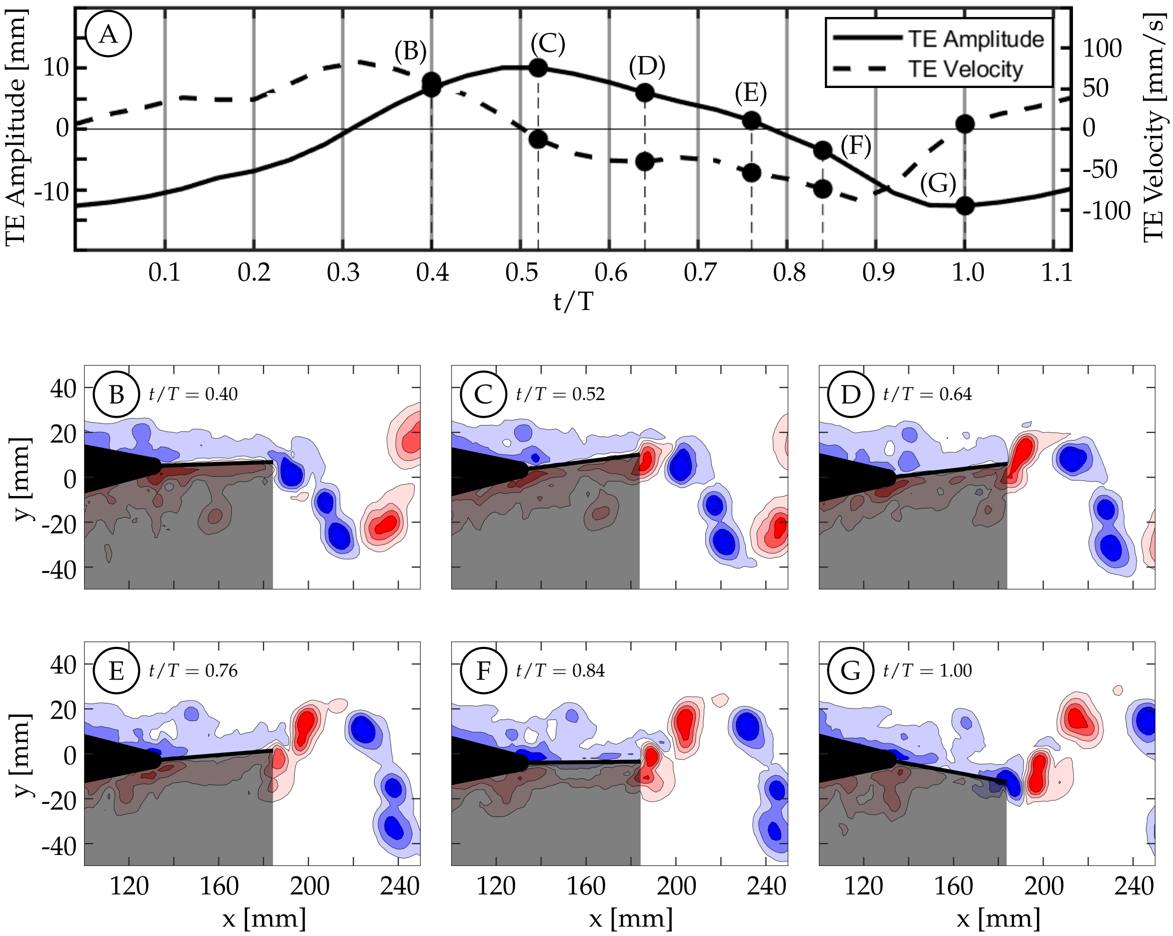

Figure S2. (Case 2: SG1, KG2) Spanwise vorticity ($\omega_z = \pm[1, 4, 9, 16]s^{-1}$) contours are shown here for case 2 which has $\theta_{T,o} = 2.01°$, $\theta_{C,o} = 9.03°$, and $St = 0.279$. Positive spanwise vorticity is shown in red and negative in blue: (**A**) The trailing edge motion profile where the solid curve represents the trailing edge amplitude and the dashed curve represents the trailing edge velocity. (**B**) $t/T = 0.40$. (**C**) $t/T = 0.52$. (**D**) $t/T = 0.64$. (**E**) $t/T = 0.76$. (**F**) $t/T = 0.84$. (**G**) $t/T = 1.00$.

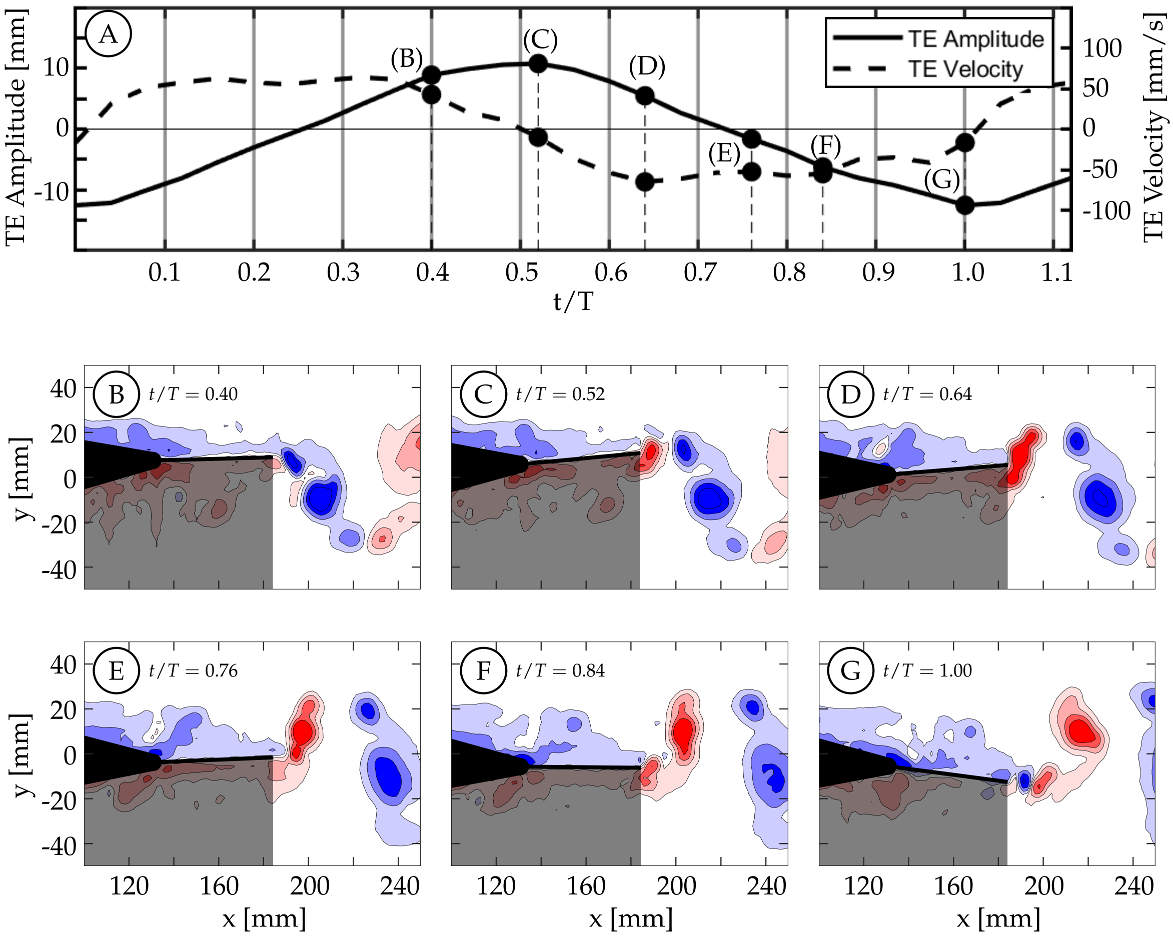

Figure S3. (Case 3: SG1, KG3) Spanwise vorticity ($\omega_z = \pm[1, 4, 9, 16]s^{-1}$) contours are shown here for case 3 which has $\theta_{T,o} = 3.08°$, $\theta_{C,o} = 5.48°$, and $St = 0.286$. Positive spanwise vorticity is shown in red and negative in blue: (**A**) The trailing edge motion profile where the solid curve represents the trailing edge amplitude and the dashed curve represents the trailing edge velocity. (**B**) $t/T = 0.40$. (**C**) $t/T = 0.52$. (**D**) $t/T = 0.64$. (**E**) $t/T = 0.76$. (**F**) $t/T = 0.84$. (**G**) $t/T = 1.00$.

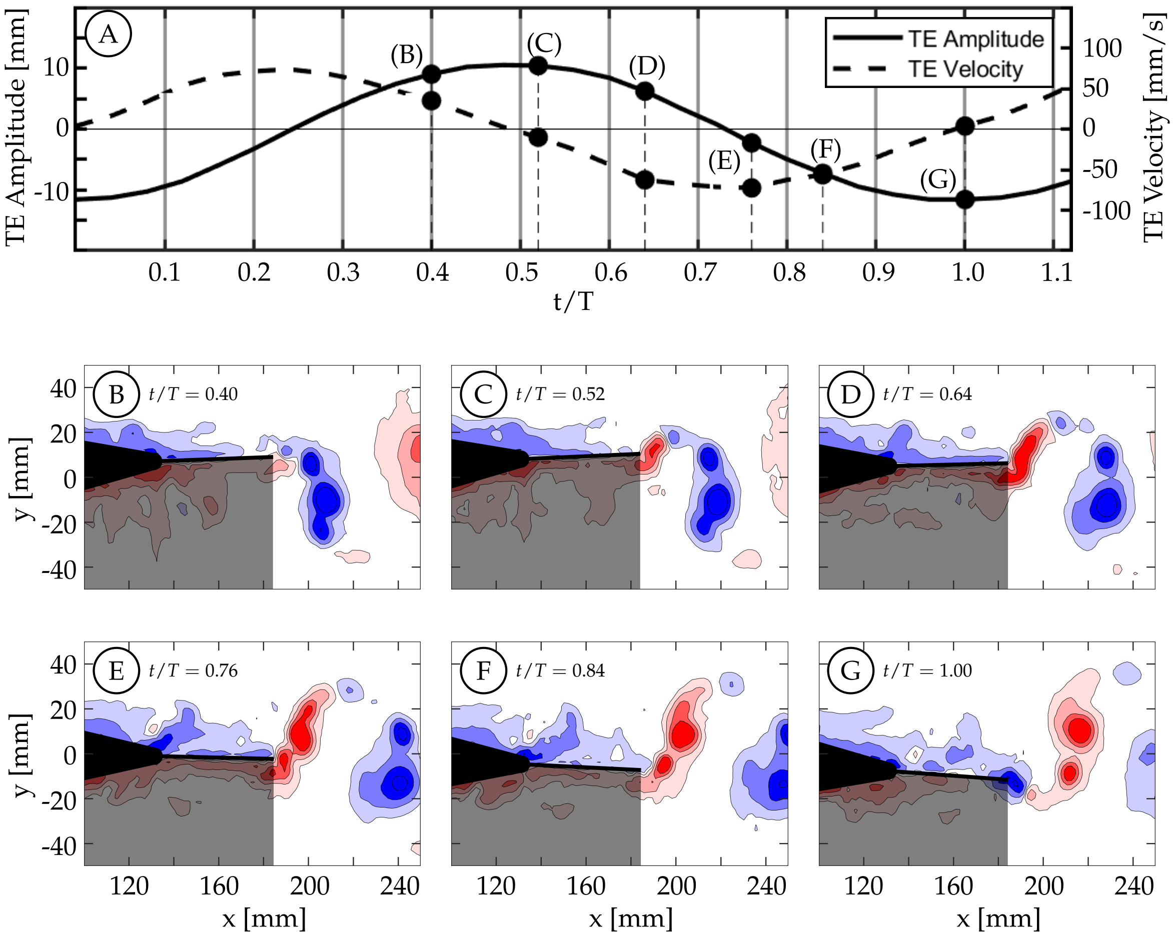

Figure S4. (Case 4: SG1, KG4) Spanwise vorticity ($\omega_z = \pm[1, 4, 9, 16]s^{-1}$) contours are shown here for case 4 which has $\theta_{T,o} = 3.44°$, $\theta_{C,o} = 0.18°$, and $St = 0.271$. Positive spanwise vorticity is shown in red and negative in blue: (**A**) The trailing edge motion profile where the solid curve represents the trailing edge amplitude and the dashed curve represents the trailing edge velocity. (**B**) $t/T = 0.40$. (**C**) $t/T = 0.52$. (**D**) $t/T = 0.64$. (**E**) $t/T = 0.76$. (**F**) $t/T = 0.84$. (**G**) $t/T = 1.00$.

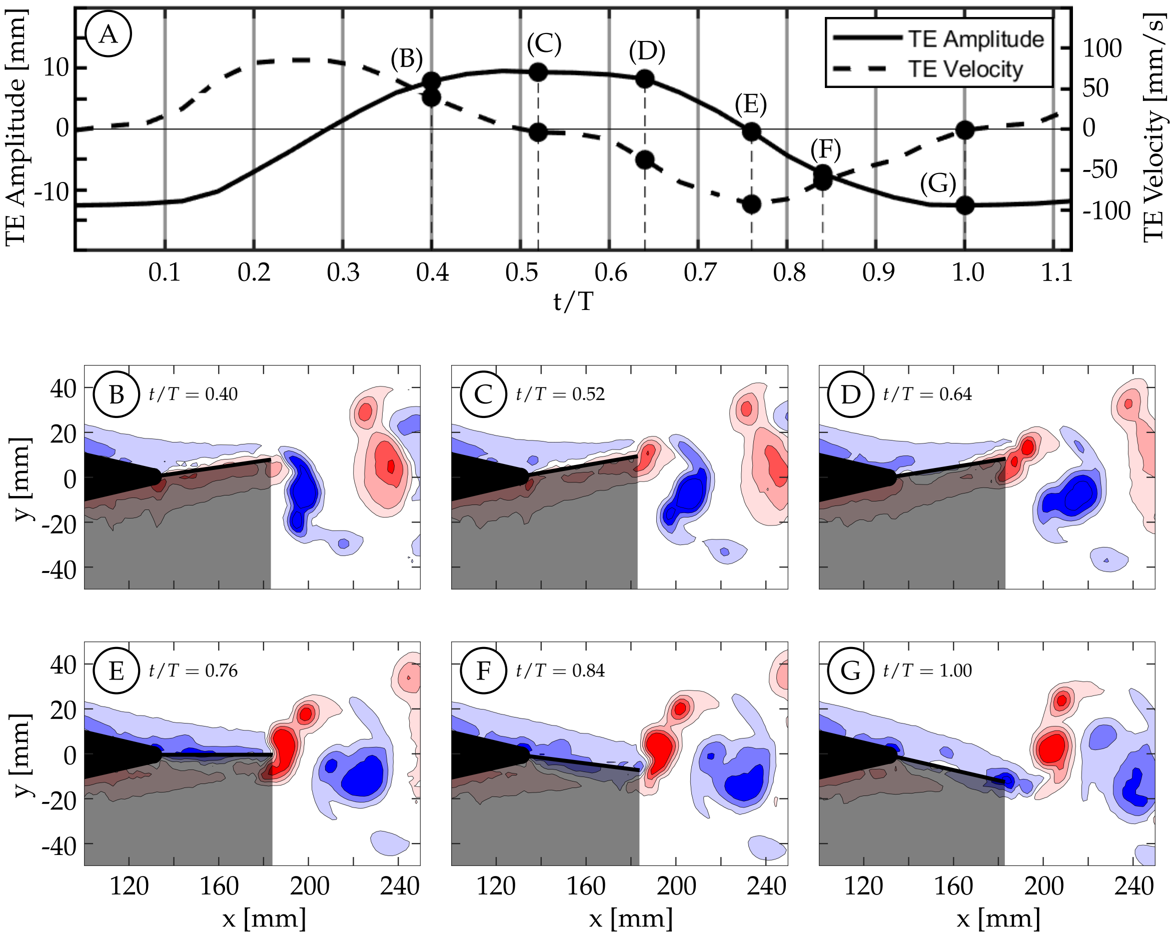

Figure S5. (Case 5: SG2, KG1) Spanwise vorticity ($\omega_z = \pm[1, 4, 9, 16]s^{-1}$) contours are shown here for case 5 which has $\theta_{T,o} = 0.22°$, $\theta_{C,o} = 11.18°$, and $St = 0.371$. Positive spanwise vorticity is shown in red and negative in blue: (**A**) The trailing edge motion profile where the solid curve represents the trailing edge amplitude and the dashed curve represents the trailing edge velocity. (**B**) $t/T = 0.40$. (**C**) $t/T = 0.52$. (**D**) $t/T = 0.64$. (**E**) $t/T = 0.76$. (**F**) $t/T = 0.84$. (**G**) $t/T = 1.00$.

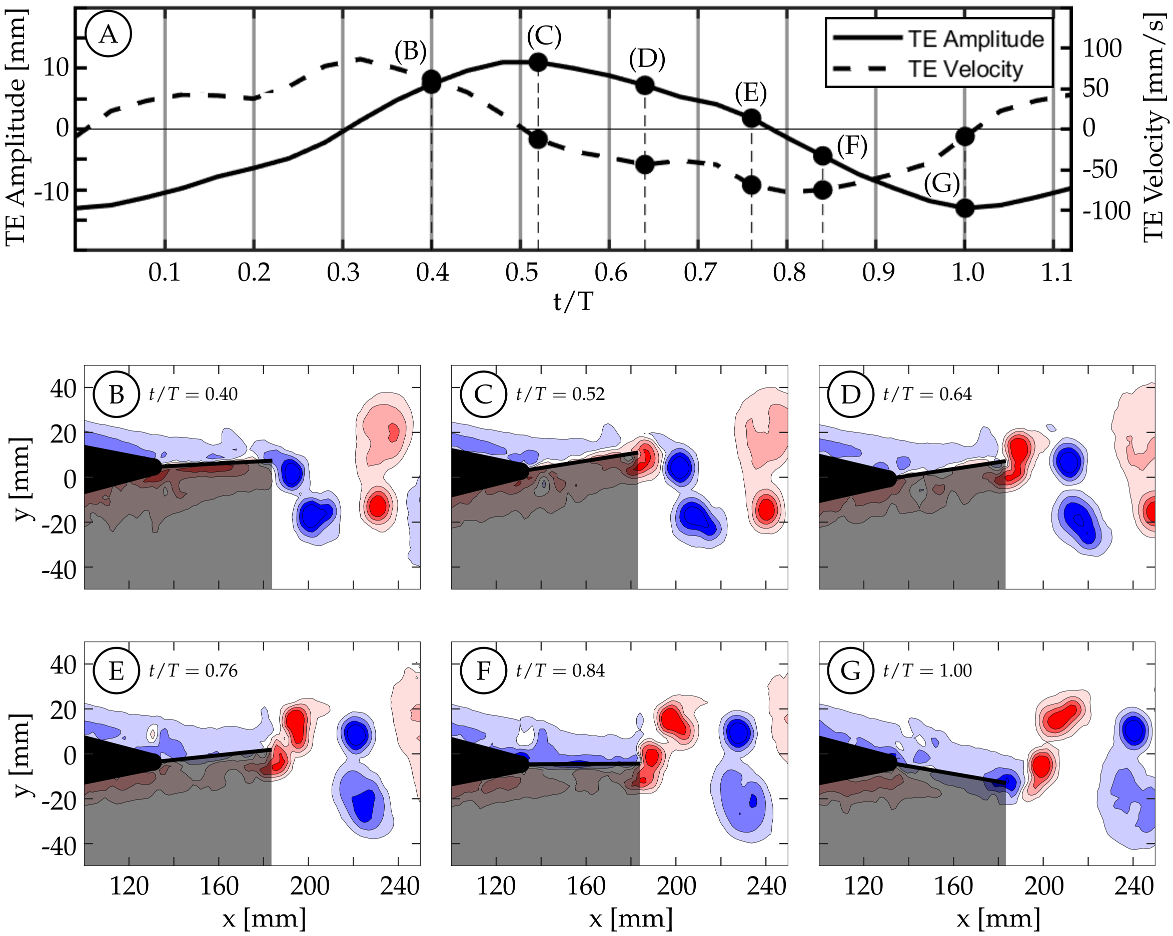

Figure S6. (Case 6: SG2, KG2) Spanwise vorticity ($\omega_z = \pm[1, 4, 9, 16]s^{-1}$) contours are shown here for case 6 which has $\theta_{T,o} = 2.06°$, $\theta_{C,o} = 9.12°$, and $St = 0.403$. Positive spanwise vorticity is shown in red and negative in blue: (**A**) The trailing edge motion profile where the solid curve represents the trailing edge amplitude and the dashed curve represents the trailing edge velocity. (**B**) $t/T = 0.40$. (**C**) $t/T = 0.52$. (**D**) $t/T = 0.64$. (**E**) $t/T = 0.76$. (**F**) $t/T = 0.84$. (**G**) $t/T = 1.00$.

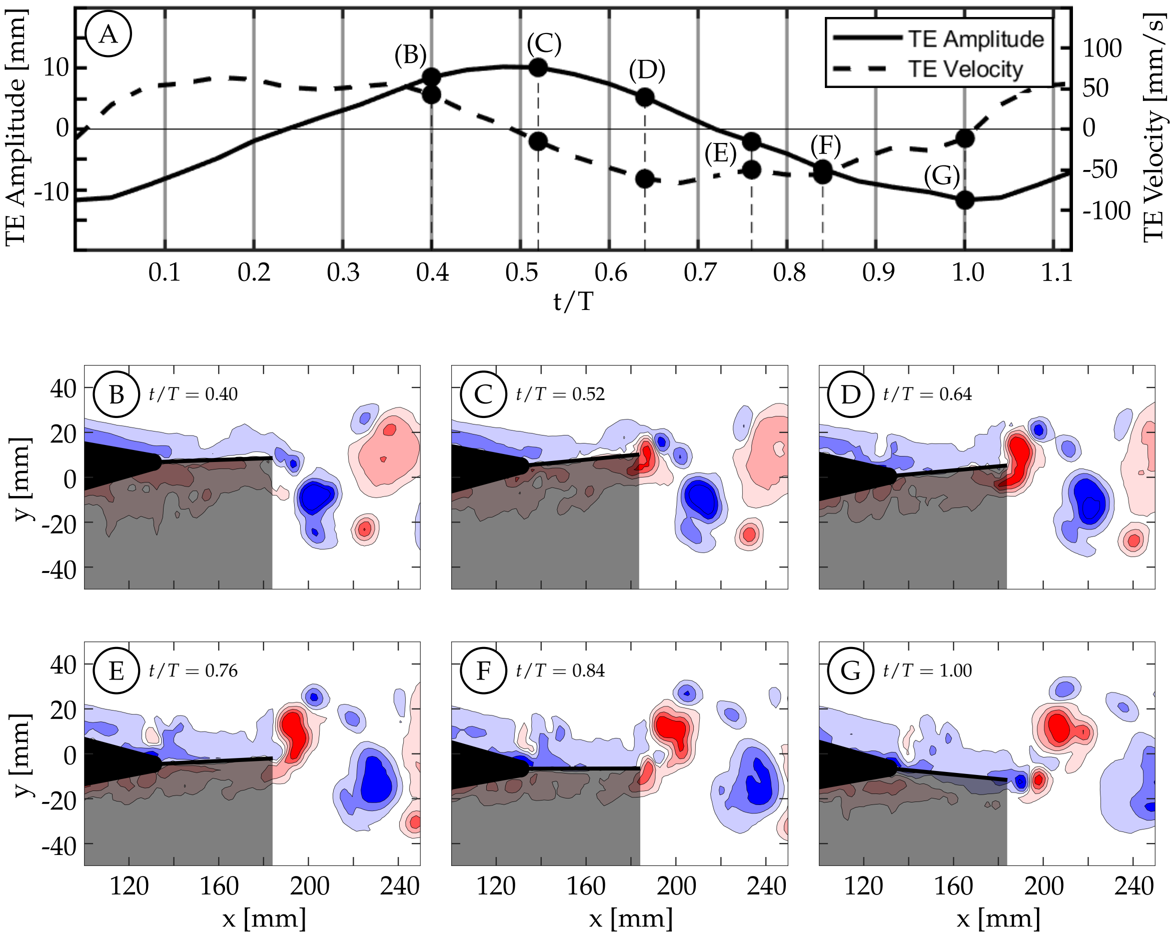

Figure S7. (Case 7: SG2, KG3) Spanwise vorticity ($\omega_z = \pm[1, 4, 9, 16]s^{-1}$) contours are shown here for case 7 which has $\theta_{T,o} = 3.04°$, $\theta_{C,o} = 4.91°$, and $St = 0.368$. Positive spanwise vorticity is shown in red and negative in blue: (**A**) The trailing edge motion profile where the solid curve represents the trailing edge amplitude and the dashed curve represents the trailing edge velocity. (**B**) $t/T = 0.40$. (**C**) $t/T = 0.52$. (**D**) $t/T = 0.64$. (**E**) $t/T = 0.76$. (**F**) $t/T = 0.84$. (**G**) $t/T = 1.00$.

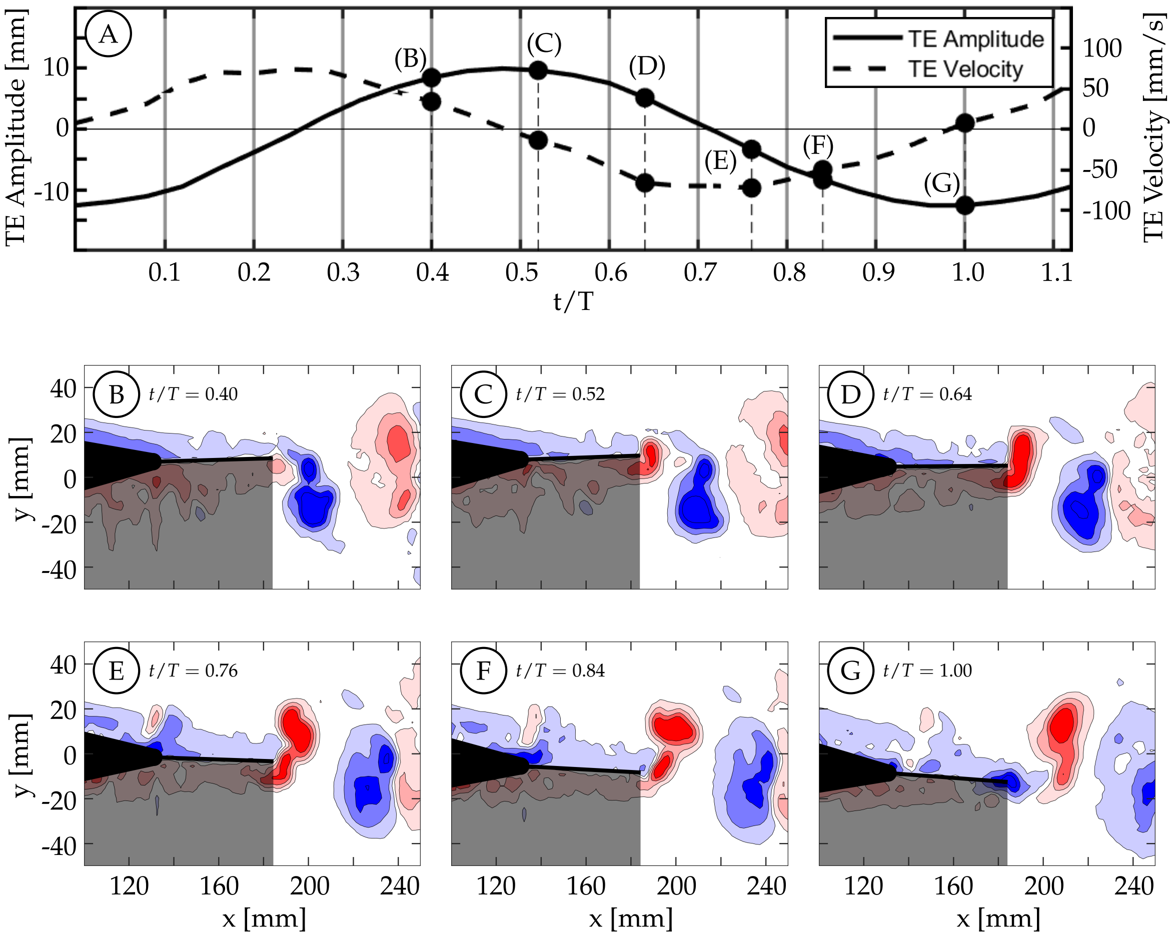

Figure S8. (Case 8: SG2, KG4) Spanwise vorticity ($\omega_z = \pm[1, 4, 9, 16]s^{-1}$) contours are shown here for case 8 which has $\theta_{T,o} = 3.63°$, $\theta_{C,o} = 0.68°$, and $St = 0.378$. Positive spanwise vorticity is shown in red and negative in blue: (**A**) The trailing edge motion profile where the solid curve represents the trailing edge amplitude and the dashed curve represents the trailing edge velocity. (**B**) $t/T = 0.40$. (**C**) $t/T = 0.52$. (**D**) $t/T = 0.64$. (**E**) $t/T = 0.76$. (**F**) $t/T = 0.84$. (**G**) $t/T = 1.00$.

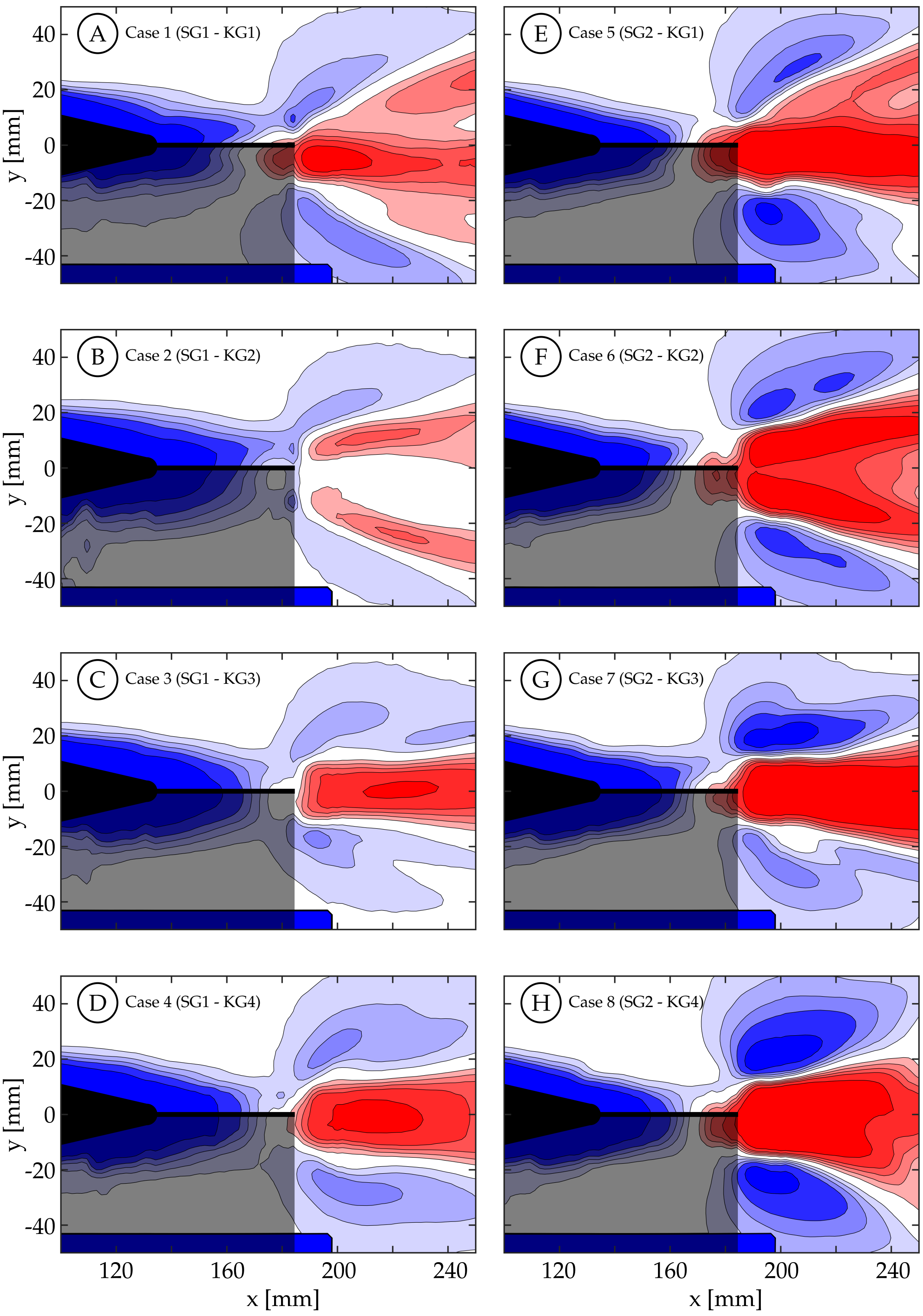

Figure S9. Time averaged x-direction velocity ($U/U_\infty - 1 = \pm[0.05, 0.10, 0.15, 0.20, 0.30]$) contours are shown here for cases 1 through 8 where excess velocity is red and velocity deficit is blue: (**A**) Case 1 - SG1 and KG1. (**B**) Case 2 - SG1 and KG2. (**C**) Case 3 - SG1 and KG3. (**D**) Case 4 - SG1 and KG4. (**E**) Case 5 - SG2 and KG1. (**F**) Case 6 - SG2 and KG2. (**G**) Case 7 - SG2 and KG3. (**H**) Case 8 - SG2 and KG4.
