# Supplementary figures and images for: Experimental Study of Body-Fin Interaction and Vortex Dynamics Generated by a Two Degree-Of-Freedom Fish Model
Source: Biomimetics (Basel). 2019 Oct 8;4(4):67. doi: 10.3390/biomimetics4040067 (PMC6963735; doi:10.3390/biomimetics4040067)

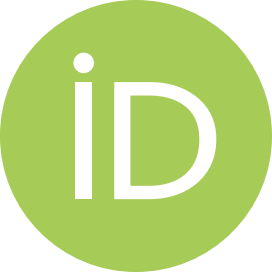

Supplement: Supplementary file 1 [file biomimetics-04-00067-s001.zip › Brooks_Green_Supplemental_Materials/Definitions/logo-orcid-eps-converted-to.pdf]

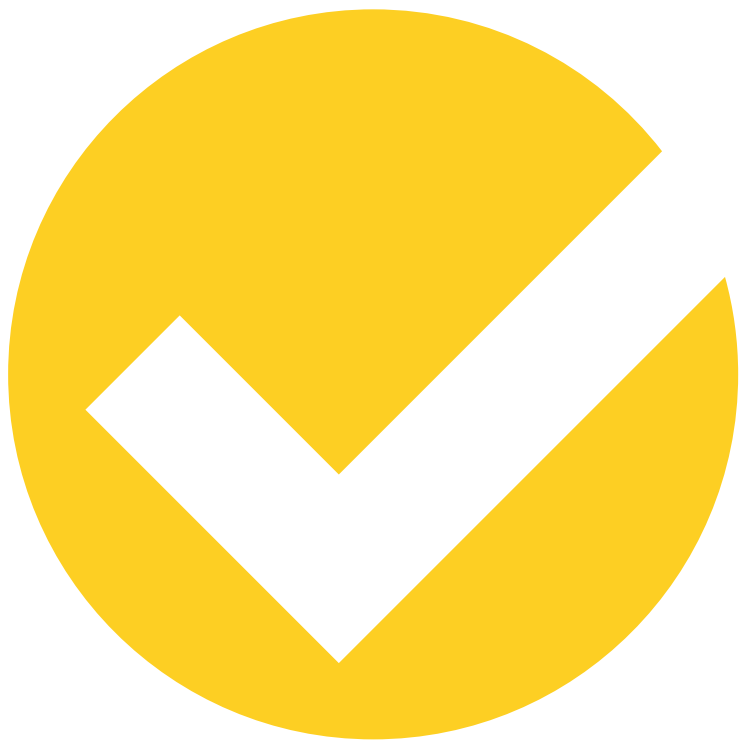

check for  
updates

Supplement: Supplementary file 1 [file biomimetics-04-00067-s001.zip › Brooks_Green_Supplemental_Materials/Definitions/logo-updates.pdf]

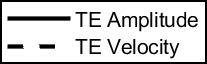

Supplement: Supplementary file 1 [file biomimetics-04-00067-s001.zip › Brooks_Green_Supplemental_Materials/Figures/Legend.png]

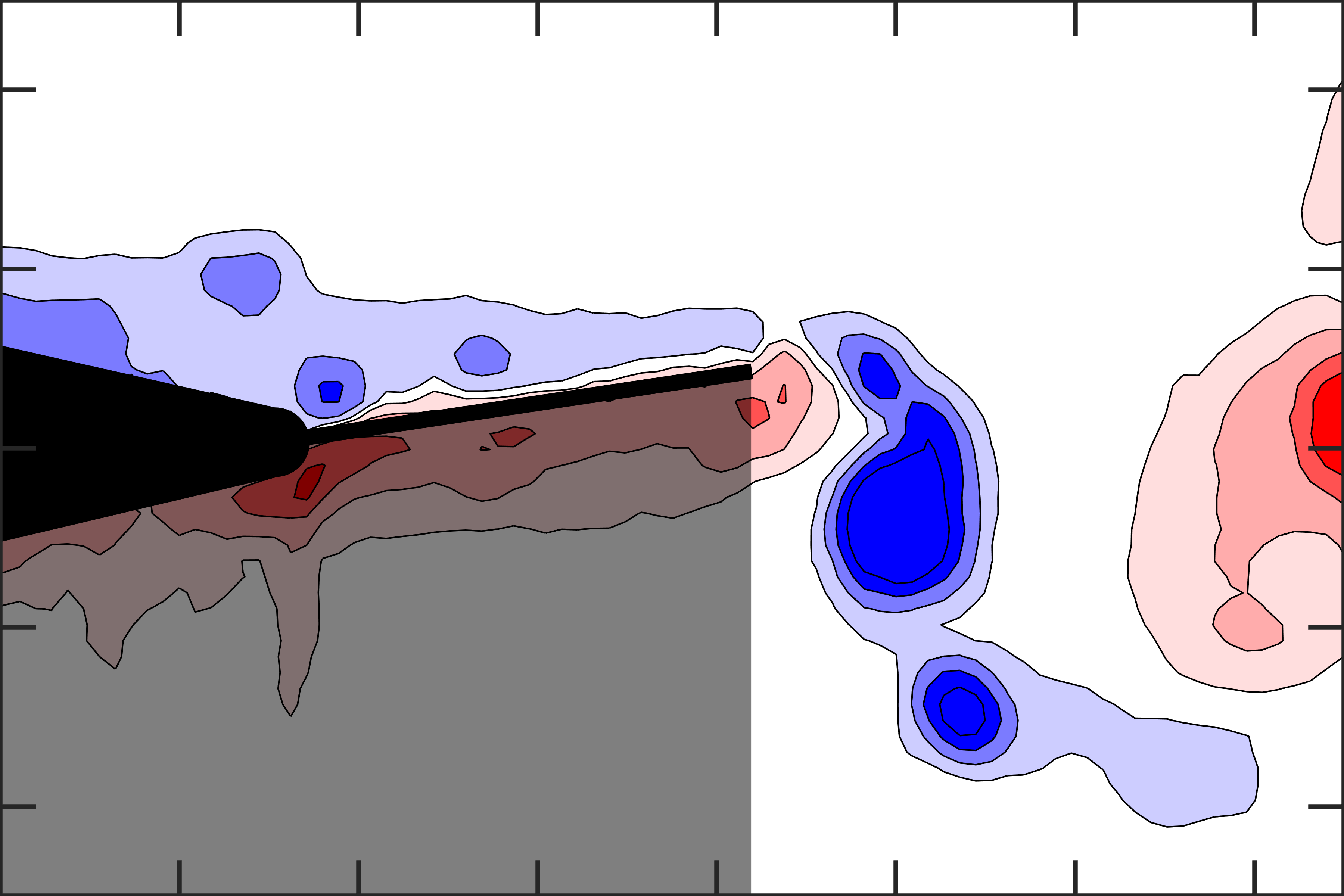

Supplement: Supplementary file 1 [file biomimetics-04-00067-s001.zip › Brooks_Green_Supplemental_Materials/Figures/TEVel_St0p27_T00p00_C15p00_p00mm_pActual11_pRaw10.png]

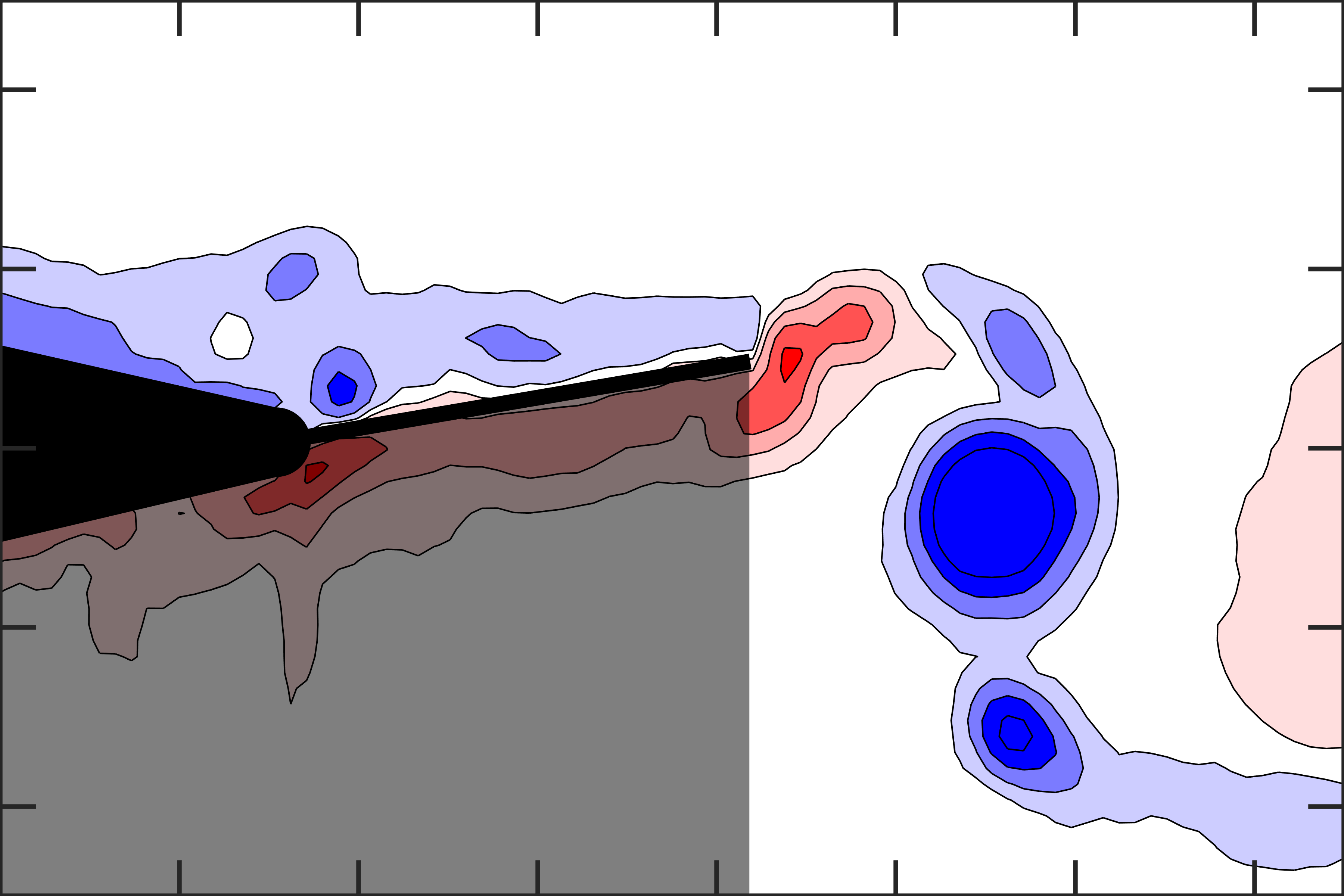

Supplement: Supplementary file 1 [file biomimetics-04-00067-s001.zip › Brooks_Green_Supplemental_Materials/Figures/TEVel_St0p27_T00p00_C15p00_p00mm_pActual14_pRaw13.png]

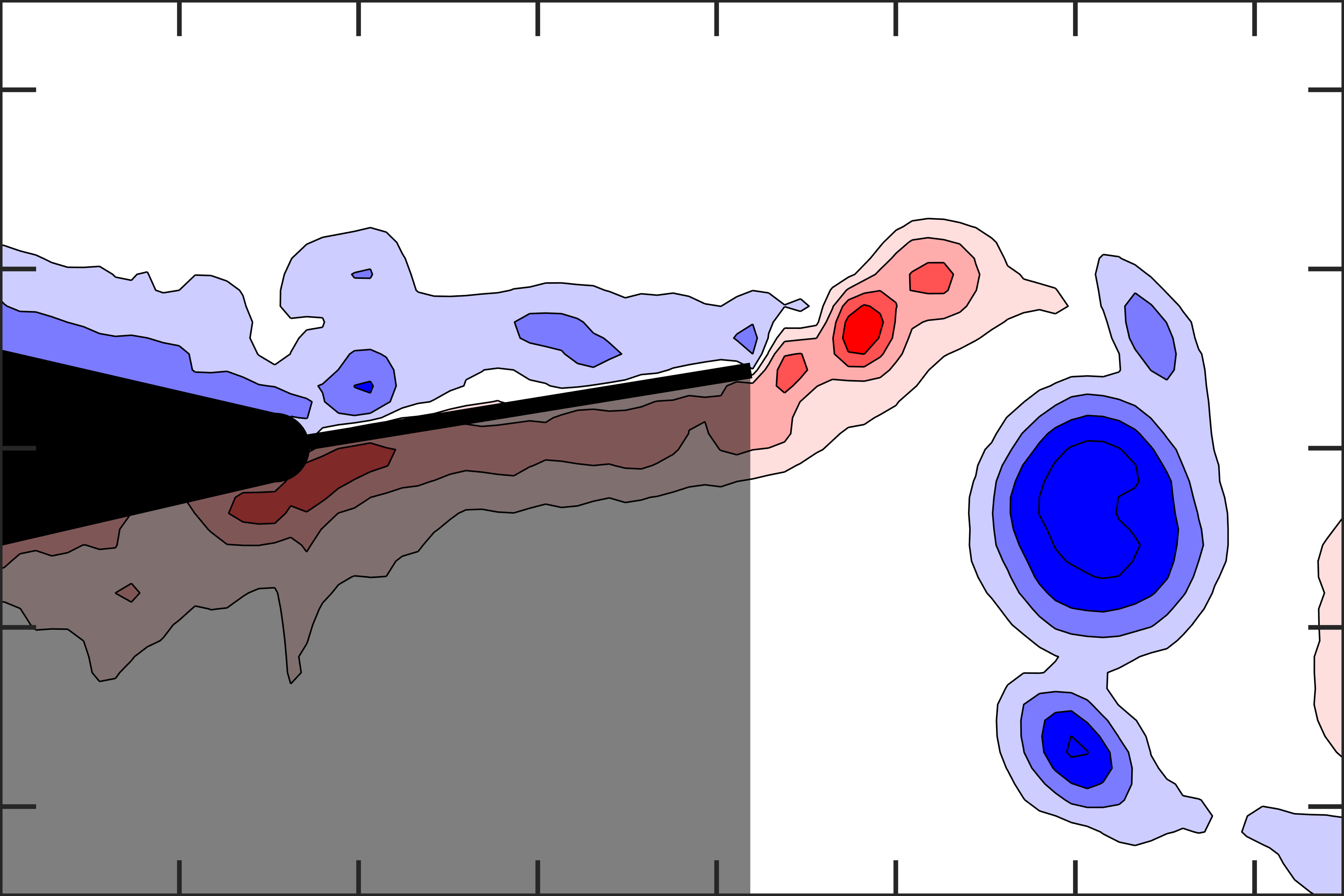

Supplement: Supplementary file 1 [file biomimetics-04-00067-s001.zip › Brooks_Green_Supplemental_Materials/Figures/TEVel_St0p27_T00p00_C15p00_p00mm_pActual17_pRaw16.png]

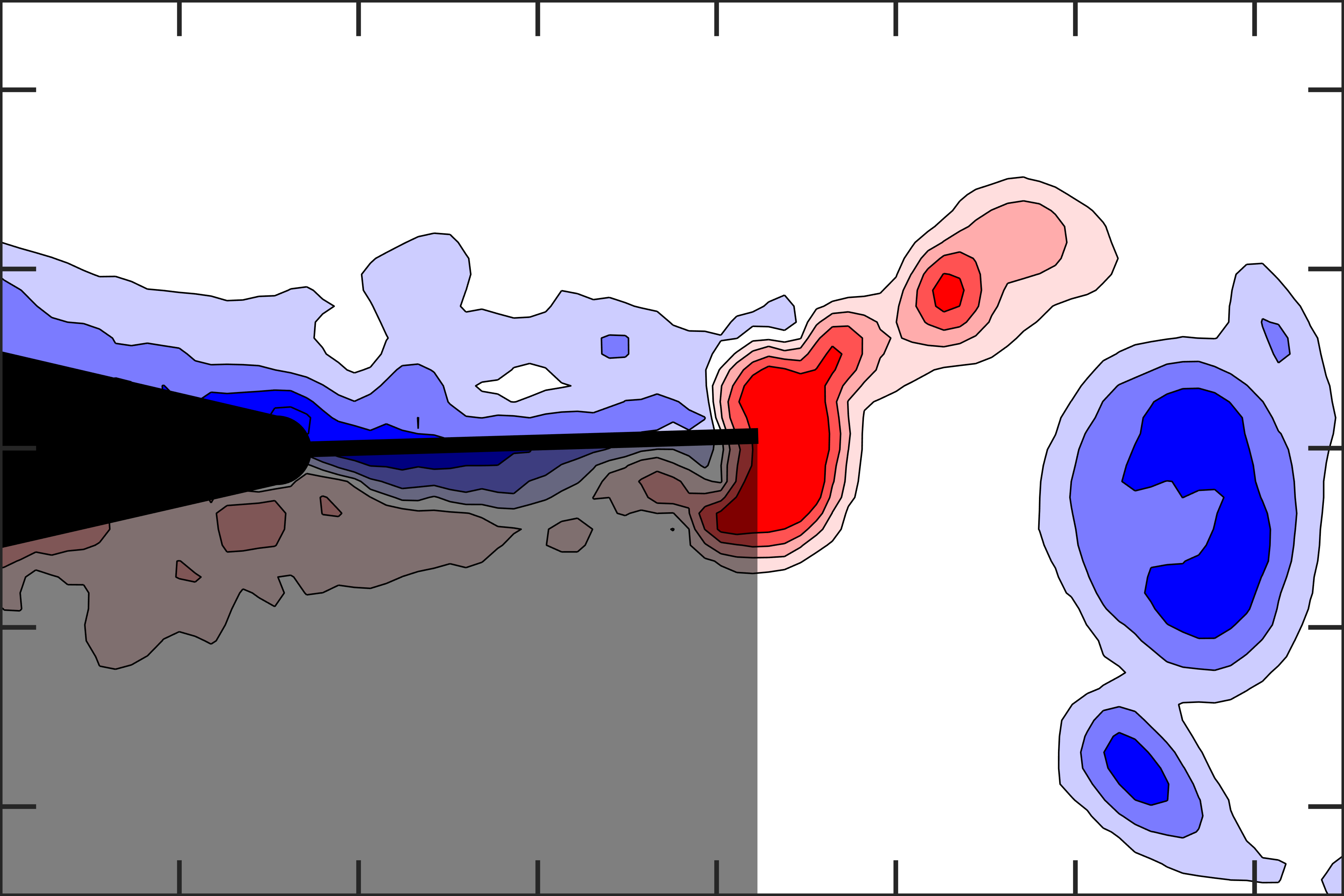

Supplement: Supplementary file 1 [file biomimetics-04-00067-s001.zip › Brooks_Green_Supplemental_Materials/Figures/TEVel_St0p27_T00p00_C15p00_p00mm_pActual20_pRaw19.png]

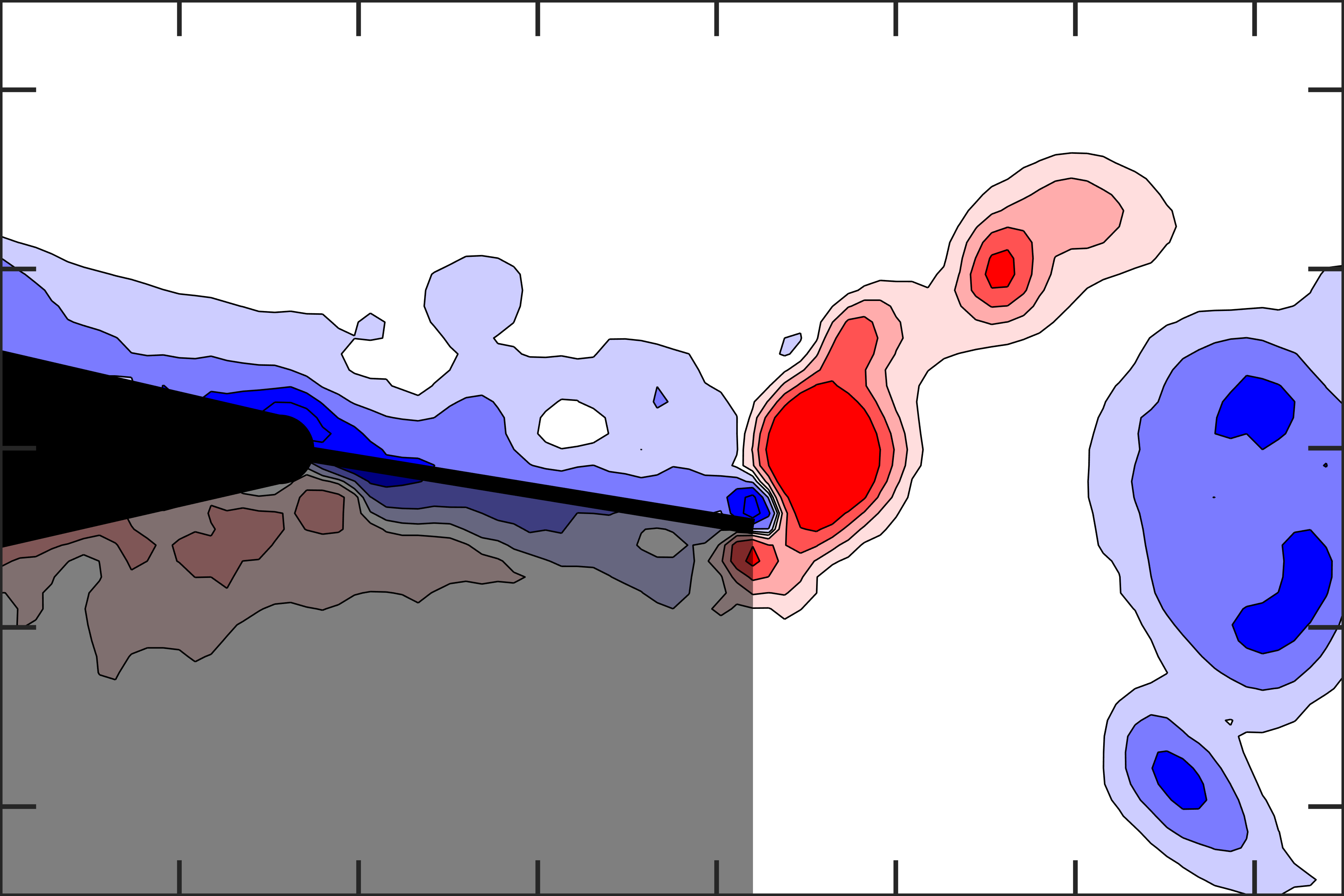

Supplement: Supplementary file 1 [file biomimetics-04-00067-s001.zip › Brooks_Green_Supplemental_Materials/Figures/TEVel_St0p27_T00p00_C15p00_p00mm_pActual22_pRaw21.png]

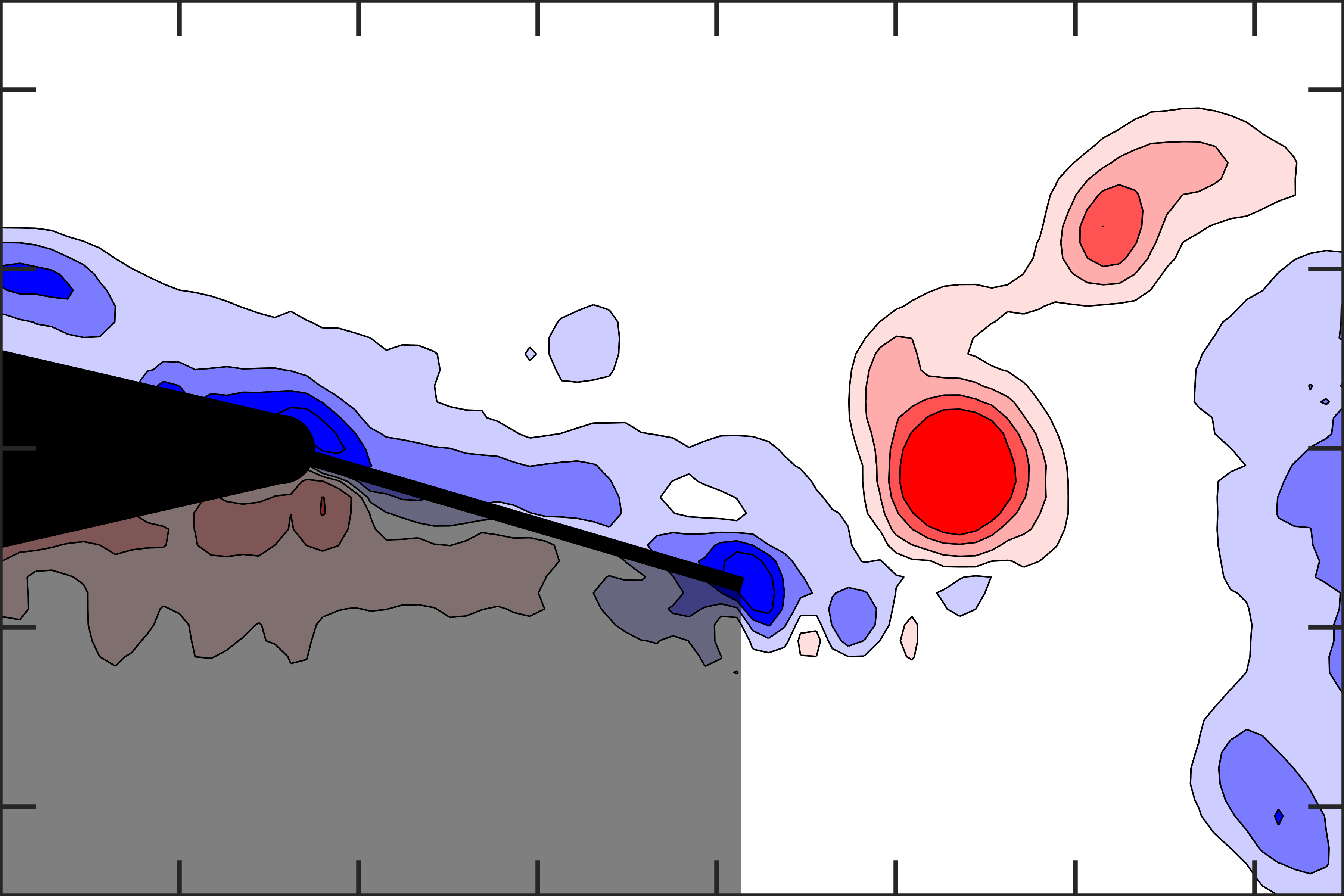

Supplement: Supplementary file 1 [file biomimetics-04-00067-s001.zip › Brooks_Green_Supplemental_Materials/Figures/TEVel_St0p27_T00p00_C15p00_p00mm_pActual26_pRaw25.png]

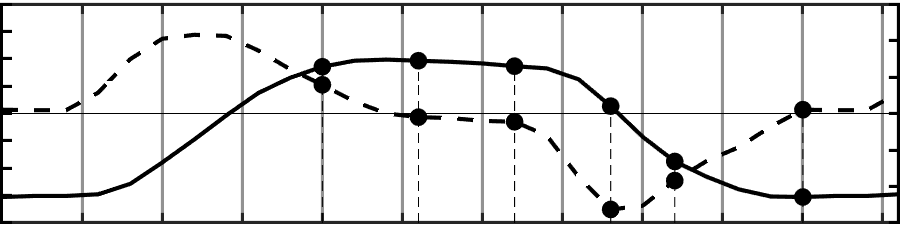

Supplement: Supplementary file 1 [file biomimetics-04-00067-s001.zip › Brooks_Green_Supplemental_Materials/Figures/TEVel_St0p27_T00p00_C15p00_p00mm_Velocity.png]

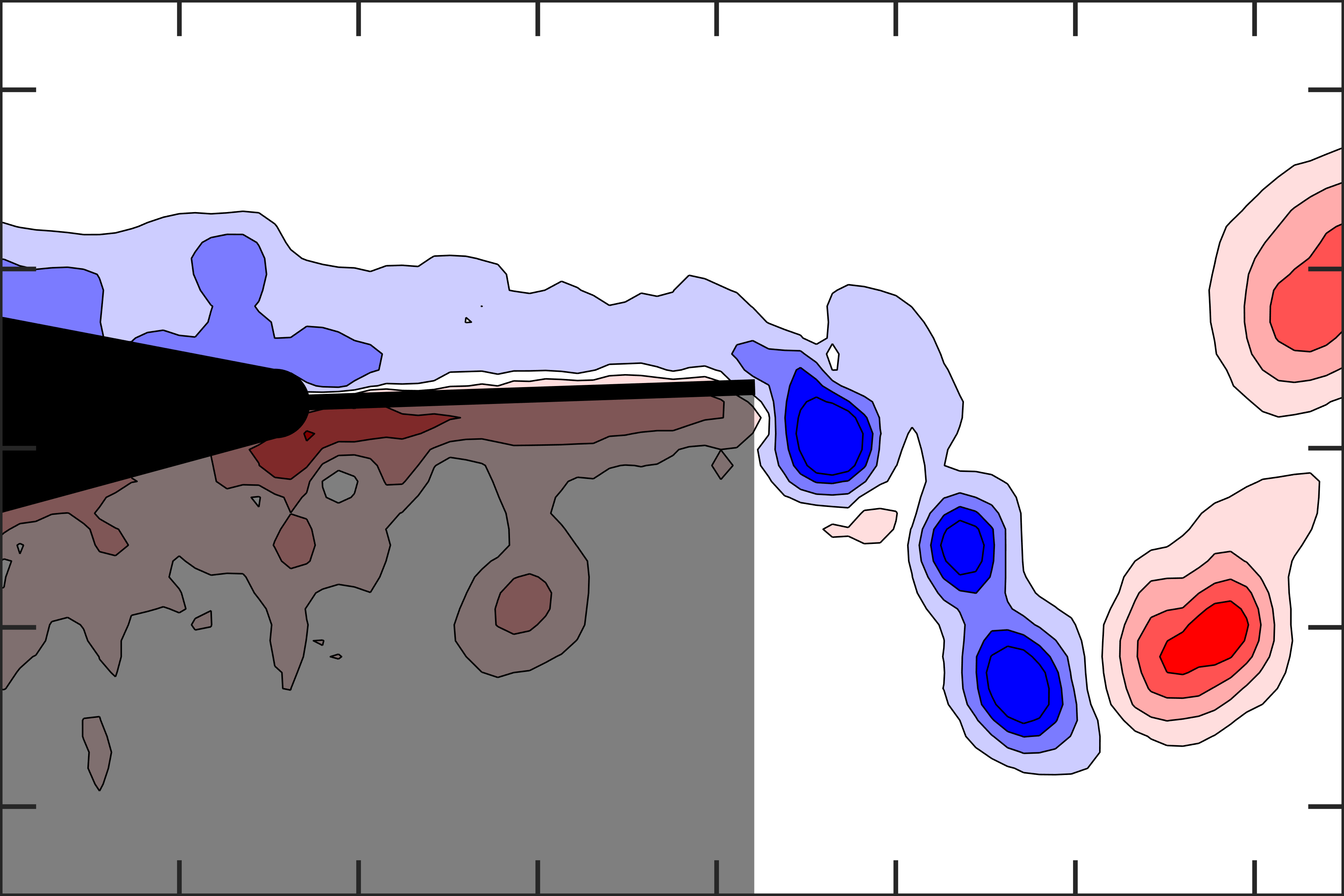

Supplement: Supplementary file 1 [file biomimetics-04-00067-s001.zip › Brooks_Green_Supplemental_Materials/Figures/TEVel_St0p27_T01p99_C10p00_p00mm_pActual11_pRaw08.png]

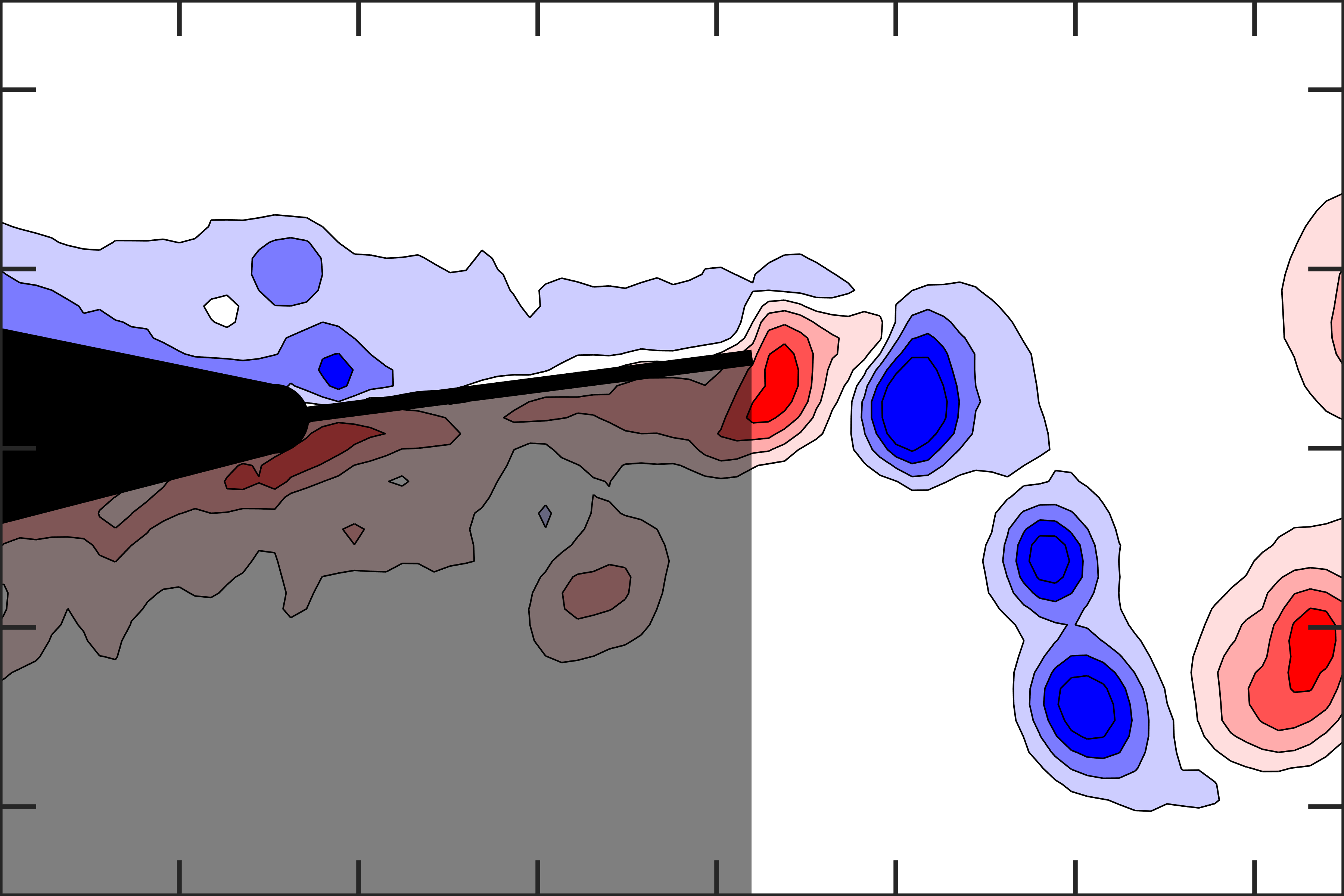

Supplement: Supplementary file 1 [file biomimetics-04-00067-s001.zip › Brooks_Green_Supplemental_Materials/Figures/TEVel_St0p27_T01p99_C10p00_p00mm_pActual14_pRaw11.png]

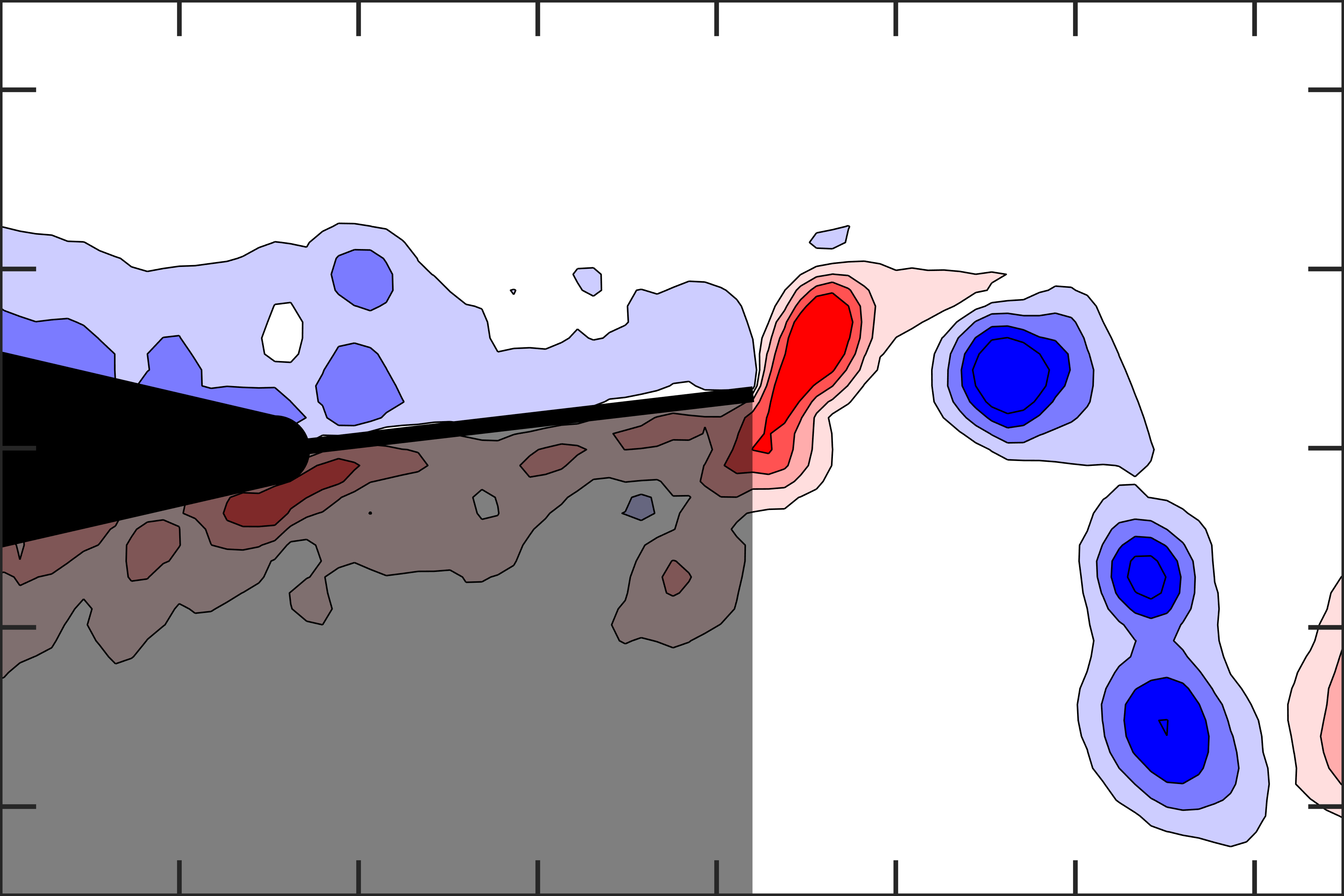

Supplement: Supplementary file 1 [file biomimetics-04-00067-s001.zip › Brooks_Green_Supplemental_Materials/Figures/TEVel_St0p27_T01p99_C10p00_p00mm_pActual17_pRaw14.png]

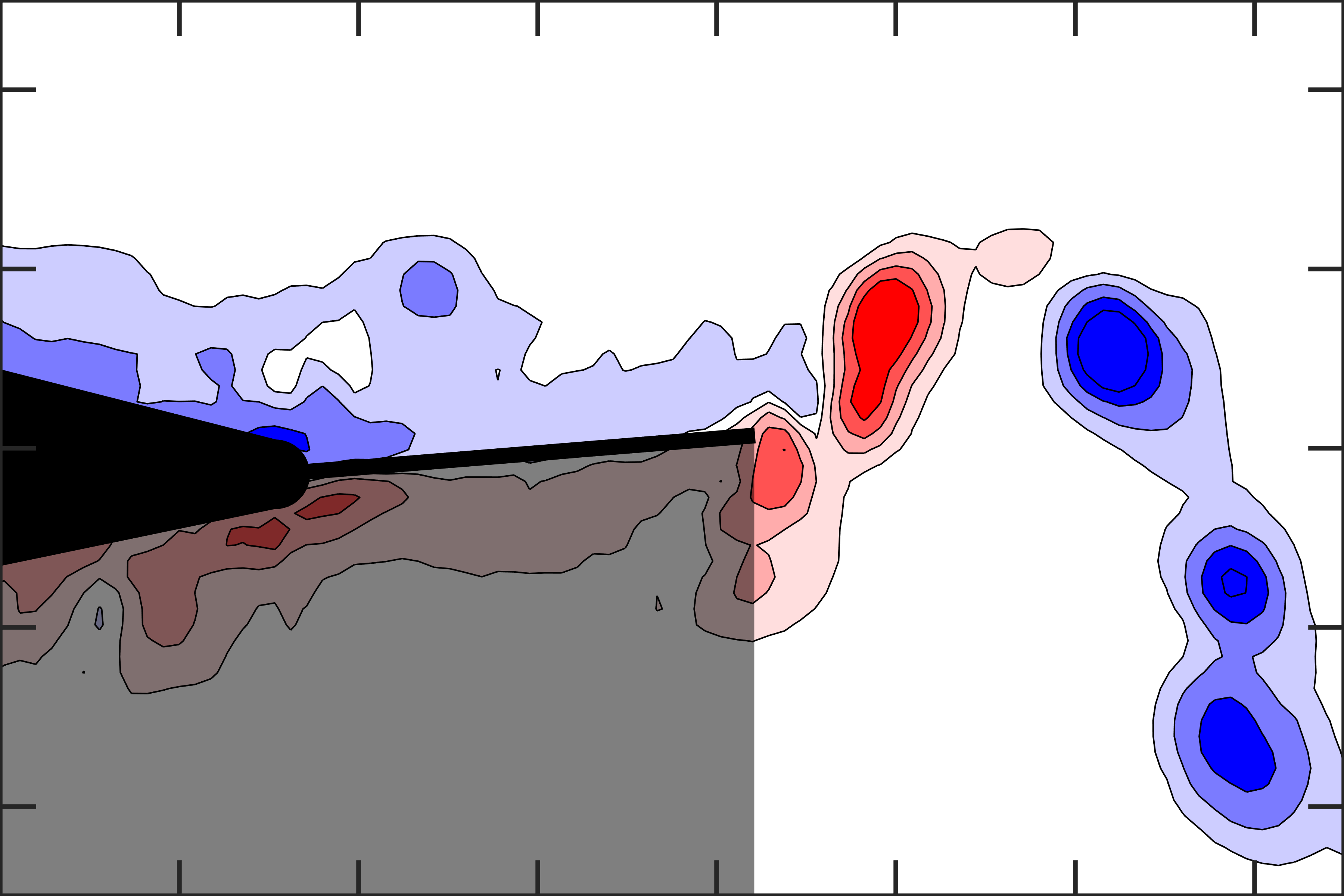

Supplement: Supplementary file 1 [file biomimetics-04-00067-s001.zip › Brooks_Green_Supplemental_Materials/Figures/TEVel_St0p27_T01p99_C10p00_p00mm_pActual20_pRaw17.png]

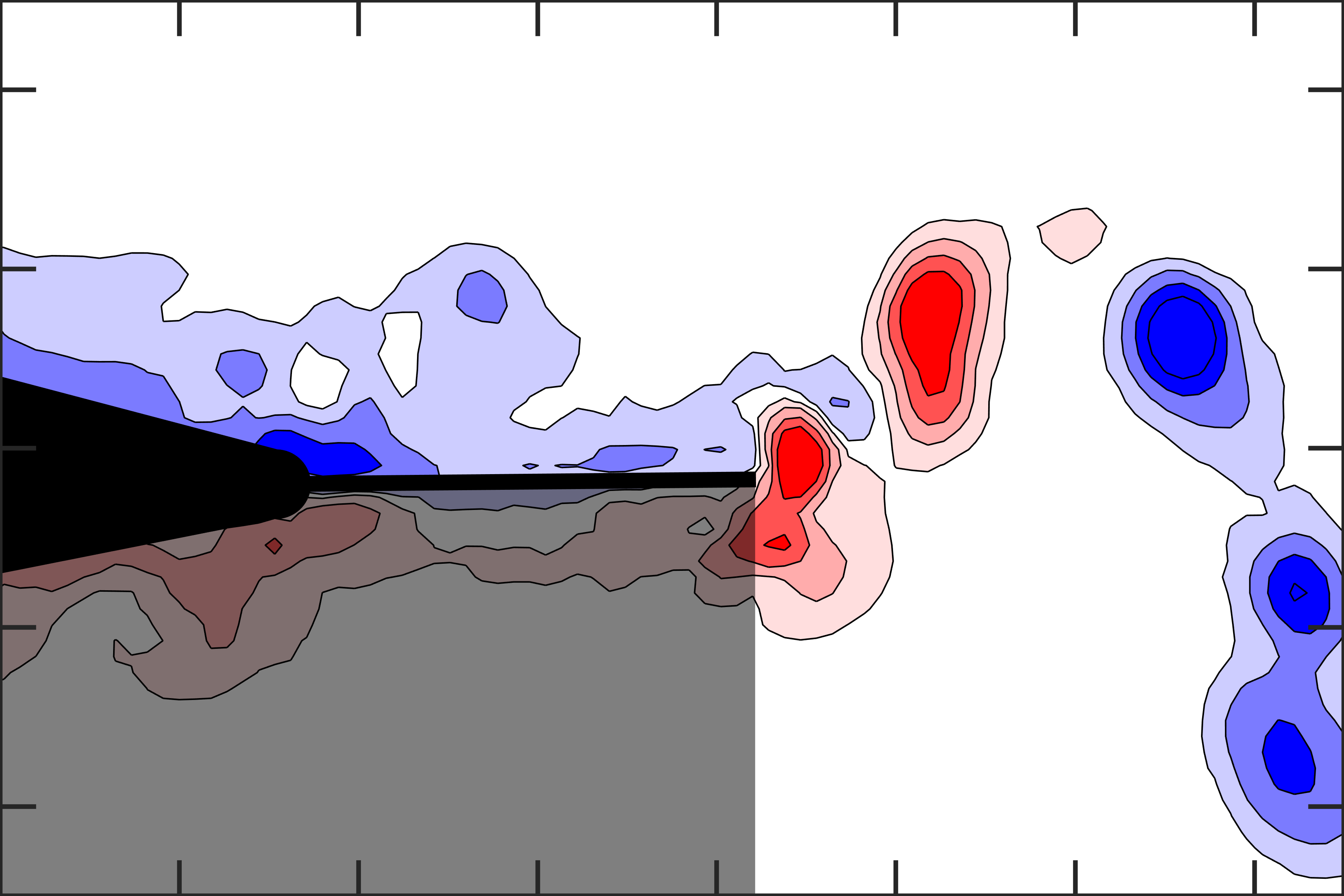

Supplement: Supplementary file 1 [file biomimetics-04-00067-s001.zip › Brooks_Green_Supplemental_Materials/Figures/TEVel_St0p27_T01p99_C10p00_p00mm_pActual22_pRaw19.png]

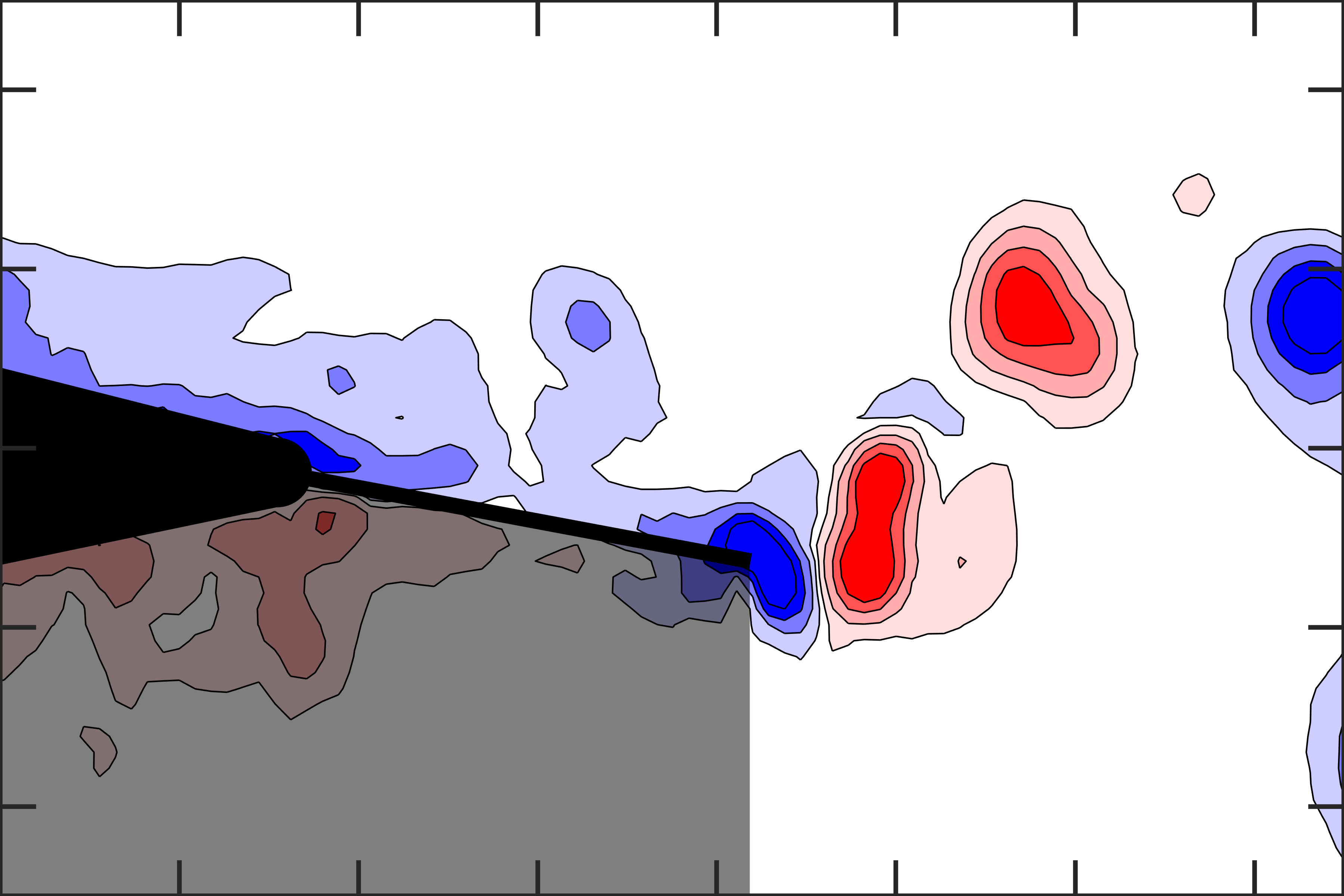

Supplement: Supplementary file 1 [file biomimetics-04-00067-s001.zip › Brooks_Green_Supplemental_Materials/Figures/TEVel_St0p27_T01p99_C10p00_p00mm_pActual26_pRaw23.png]

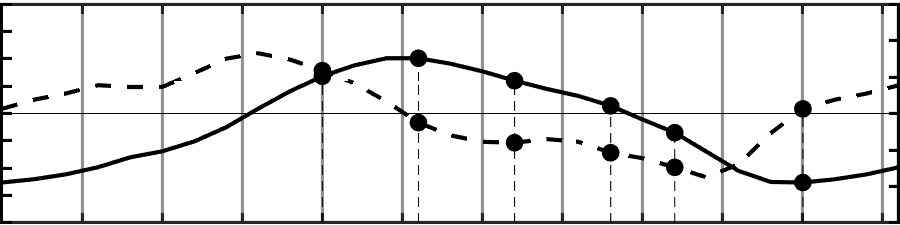

Supplement: Supplementary file 1 [file biomimetics-04-00067-s001.zip › Brooks_Green_Supplemental_Materials/Figures/TEVel_St0p27_T01p99_C10p00_p00mm_Velocity.png]

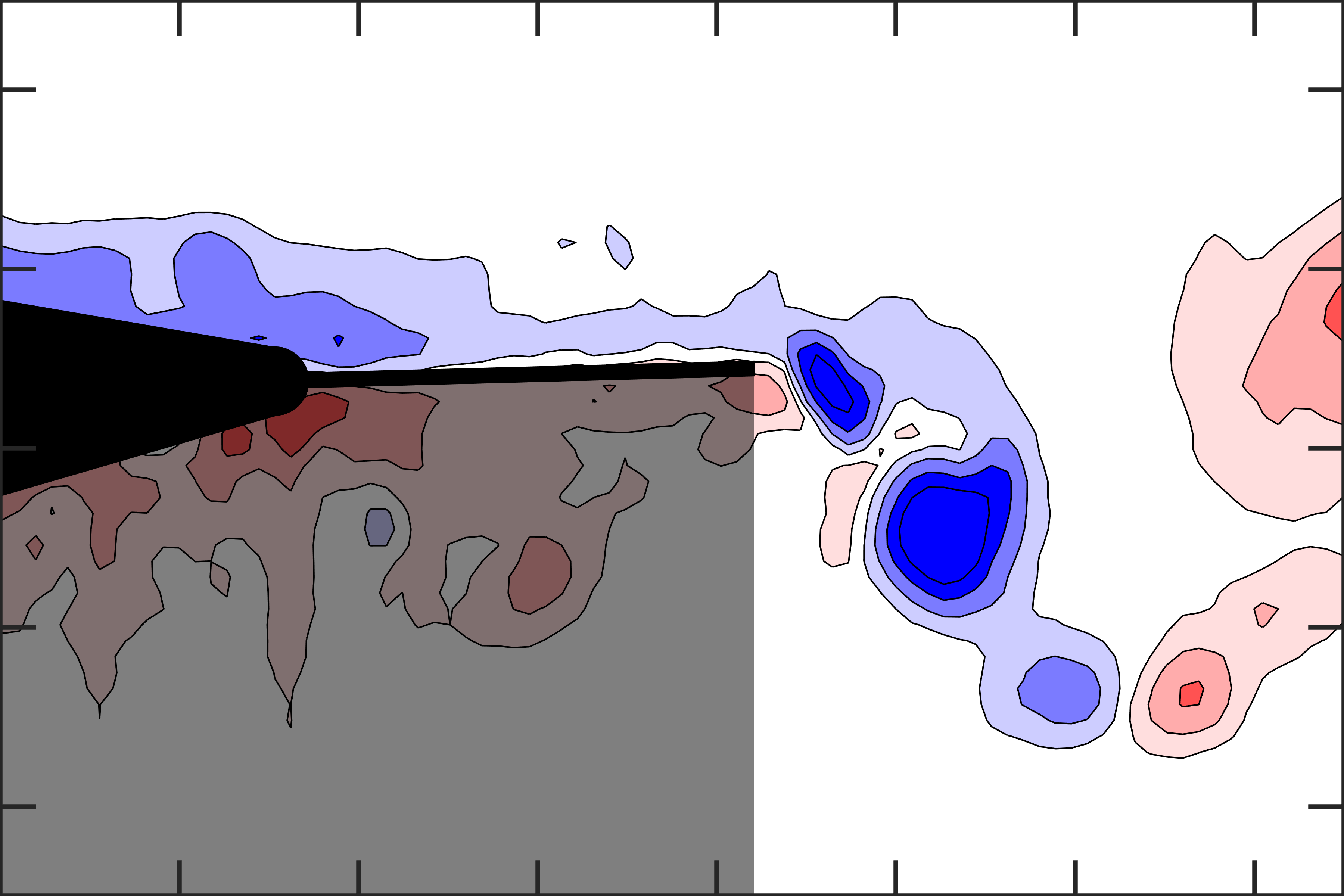

Supplement: Supplementary file 1 [file biomimetics-04-00067-s001.zip › Brooks_Green_Supplemental_Materials/Figures/TEVel_St0p27_T03p03_C05p00_p00mm_pActual11_pRaw07.png]

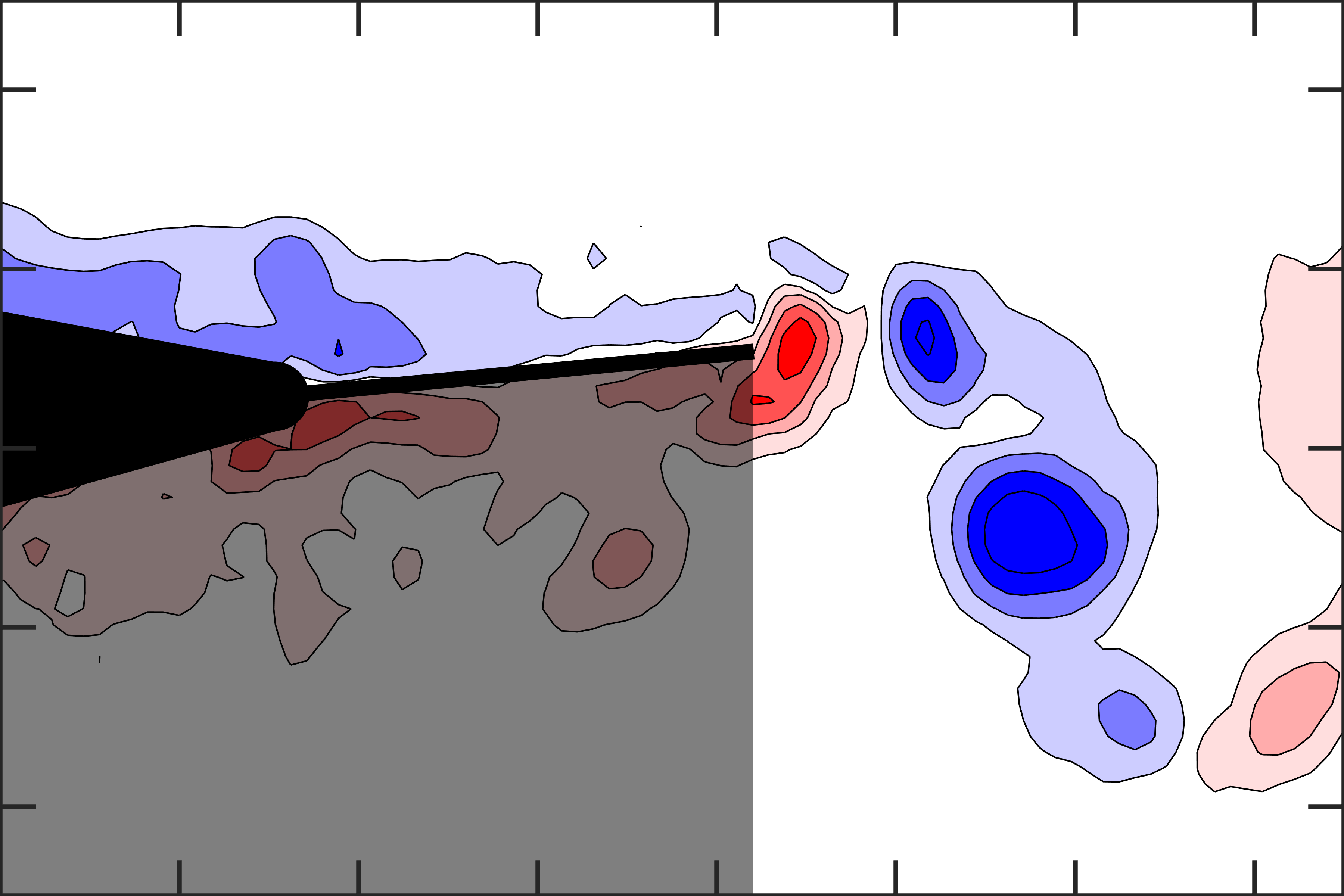

Supplement: Supplementary file 1 [file biomimetics-04-00067-s001.zip › Brooks_Green_Supplemental_Materials/Figures/TEVel_St0p27_T03p03_C05p00_p00mm_pActual14_pRaw10.png]

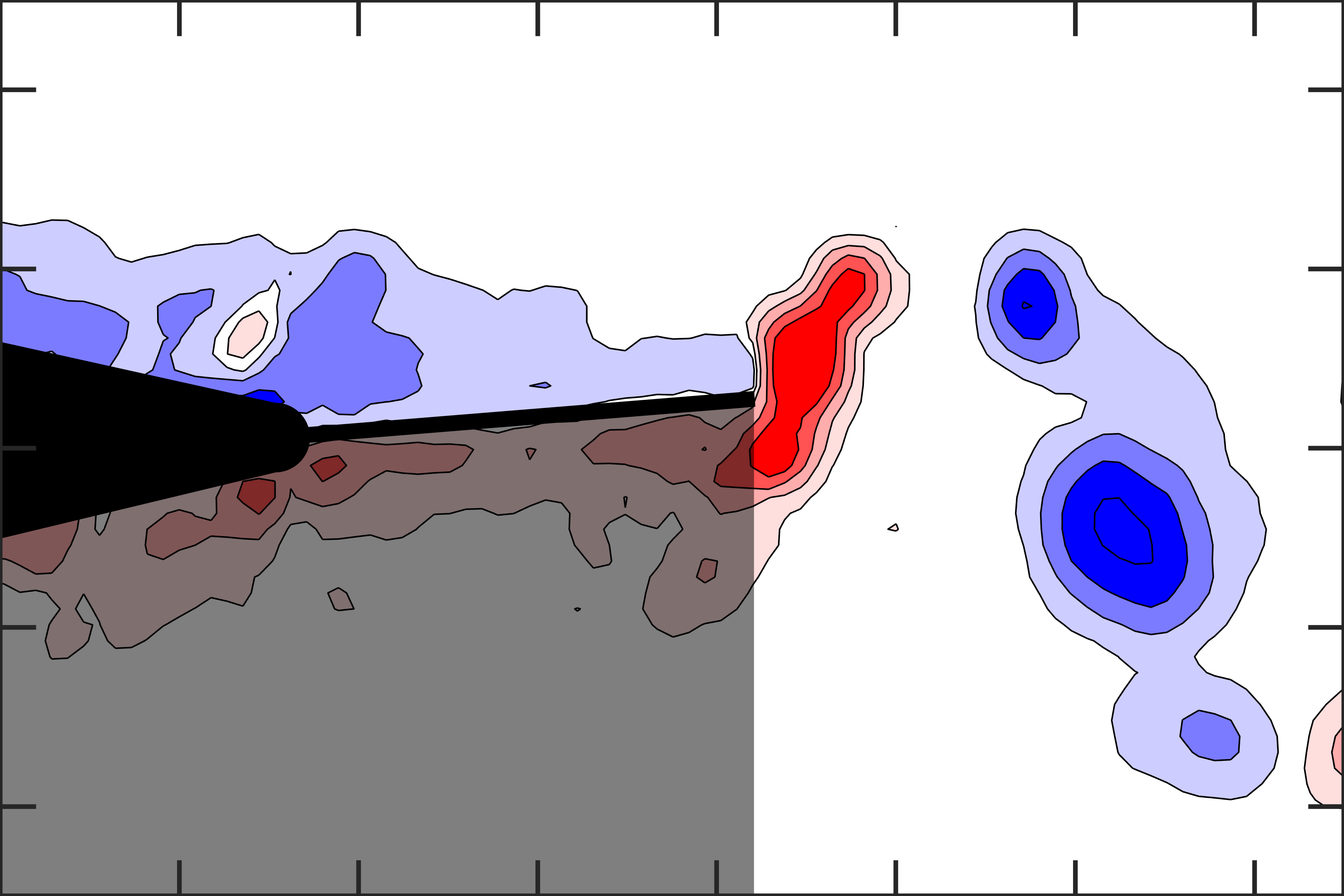

Supplement: Supplementary file 1 [file biomimetics-04-00067-s001.zip › Brooks_Green_Supplemental_Materials/Figures/TEVel_St0p27_T03p03_C05p00_p00mm_pActual17_pRaw13.png]

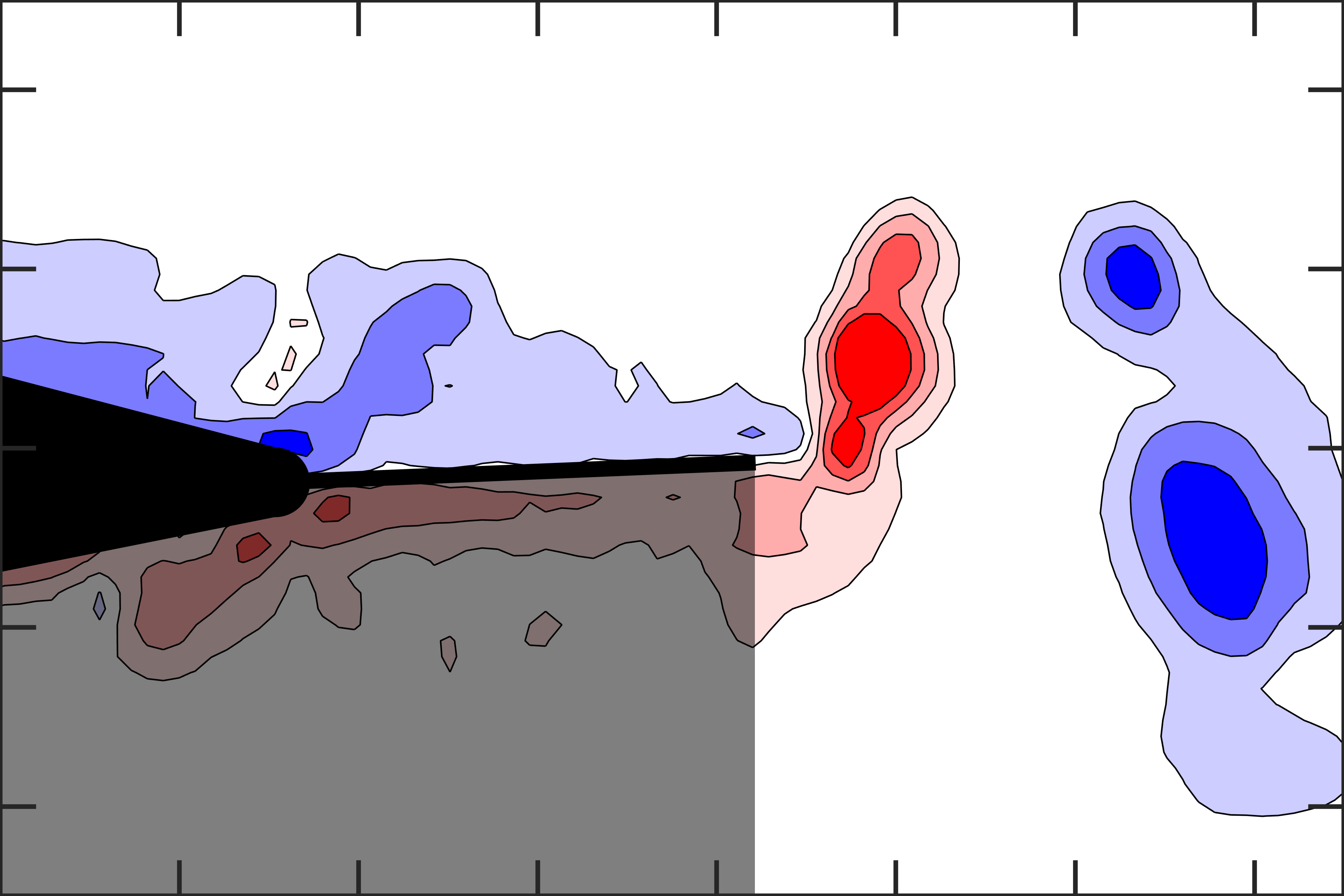

Supplement: Supplementary file 1 [file biomimetics-04-00067-s001.zip › Brooks_Green_Supplemental_Materials/Figures/TEVel_St0p27_T03p03_C05p00_p00mm_pActual20_pRaw16.png]

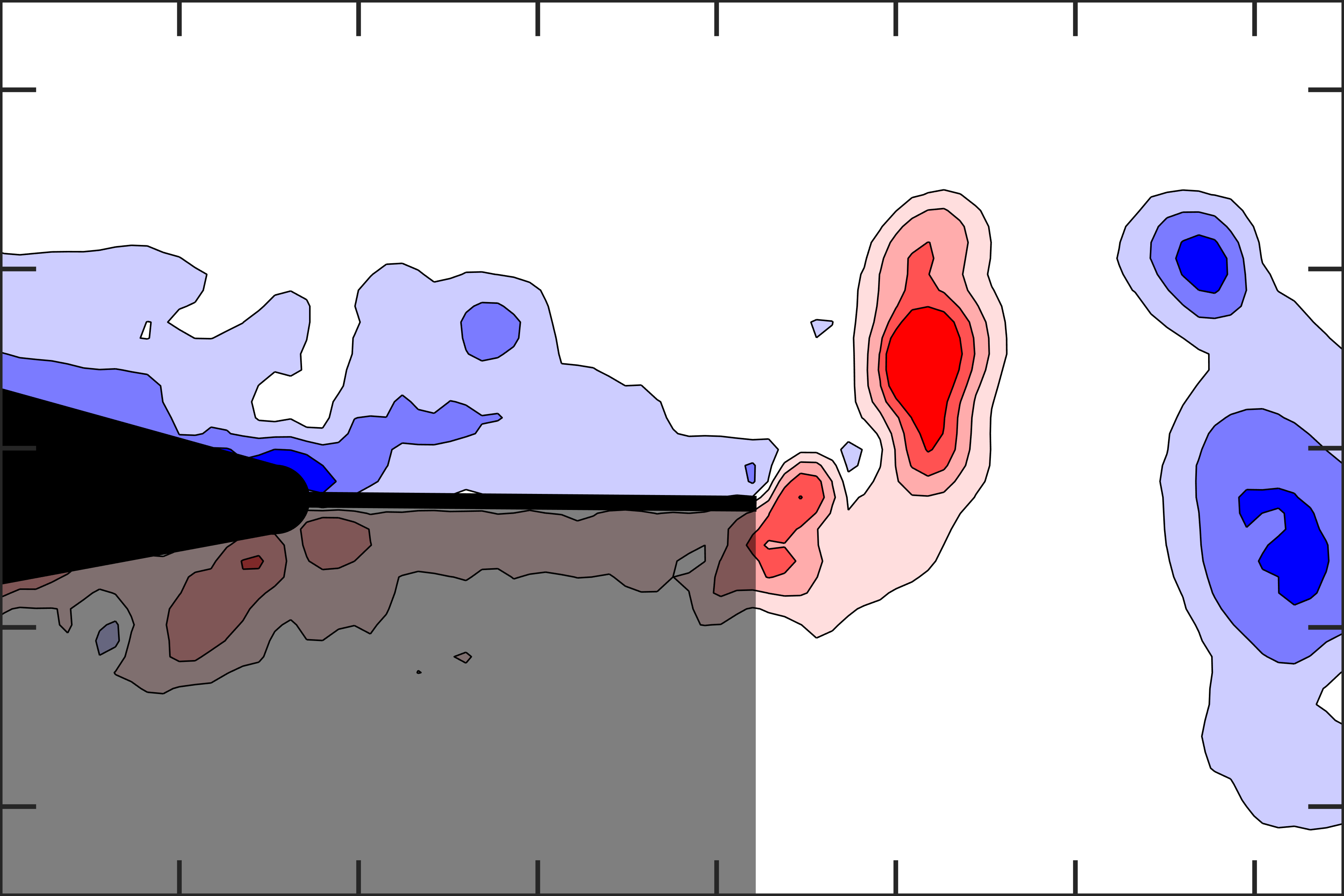

Supplement: Supplementary file 1 [file biomimetics-04-00067-s001.zip › Brooks_Green_Supplemental_Materials/Figures/TEVel_St0p27_T03p03_C05p00_p00mm_pActual22_pRaw18.png]

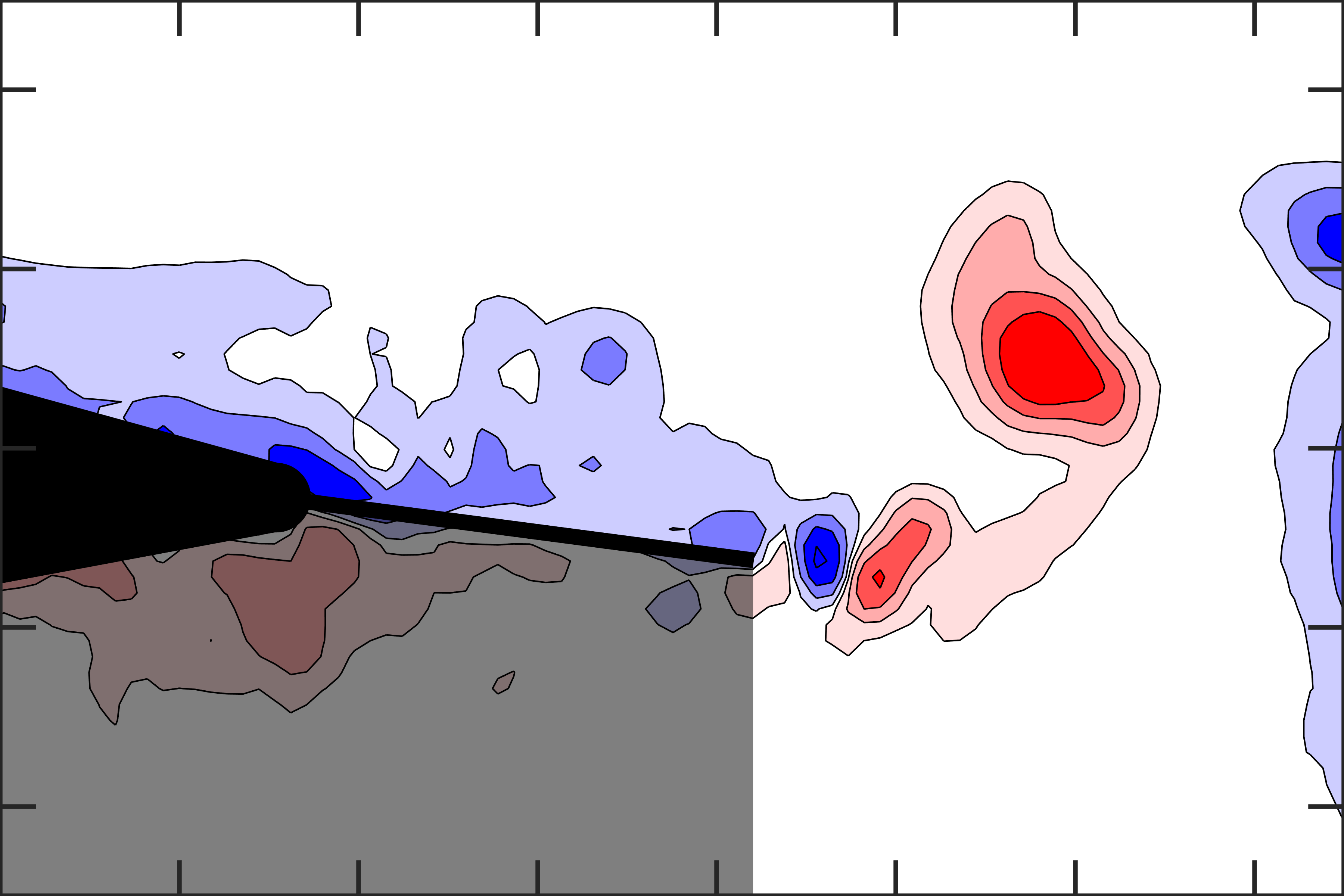

Supplement: Supplementary file 1 [file biomimetics-04-00067-s001.zip › Brooks_Green_Supplemental_Materials/Figures/TEVel_St0p27_T03p03_C05p00_p00mm_pActual26_pRaw22.png]

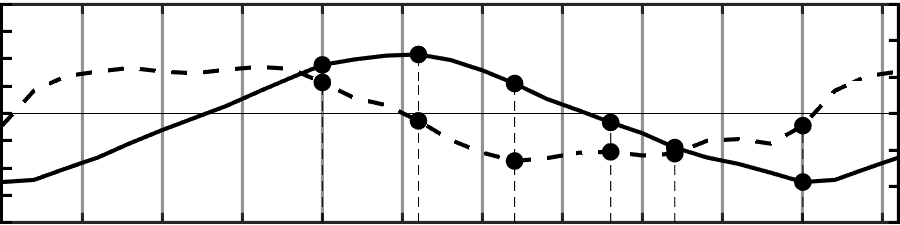

Supplement: Supplementary file 1 [file biomimetics-04-00067-s001.zip › Brooks_Green_Supplemental_Materials/Figures/TEVel_St0p27_T03p03_C05p00_p00mm_Velocity.png]

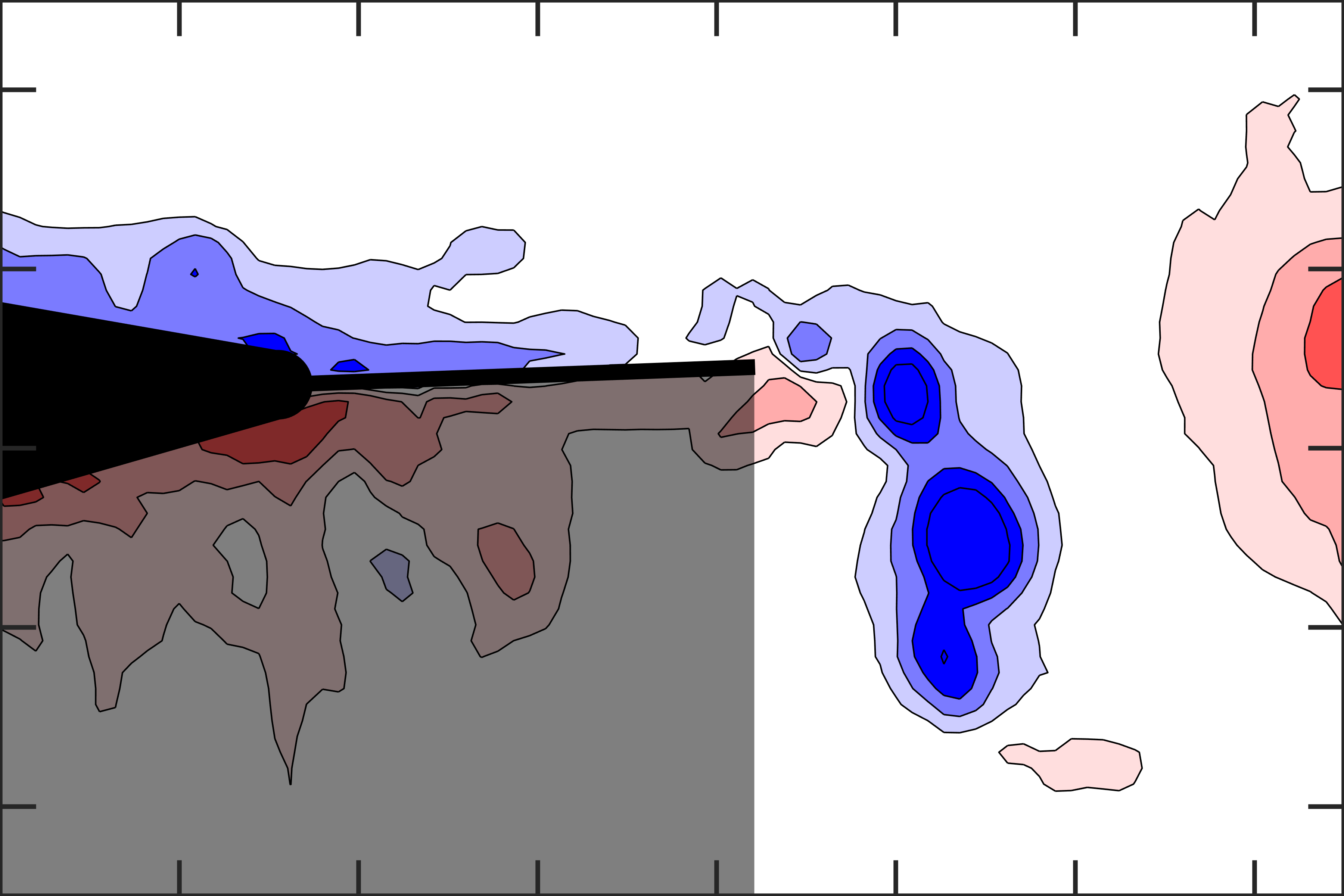

Supplement: Supplementary file 1 [file biomimetics-04-00067-s001.zip › Brooks_Green_Supplemental_Materials/Figures/TEVel_St0p27_T03p64_C00p00_p00mm_pActual11_pRaw05.png]

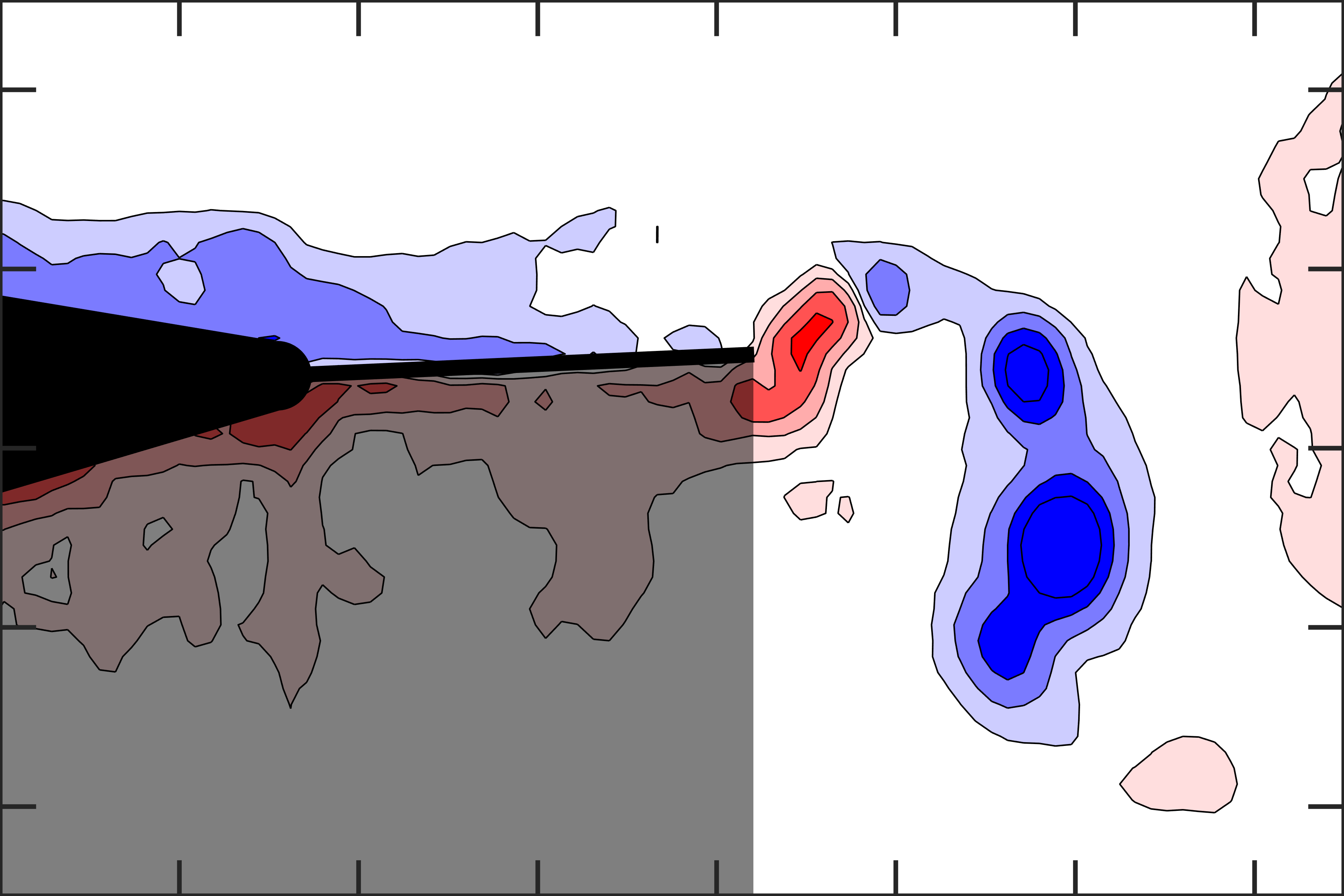

Supplement: Supplementary file 1 [file biomimetics-04-00067-s001.zip › Brooks_Green_Supplemental_Materials/Figures/TEVel_St0p27_T03p64_C00p00_p00mm_pActual14_pRaw08.png]

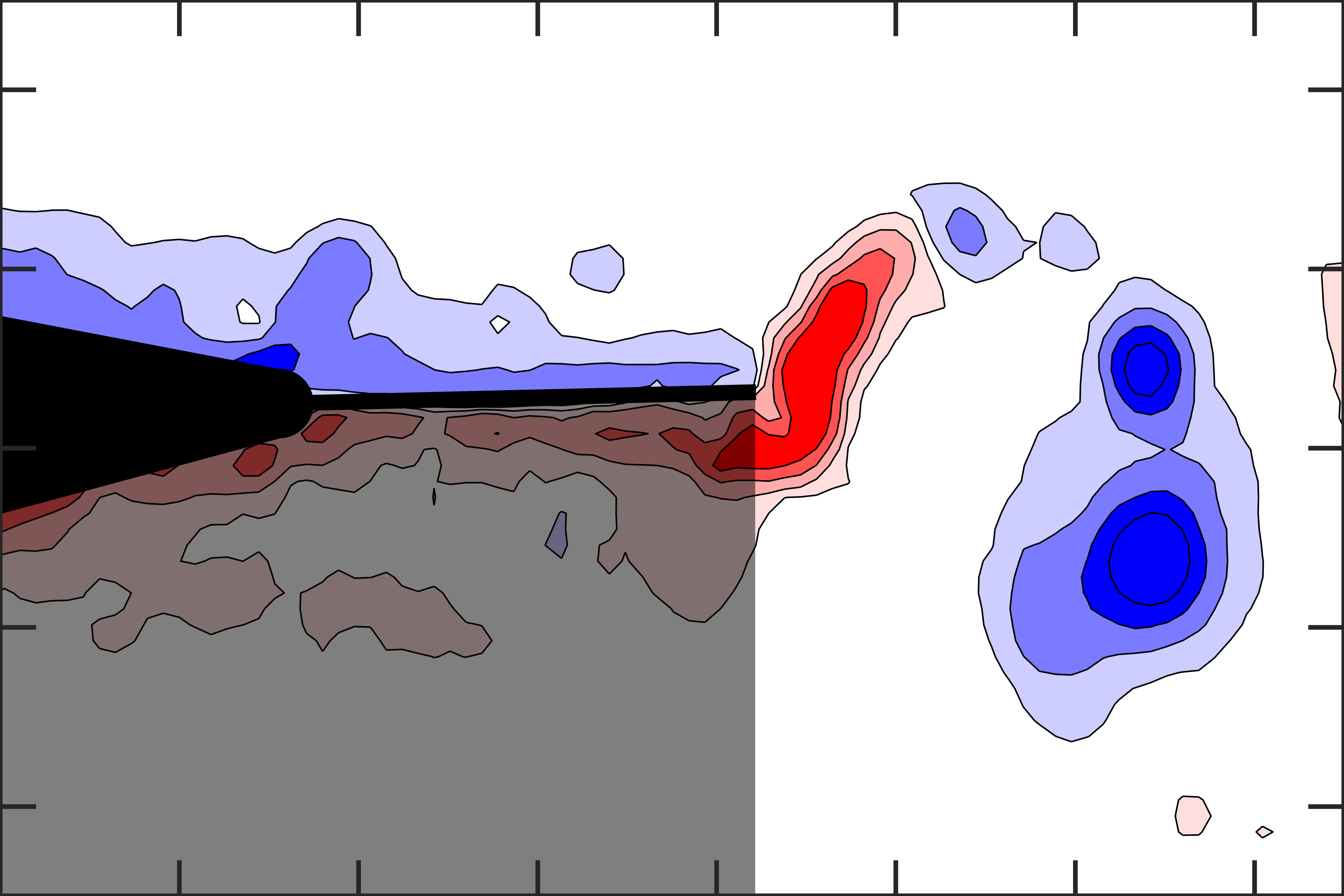

Supplement: Supplementary file 1 [file biomimetics-04-00067-s001.zip › Brooks_Green_Supplemental_Materials/Figures/TEVel_St0p27_T03p64_C00p00_p00mm_pActual17_pRaw11.png]

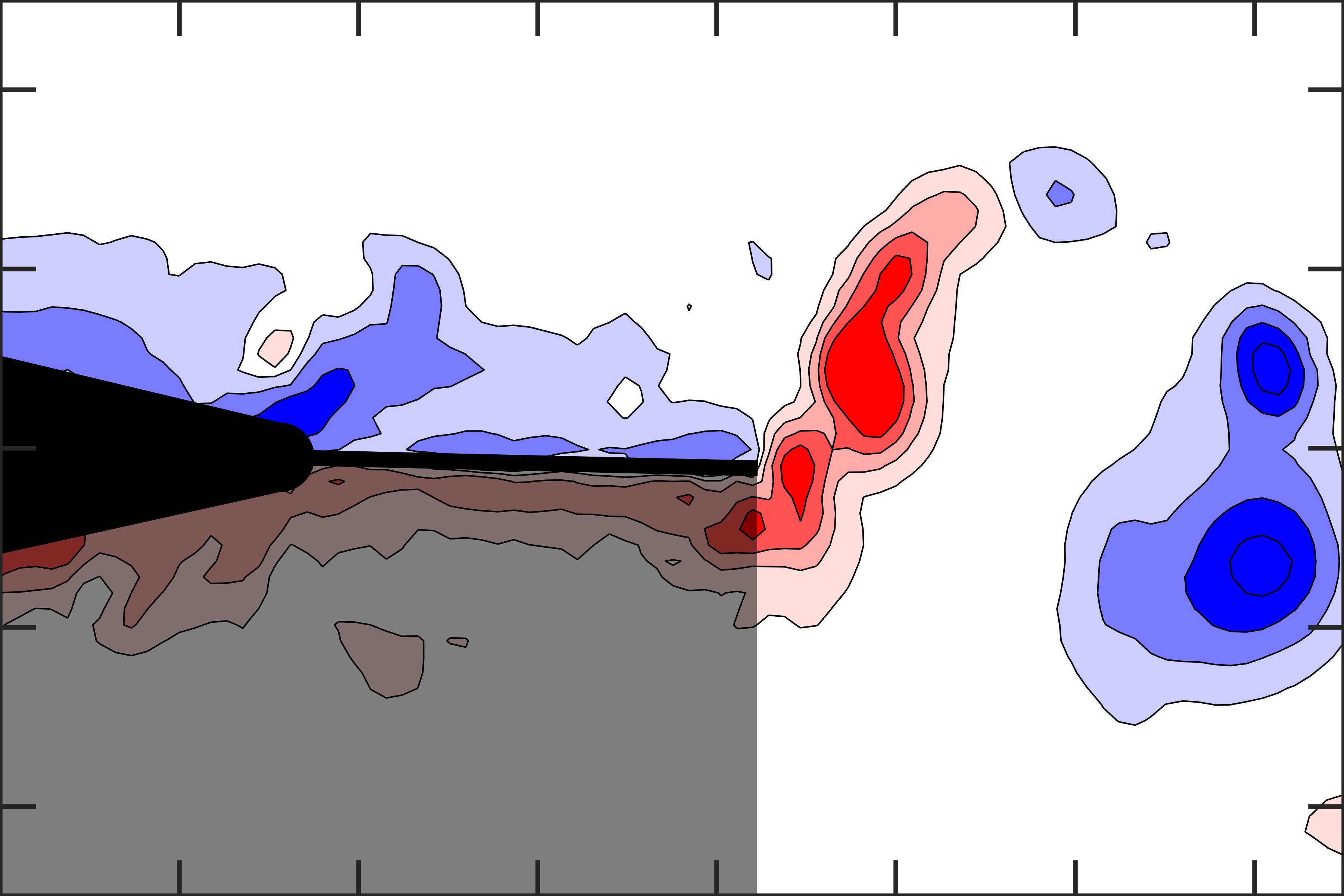

Supplement: Supplementary file 1 [file biomimetics-04-00067-s001.zip › Brooks_Green_Supplemental_Materials/Figures/TEVel_St0p27_T03p64_C00p00_p00mm_pActual20_pRaw14.png]

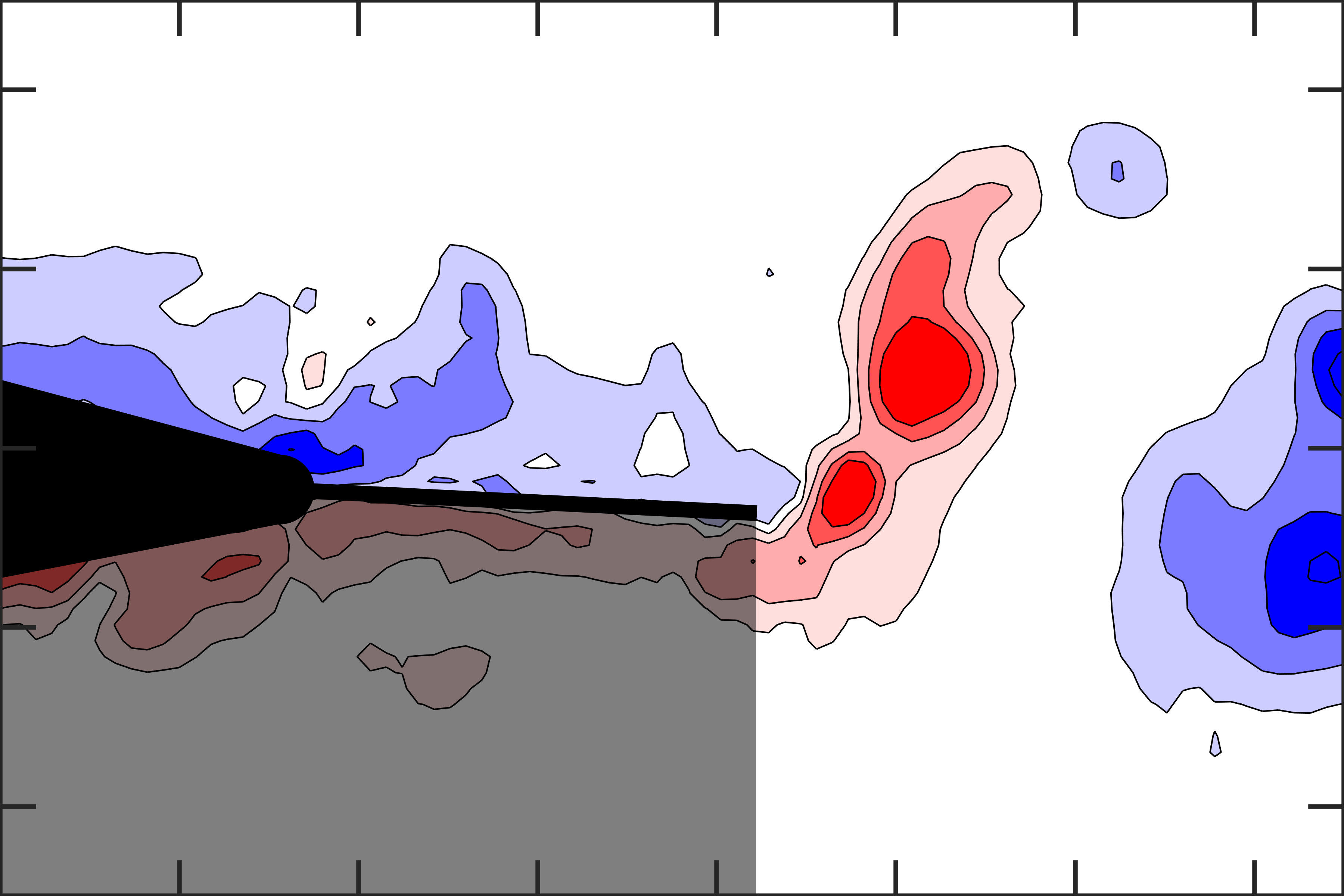

Supplement: Supplementary file 1 [file biomimetics-04-00067-s001.zip › Brooks_Green_Supplemental_Materials/Figures/TEVel_St0p27_T03p64_C00p00_p00mm_pActual22_pRaw16.png]

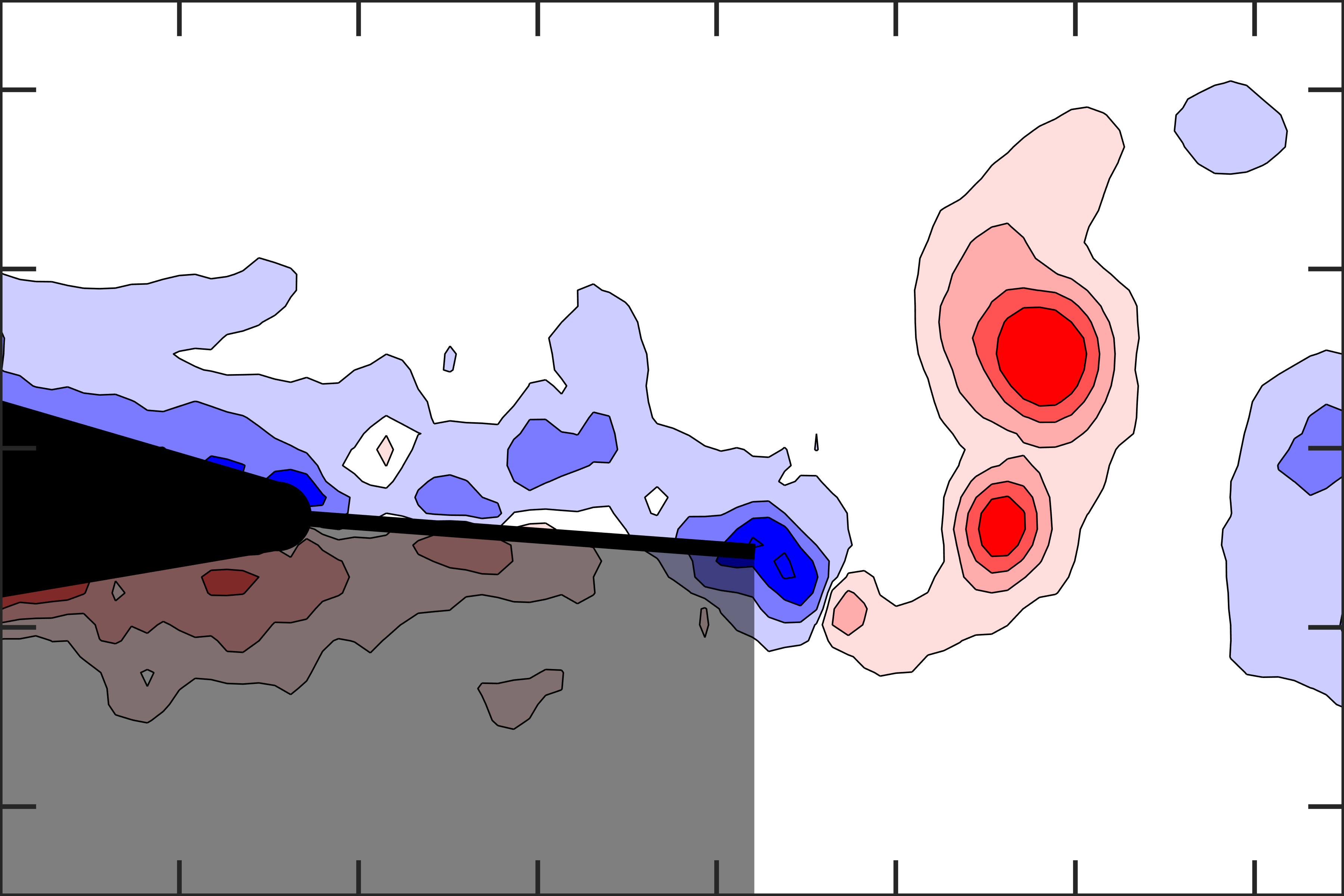

Supplement: Supplementary file 1 [file biomimetics-04-00067-s001.zip › Brooks_Green_Supplemental_Materials/Figures/TEVel_St0p27_T03p64_C00p00_p00mm_pActual26_pRaw20.png]

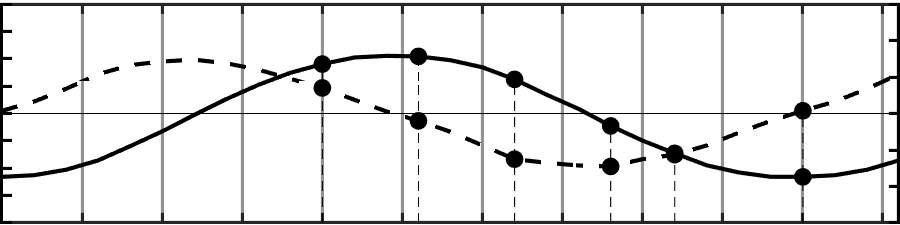

Supplement: Supplementary file 1 [file biomimetics-04-00067-s001.zip › Brooks_Green_Supplemental_Materials/Figures/TEVel_St0p27_T03p64_C00p00_p00mm_Velocity.png]

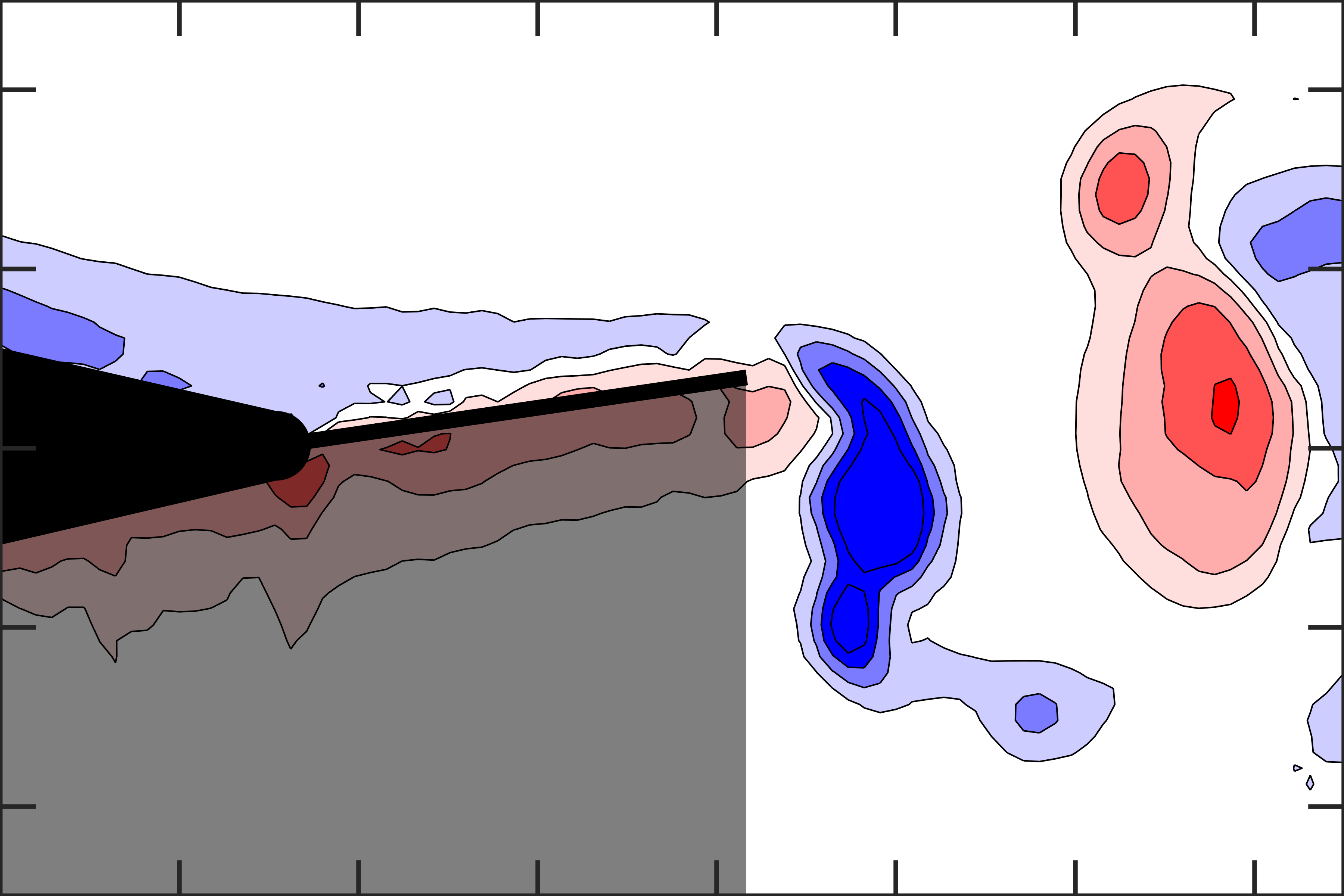

Supplement: Supplementary file 1 [file biomimetics-04-00067-s001.zip › Brooks_Green_Supplemental_Materials/Figures/TEVel_St0p37_T00p00_C15p00_p00mm_pActual11_pRaw10.png]

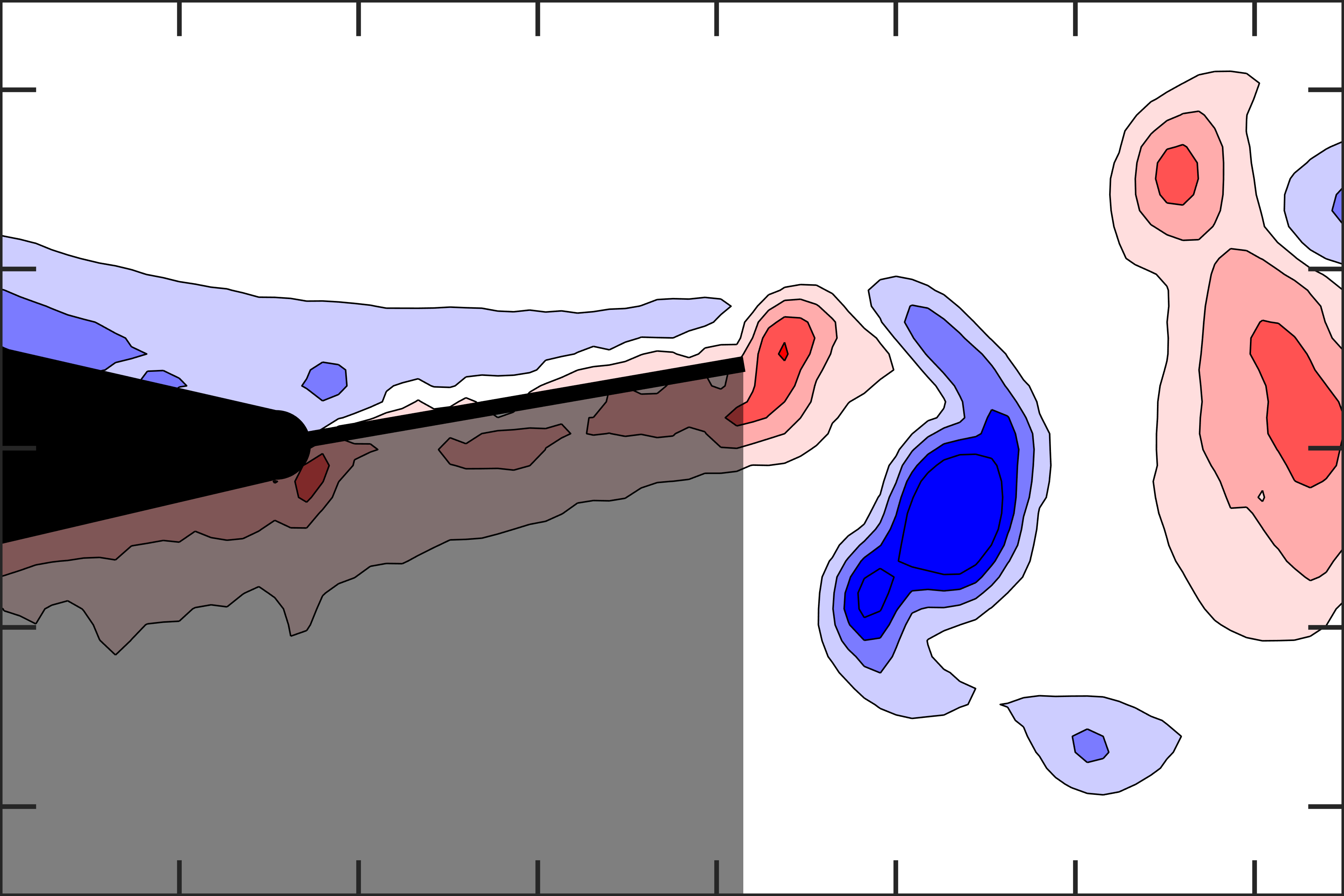

Supplement: Supplementary file 1 [file biomimetics-04-00067-s001.zip › Brooks_Green_Supplemental_Materials/Figures/TEVel_St0p37_T00p00_C15p00_p00mm_pActual14_pRaw13.png]

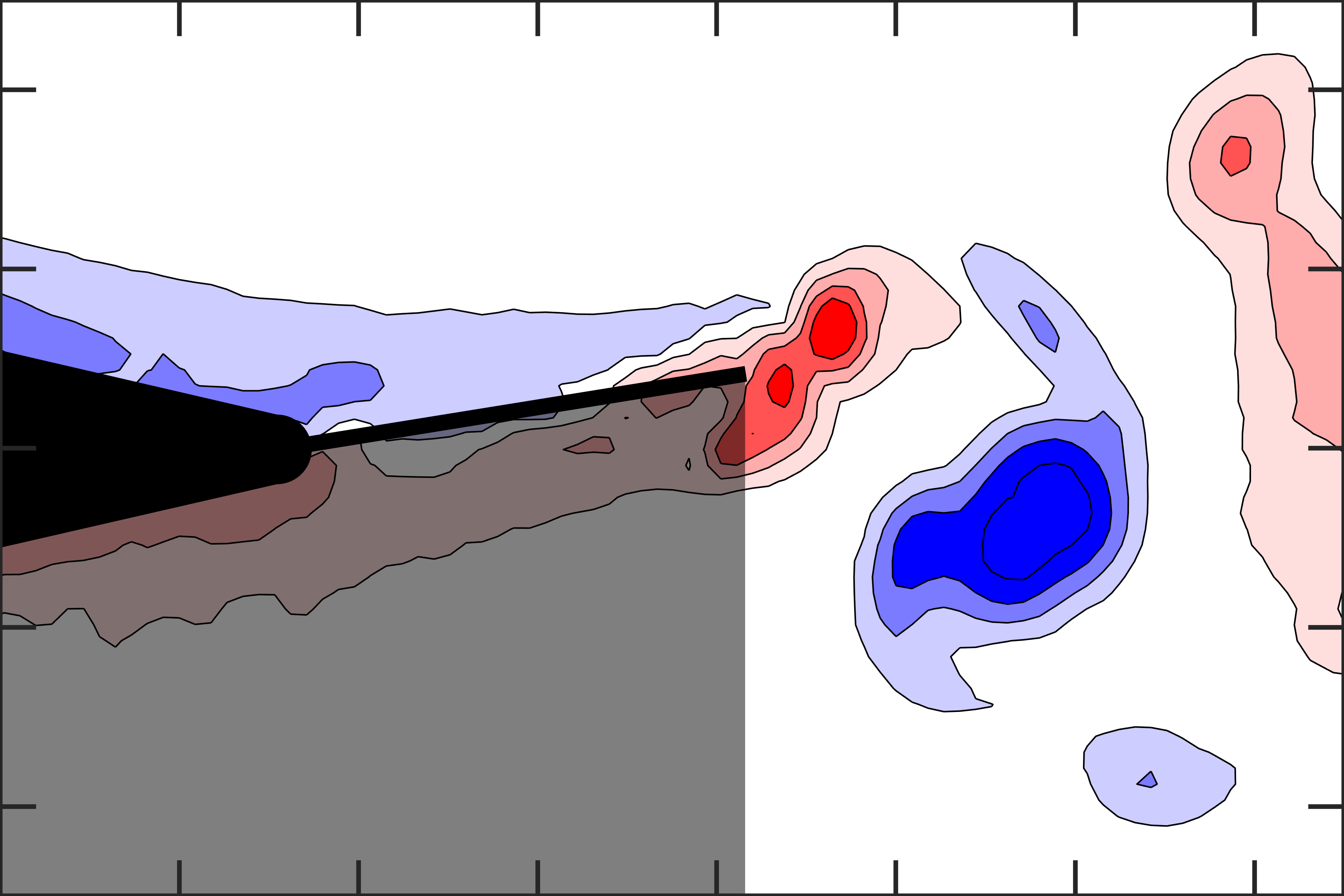

Supplement: Supplementary file 1 [file biomimetics-04-00067-s001.zip › Brooks_Green_Supplemental_Materials/Figures/TEVel_St0p37_T00p00_C15p00_p00mm_pActual17_pRaw16.png]

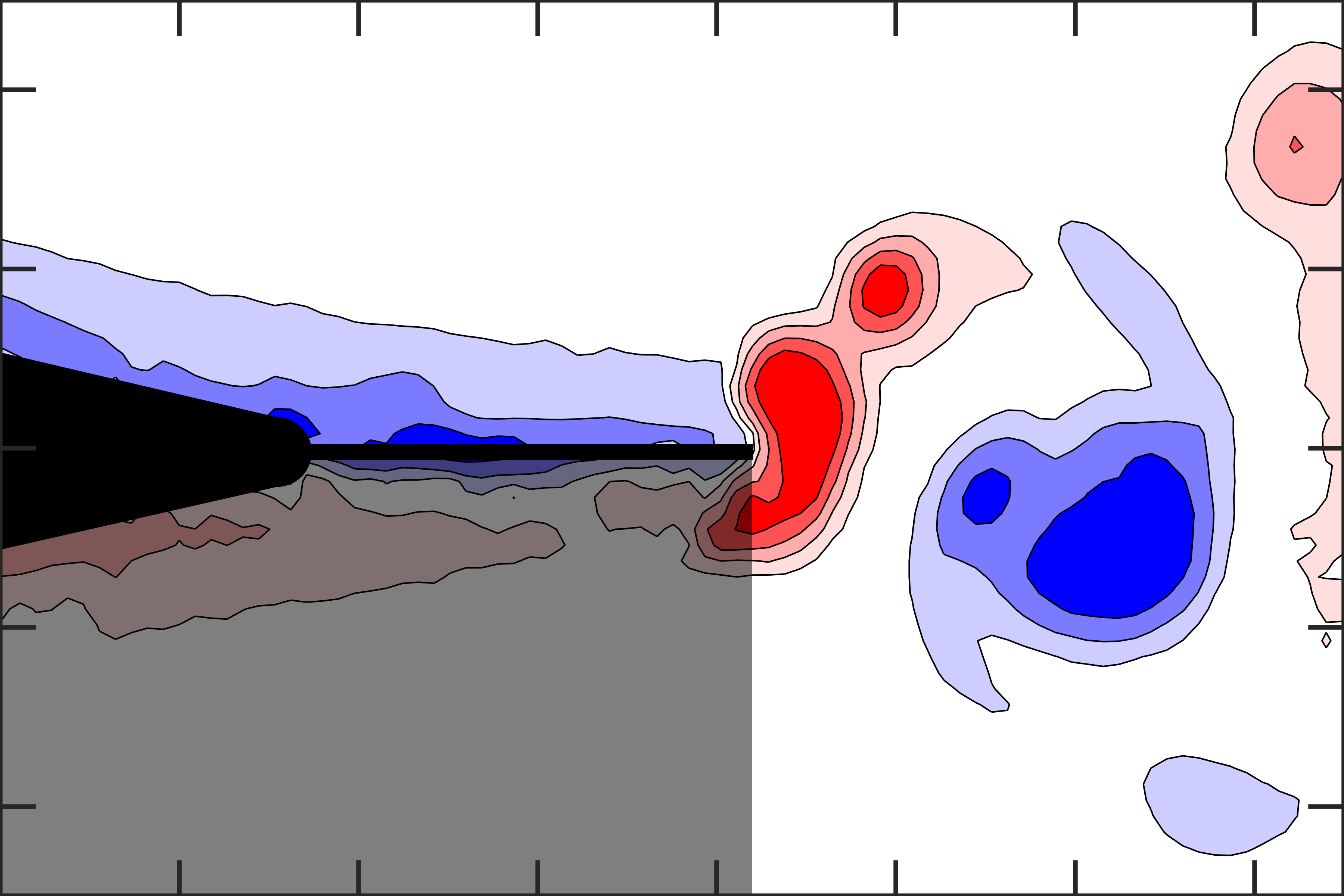

Supplement: Supplementary file 1 [file biomimetics-04-00067-s001.zip › Brooks_Green_Supplemental_Materials/Figures/TEVel_St0p37_T00p00_C15p00_p00mm_pActual20_pRaw19.png]

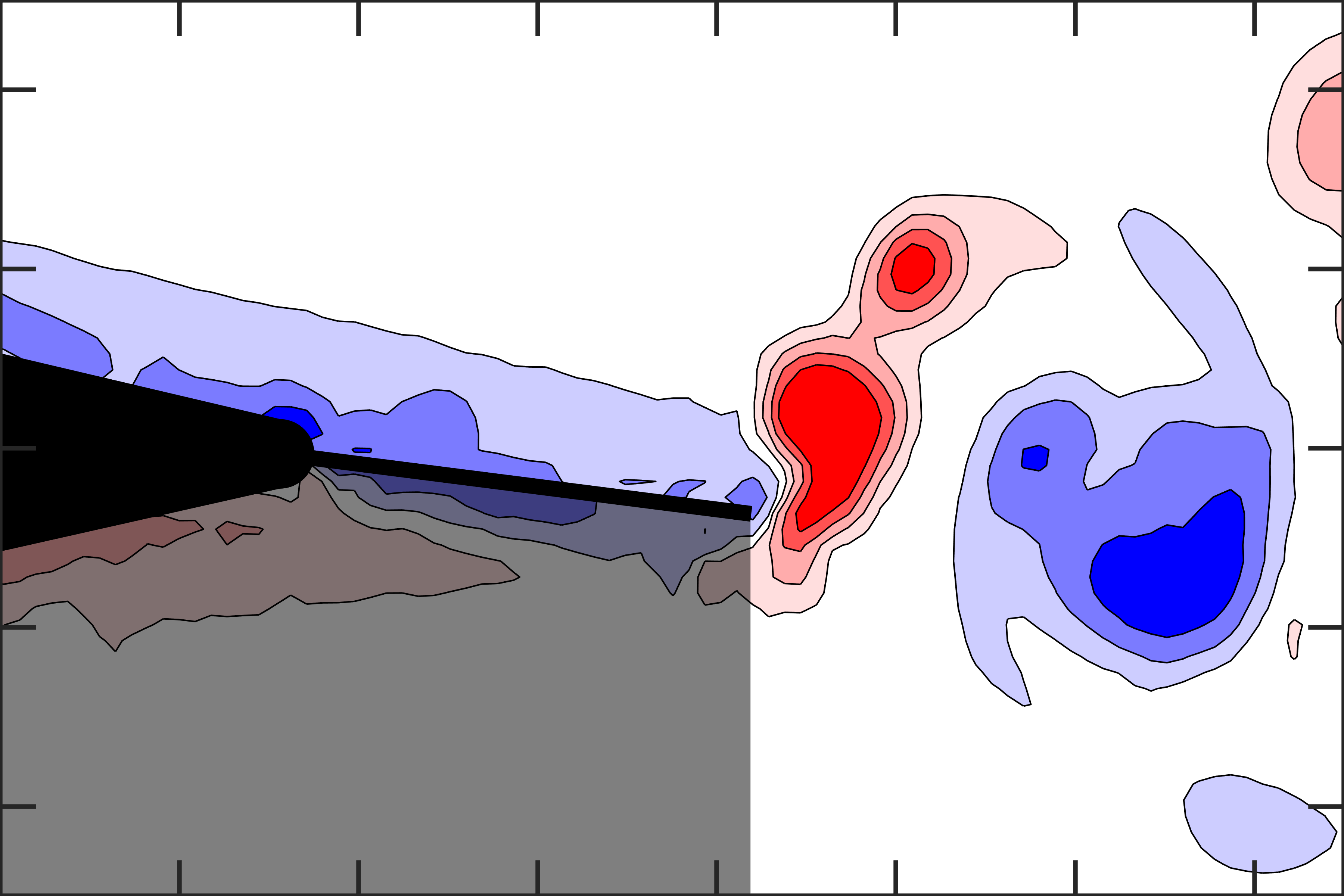

Supplement: Supplementary file 1 [file biomimetics-04-00067-s001.zip › Brooks_Green_Supplemental_Materials/Figures/TEVel_St0p37_T00p00_C15p00_p00mm_pActual22_pRaw21.png]

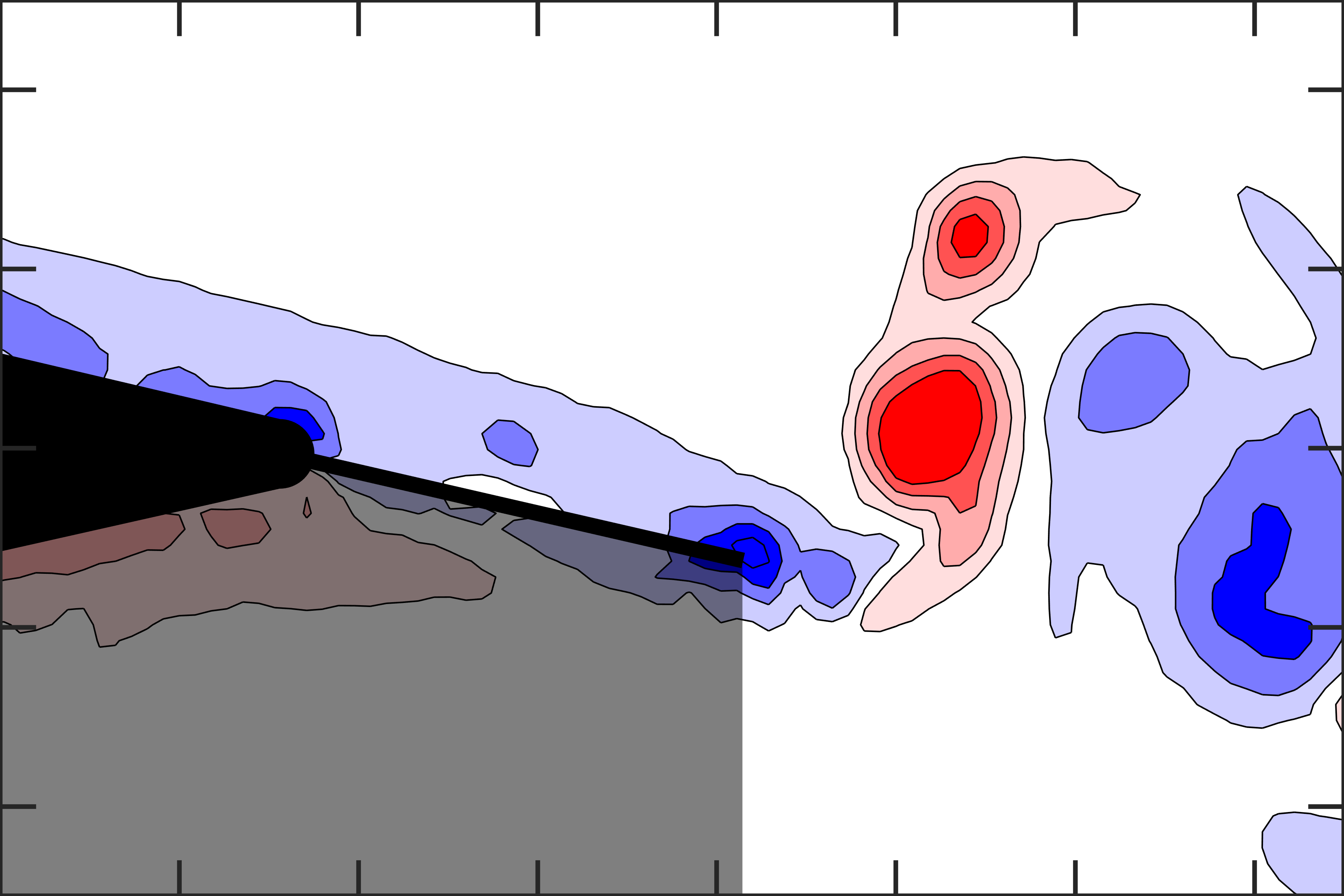

Supplement: Supplementary file 1 [file biomimetics-04-00067-s001.zip › Brooks_Green_Supplemental_Materials/Figures/TEVel_St0p37_T00p00_C15p00_p00mm_pActual26_pRaw25.png]

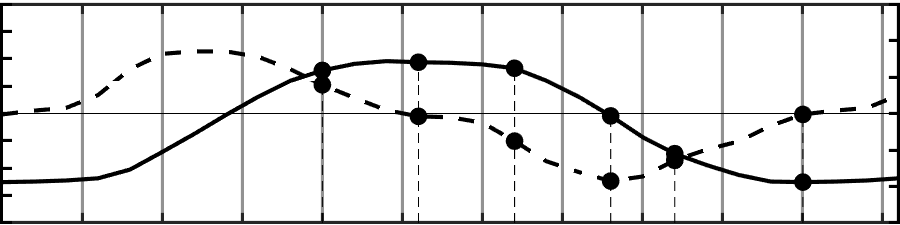

Supplement: Supplementary file 1 [file biomimetics-04-00067-s001.zip › Brooks_Green_Supplemental_Materials/Figures/TEVel_St0p37_T00p00_C15p00_p00mm_Velocity.png]

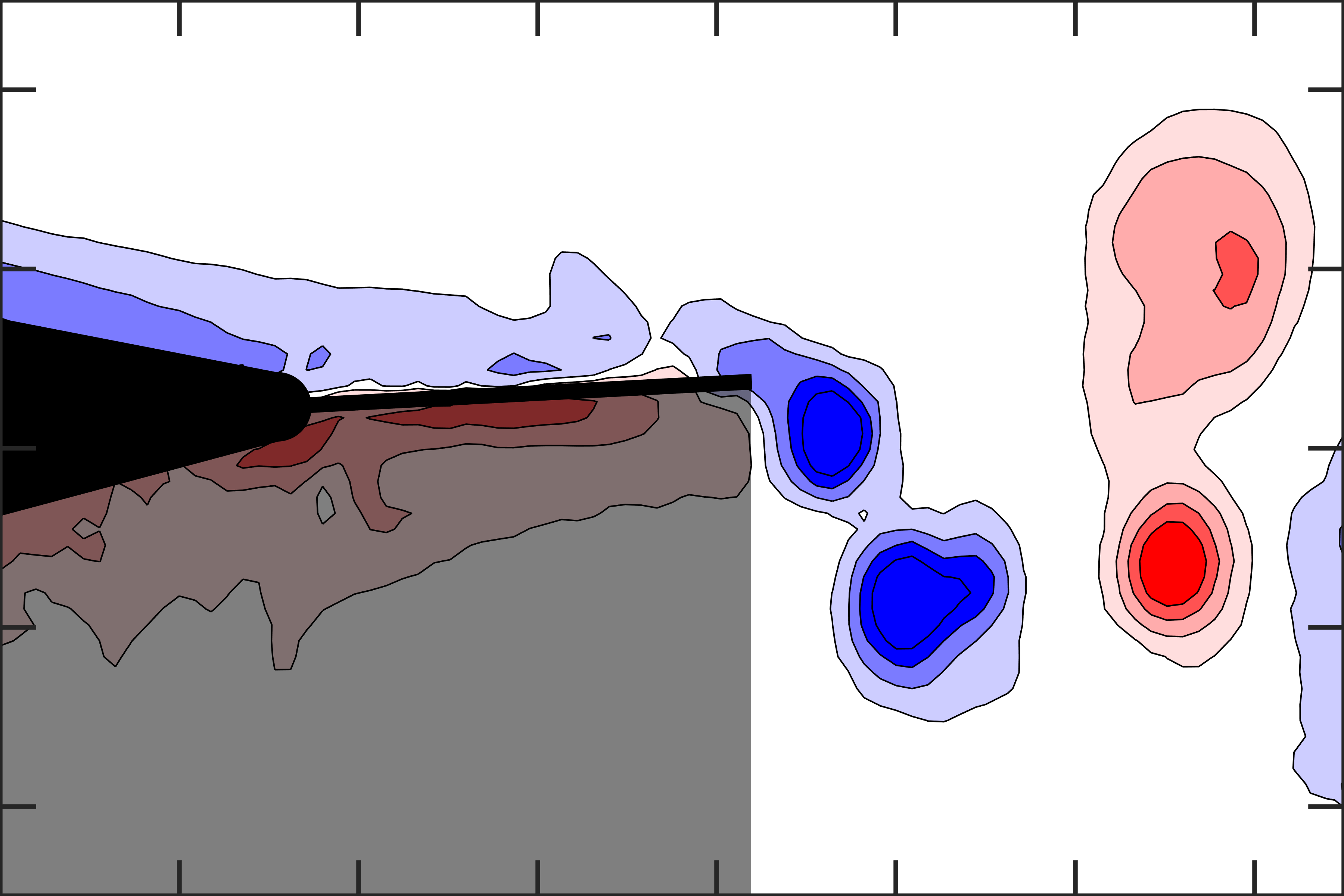

Supplement: Supplementary file 1 [file biomimetics-04-00067-s001.zip › Brooks_Green_Supplemental_Materials/Figures/TEVel_St0p37_T01p99_C10p00_p00mm_pActual11_pRaw08.png]

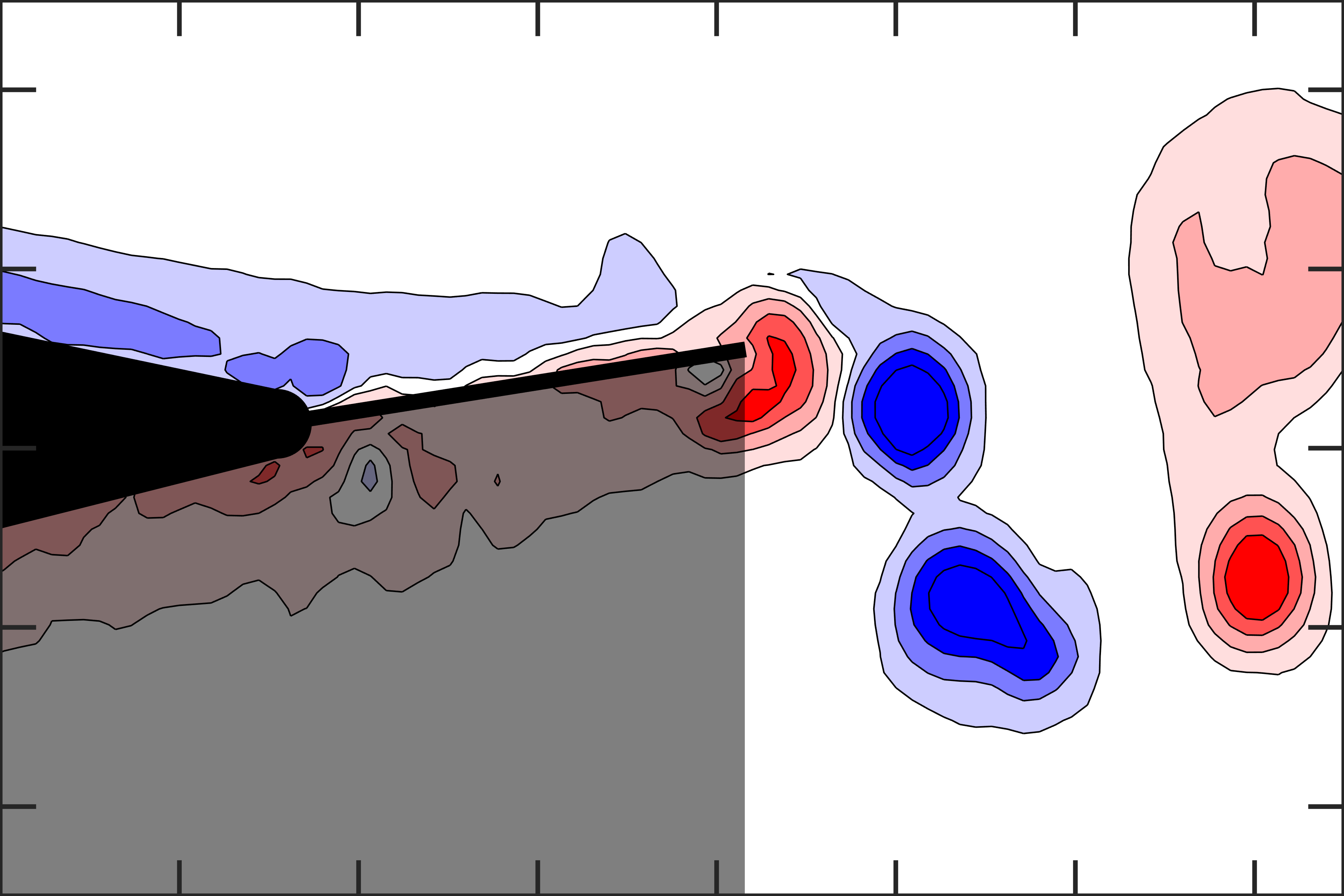

Supplement: Supplementary file 1 [file biomimetics-04-00067-s001.zip › Brooks_Green_Supplemental_Materials/Figures/TEVel_St0p37_T01p99_C10p00_p00mm_pActual14_pRaw11.png]

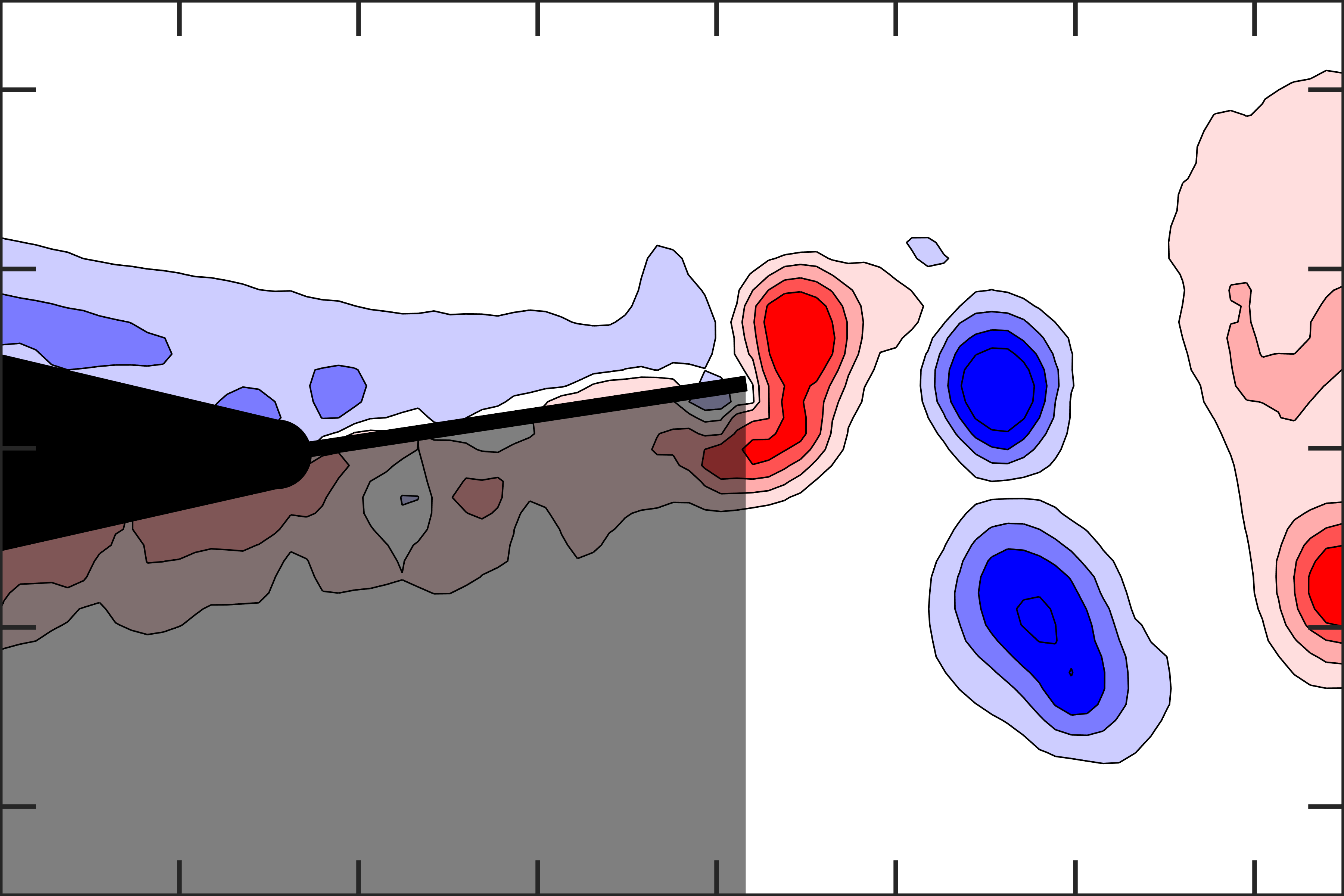

Supplement: Supplementary file 1 [file biomimetics-04-00067-s001.zip › Brooks_Green_Supplemental_Materials/Figures/TEVel_St0p37_T01p99_C10p00_p00mm_pActual17_pRaw14.png]

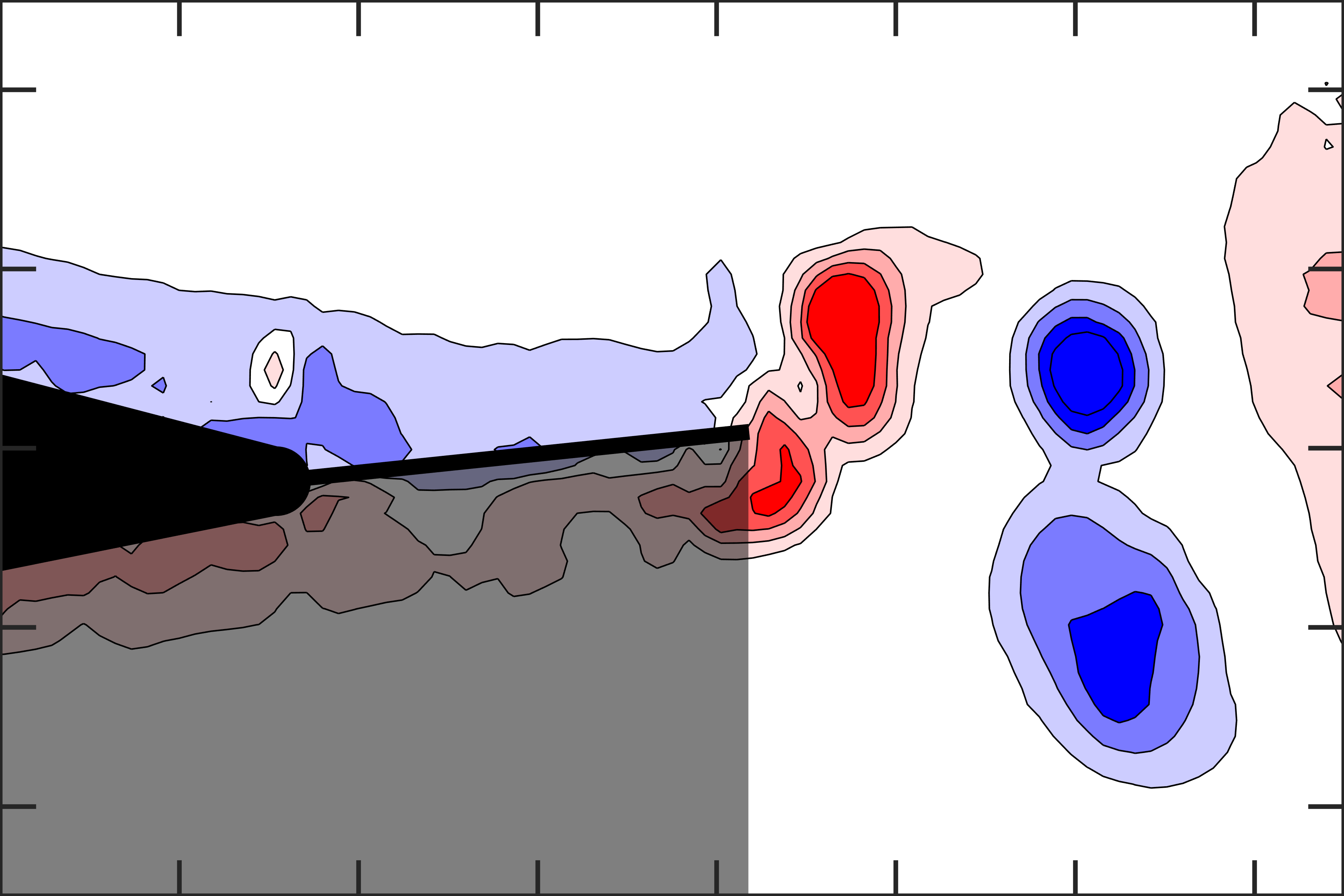

Supplement: Supplementary file 1 [file biomimetics-04-00067-s001.zip › Brooks_Green_Supplemental_Materials/Figures/TEVel_St0p37_T01p99_C10p00_p00mm_pActual20_pRaw17.png]

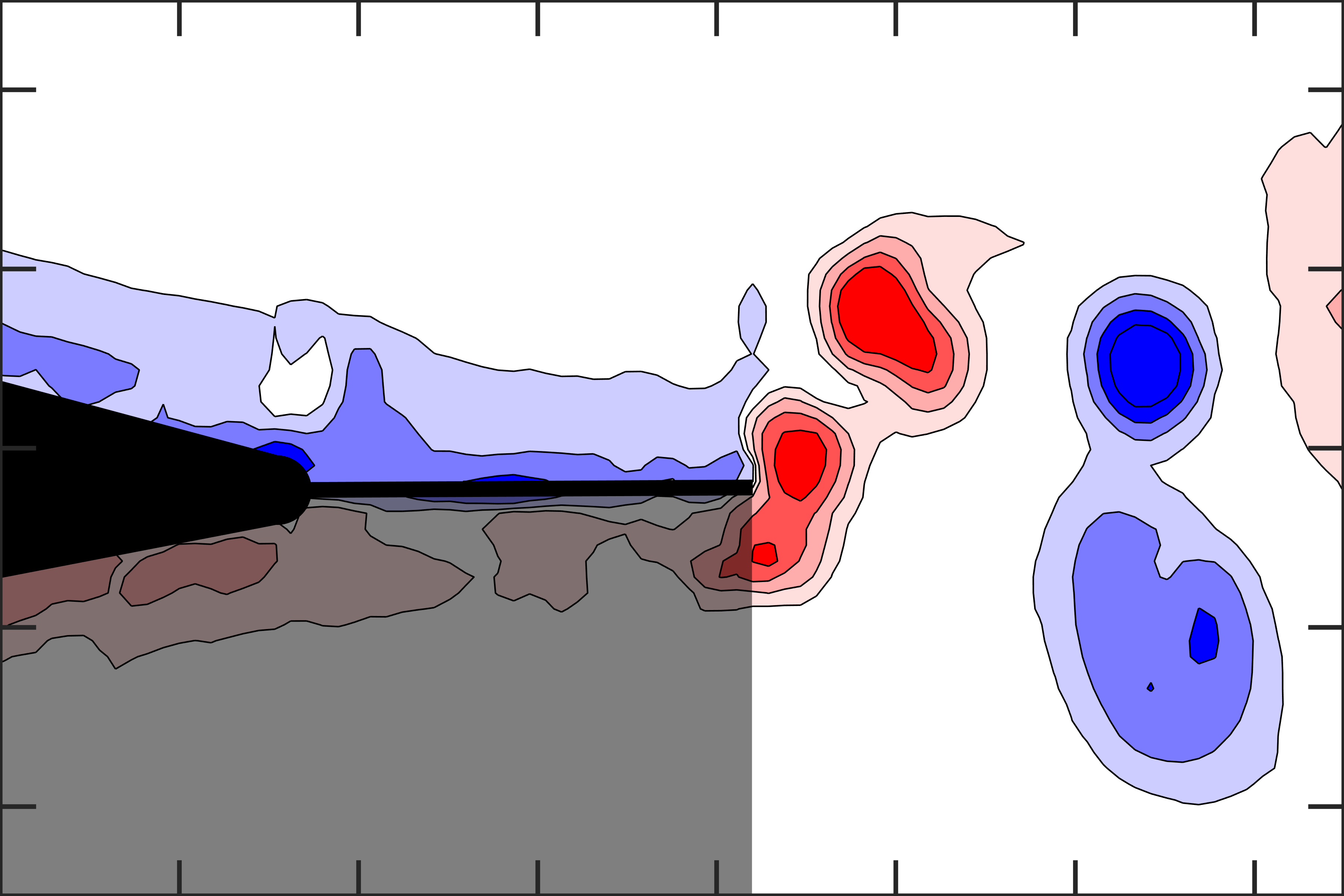

Supplement: Supplementary file 1 [file biomimetics-04-00067-s001.zip › Brooks_Green_Supplemental_Materials/Figures/TEVel_St0p37_T01p99_C10p00_p00mm_pActual22_pRaw19.png]

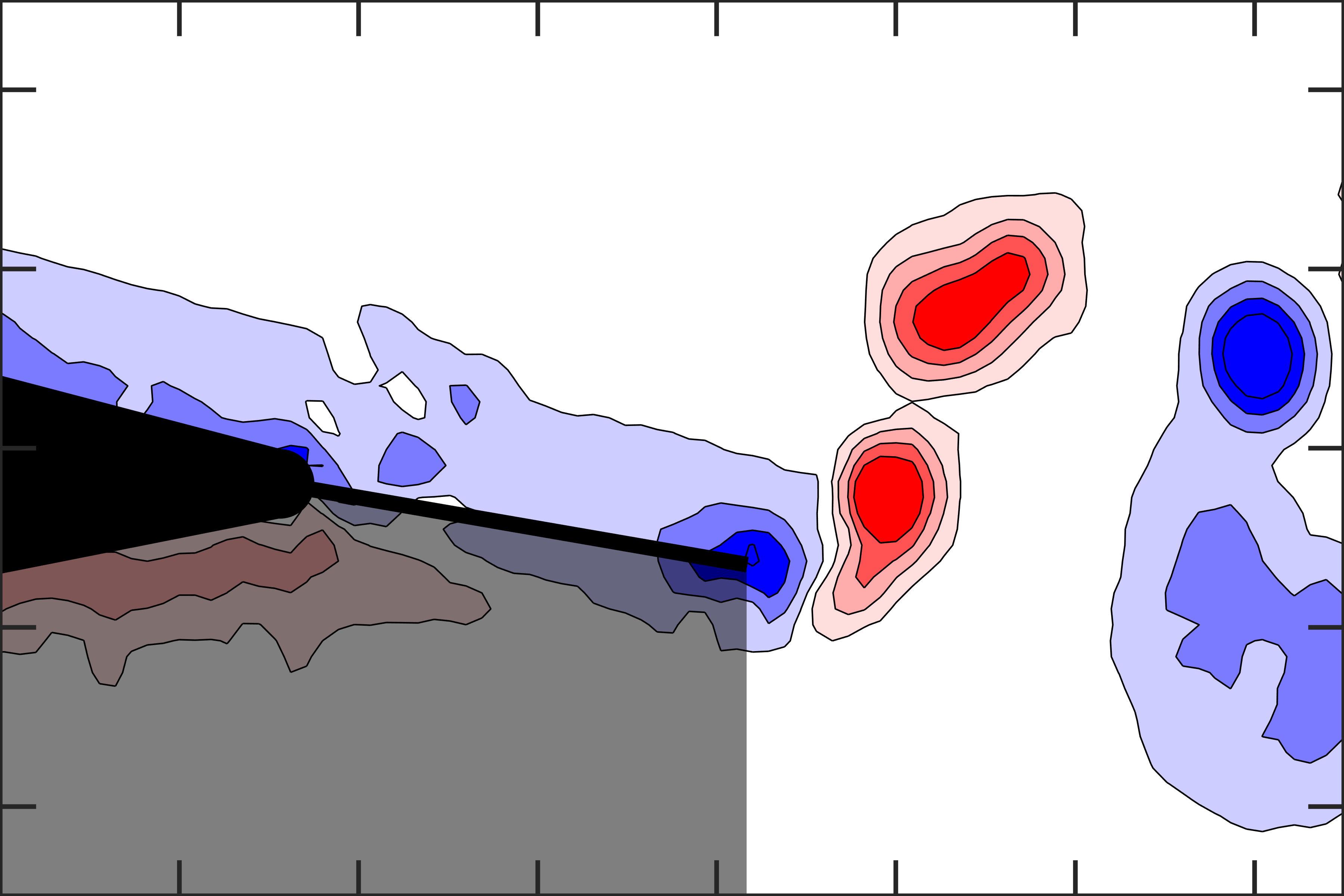

Supplement: Supplementary file 1 [file biomimetics-04-00067-s001.zip › Brooks_Green_Supplemental_Materials/Figures/TEVel_St0p37_T01p99_C10p00_p00mm_pActual26_pRaw23.png]

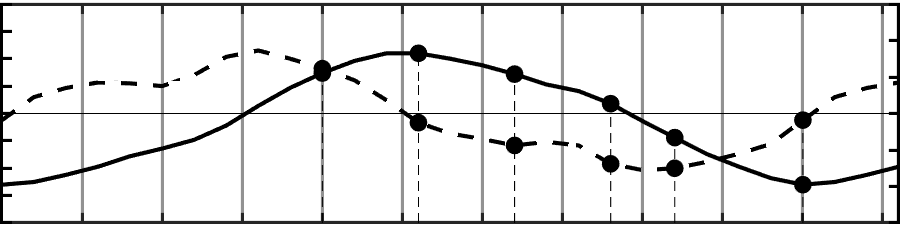

Supplement: Supplementary file 1 [file biomimetics-04-00067-s001.zip › Brooks_Green_Supplemental_Materials/Figures/TEVel_St0p37_T01p99_C10p00_p00mm_Velocity.png]

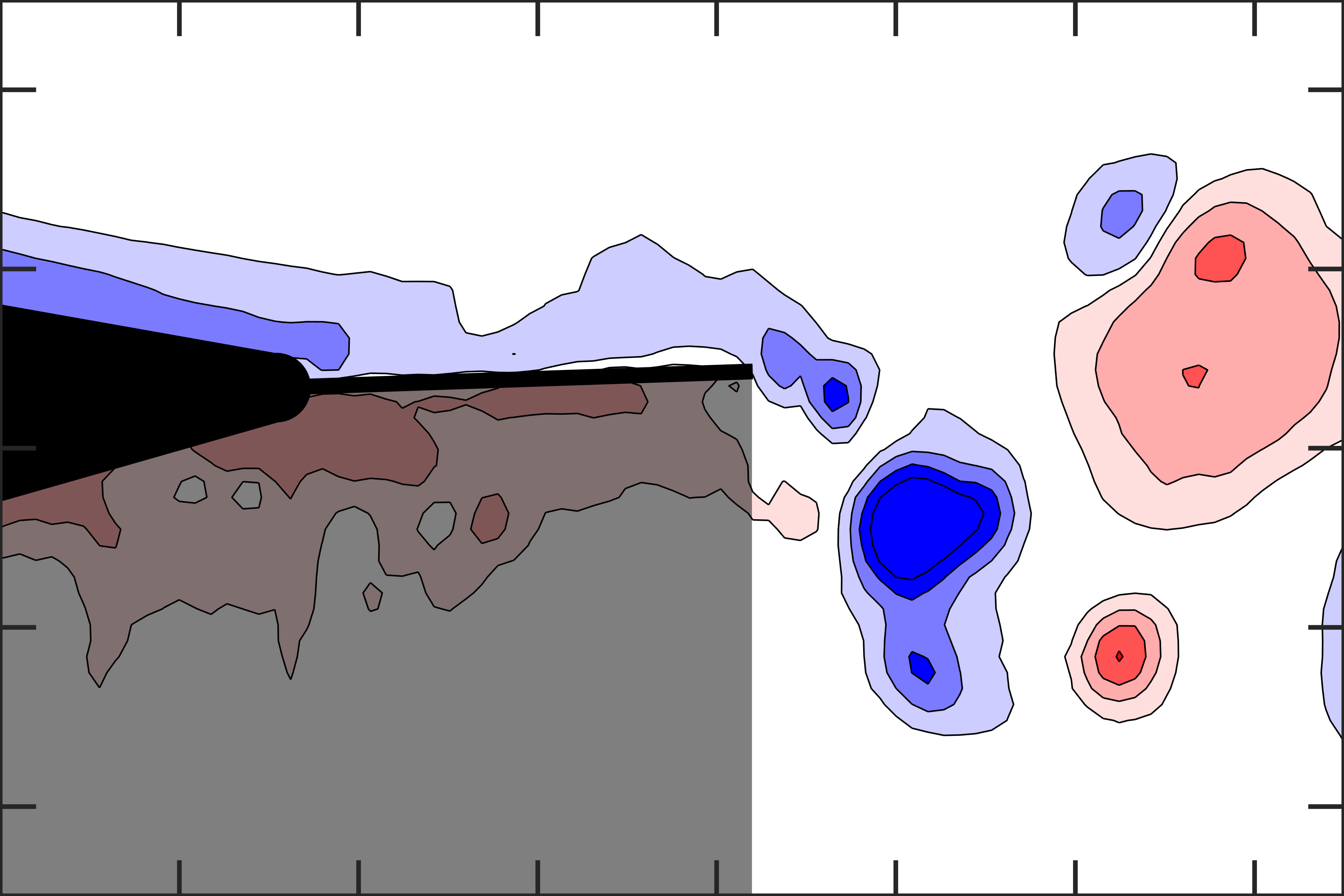

Supplement: Supplementary file 1 [file biomimetics-04-00067-s001.zip › Brooks_Green_Supplemental_Materials/Figures/TEVel_St0p37_T03p03_C05p00_p00mm_pActual11_pRaw07.png]

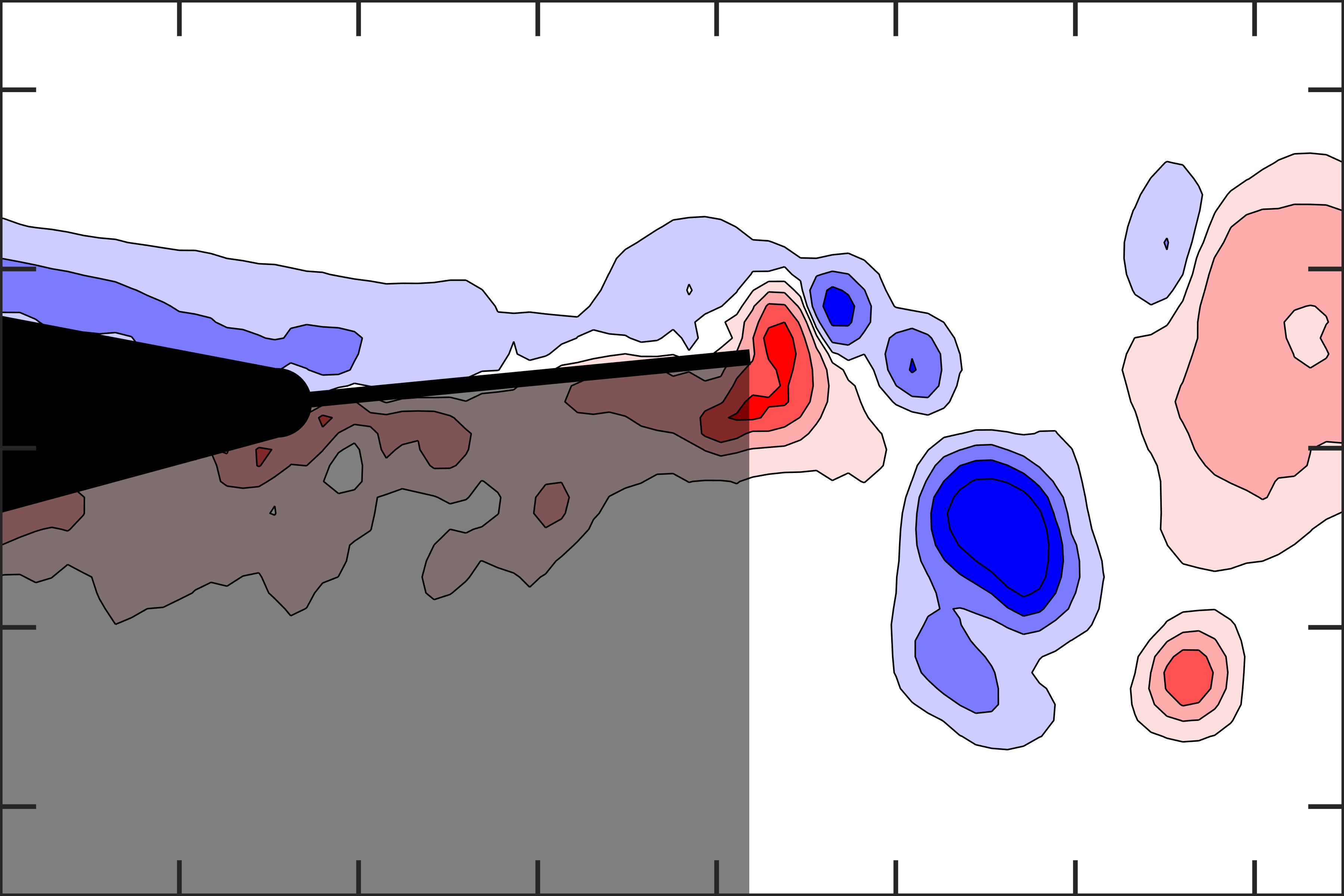

Supplement: Supplementary file 1 [file biomimetics-04-00067-s001.zip › Brooks_Green_Supplemental_Materials/Figures/TEVel_St0p37_T03p03_C05p00_p00mm_pActual14_pRaw10.png]

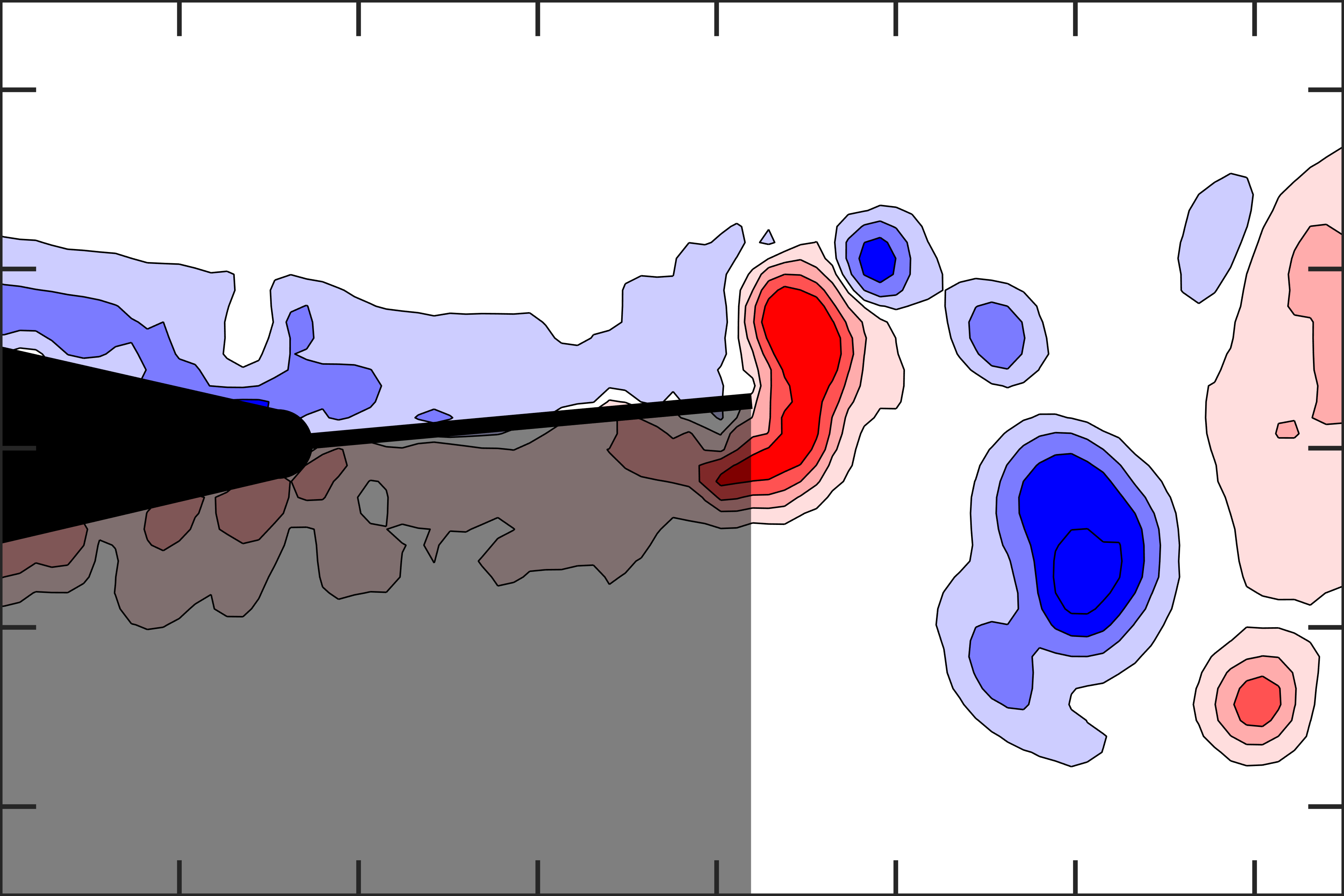

Supplement: Supplementary file 1 [file biomimetics-04-00067-s001.zip › Brooks_Green_Supplemental_Materials/Figures/TEVel_St0p37_T03p03_C05p00_p00mm_pActual17_pRaw13.png]

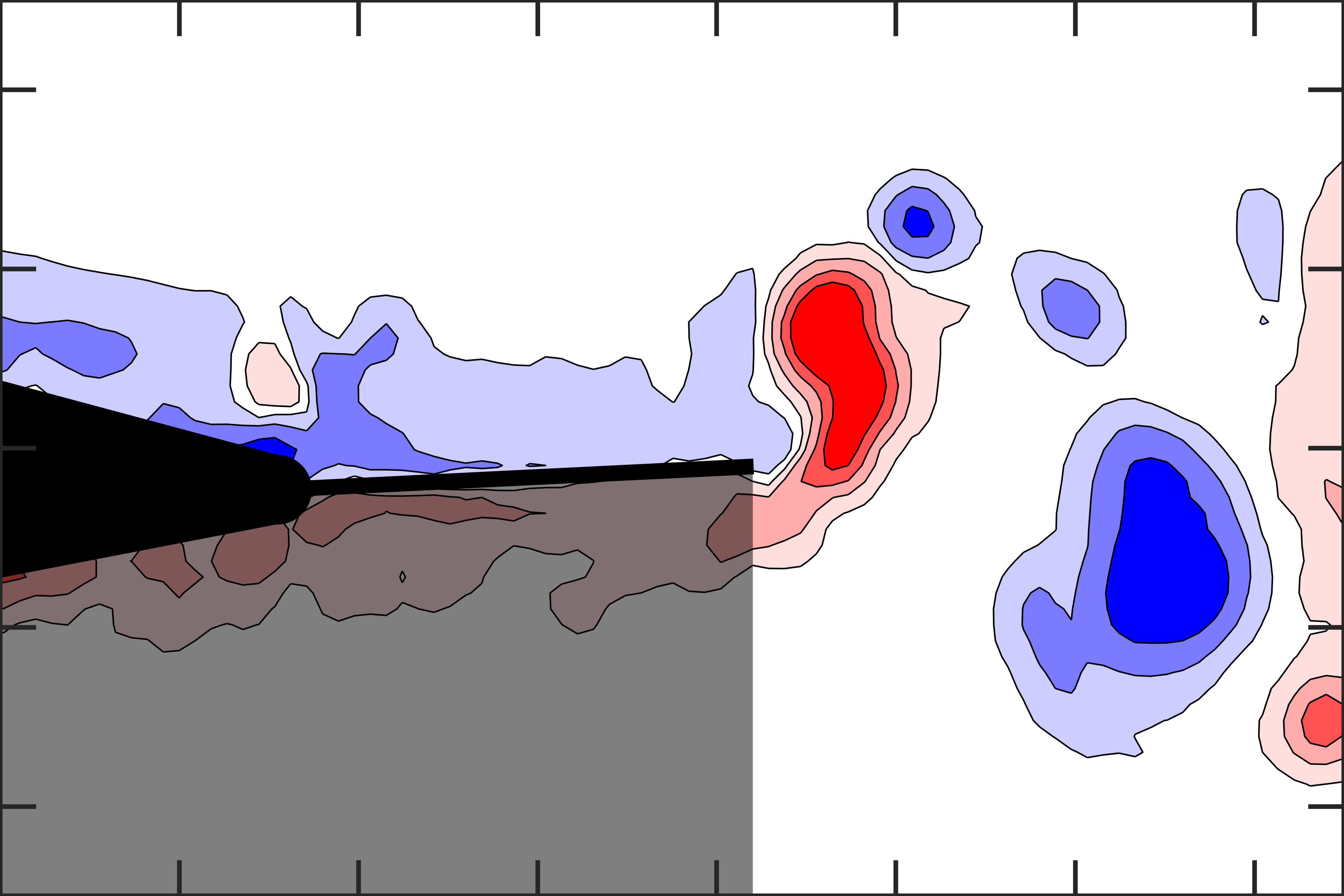

Supplement: Supplementary file 1 [file biomimetics-04-00067-s001.zip › Brooks_Green_Supplemental_Materials/Figures/TEVel_St0p37_T03p03_C05p00_p00mm_pActual20_pRaw16.png]

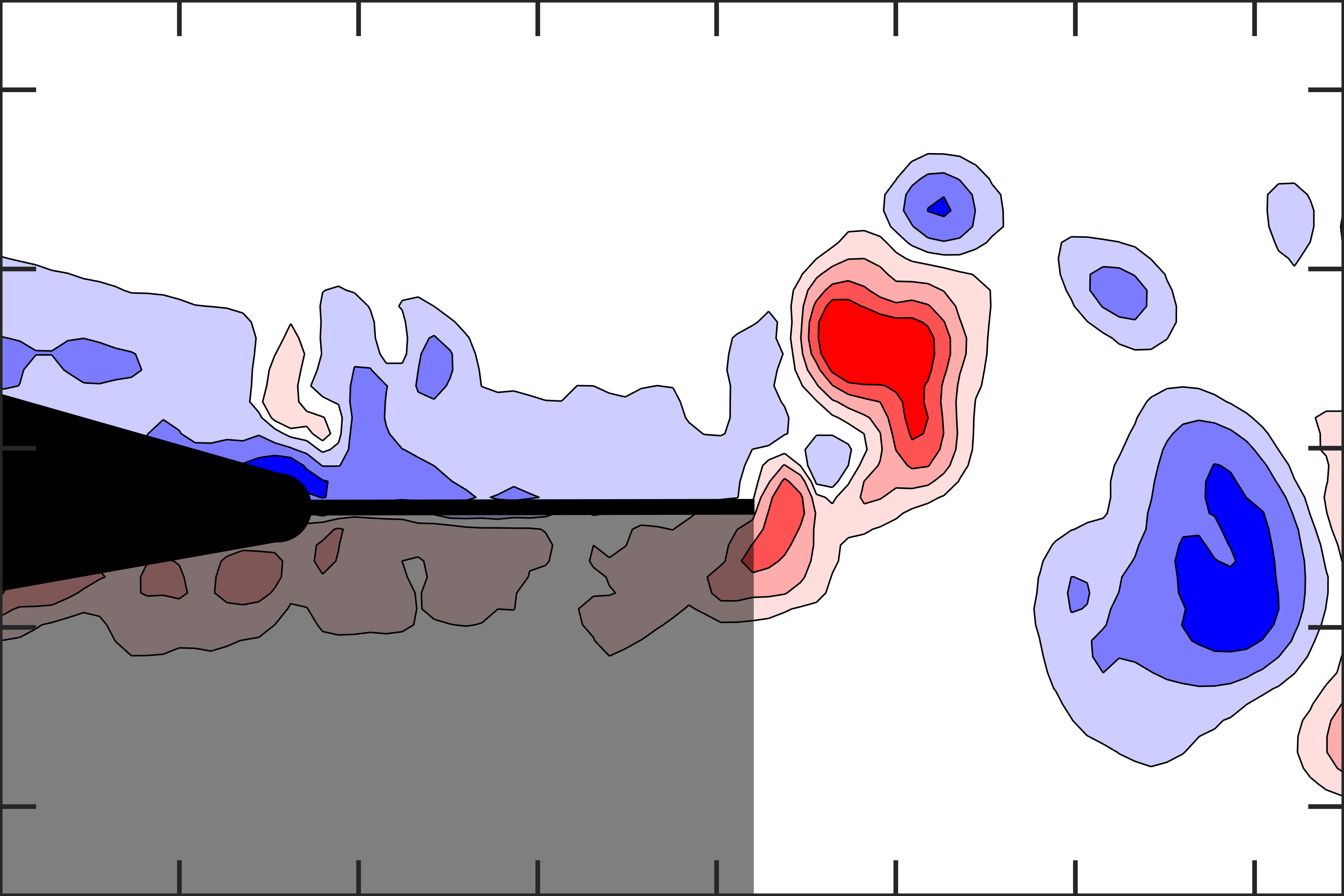

Supplement: Supplementary file 1 [file biomimetics-04-00067-s001.zip › Brooks_Green_Supplemental_Materials/Figures/TEVel_St0p37_T03p03_C05p00_p00mm_pActual22_pRaw18.png]

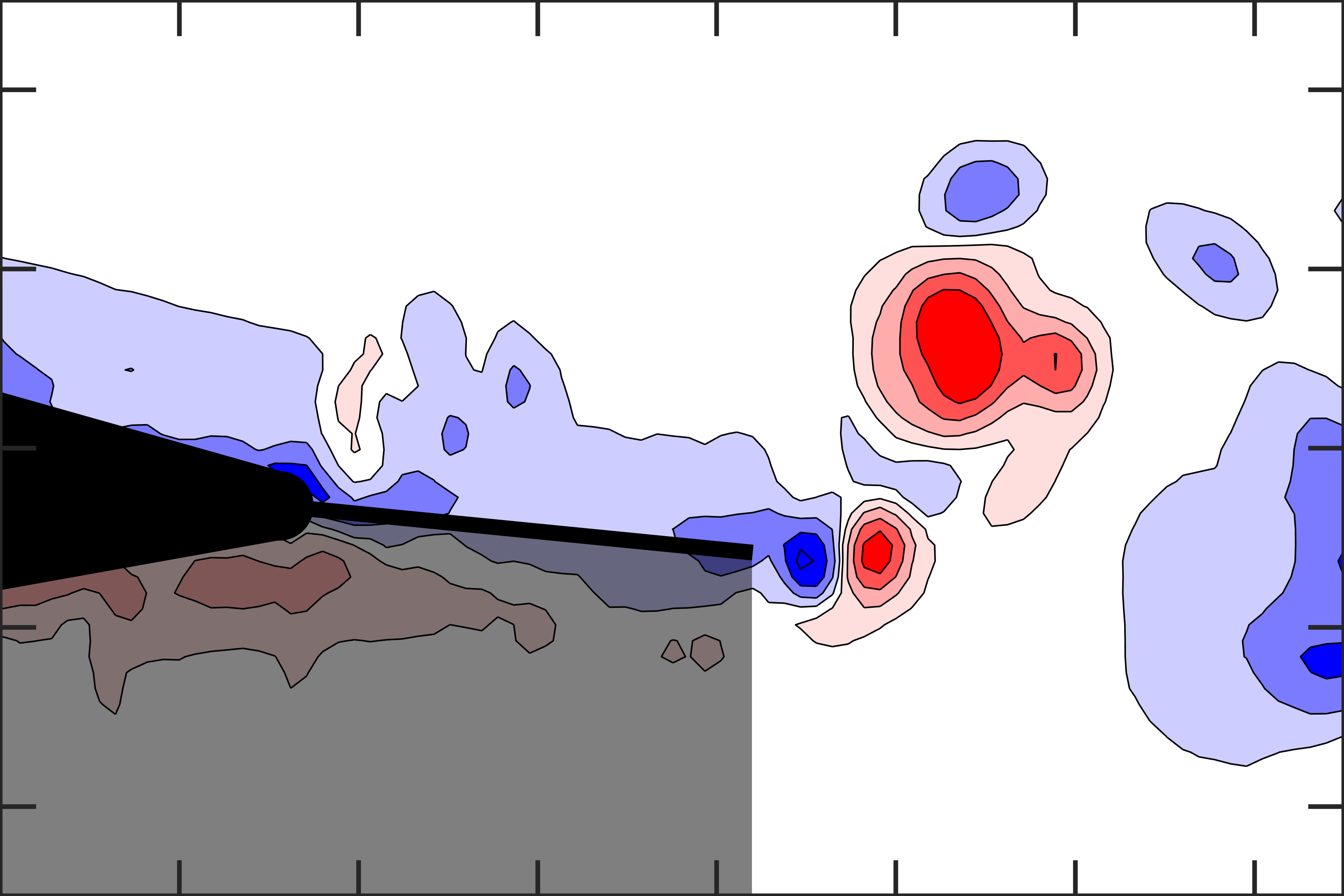

Supplement: Supplementary file 1 [file biomimetics-04-00067-s001.zip › Brooks_Green_Supplemental_Materials/Figures/TEVel_St0p37_T03p03_C05p00_p00mm_pActual26_pRaw22.png]

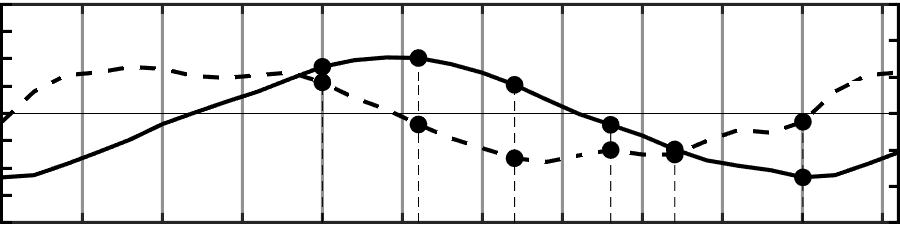

Supplement: Supplementary file 1 [file biomimetics-04-00067-s001.zip › Brooks_Green_Supplemental_Materials/Figures/TEVel_St0p37_T03p03_C05p00_p00mm_Velocity.png]

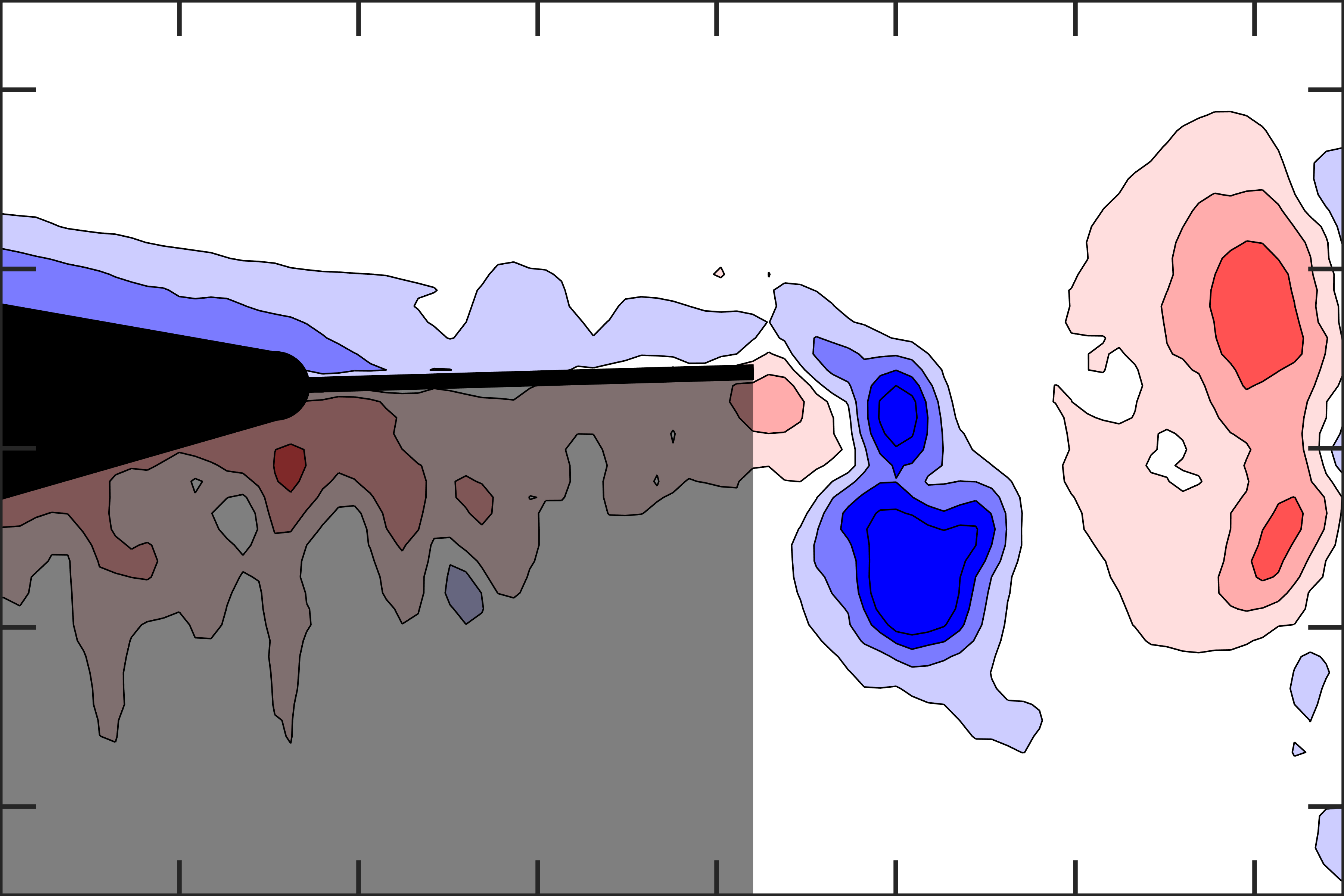

Supplement: Supplementary file 1 [file biomimetics-04-00067-s001.zip › Brooks_Green_Supplemental_Materials/Figures/TEVel_St0p37_T03p64_C00p00_p00mm_pActual11_pRaw05.png]

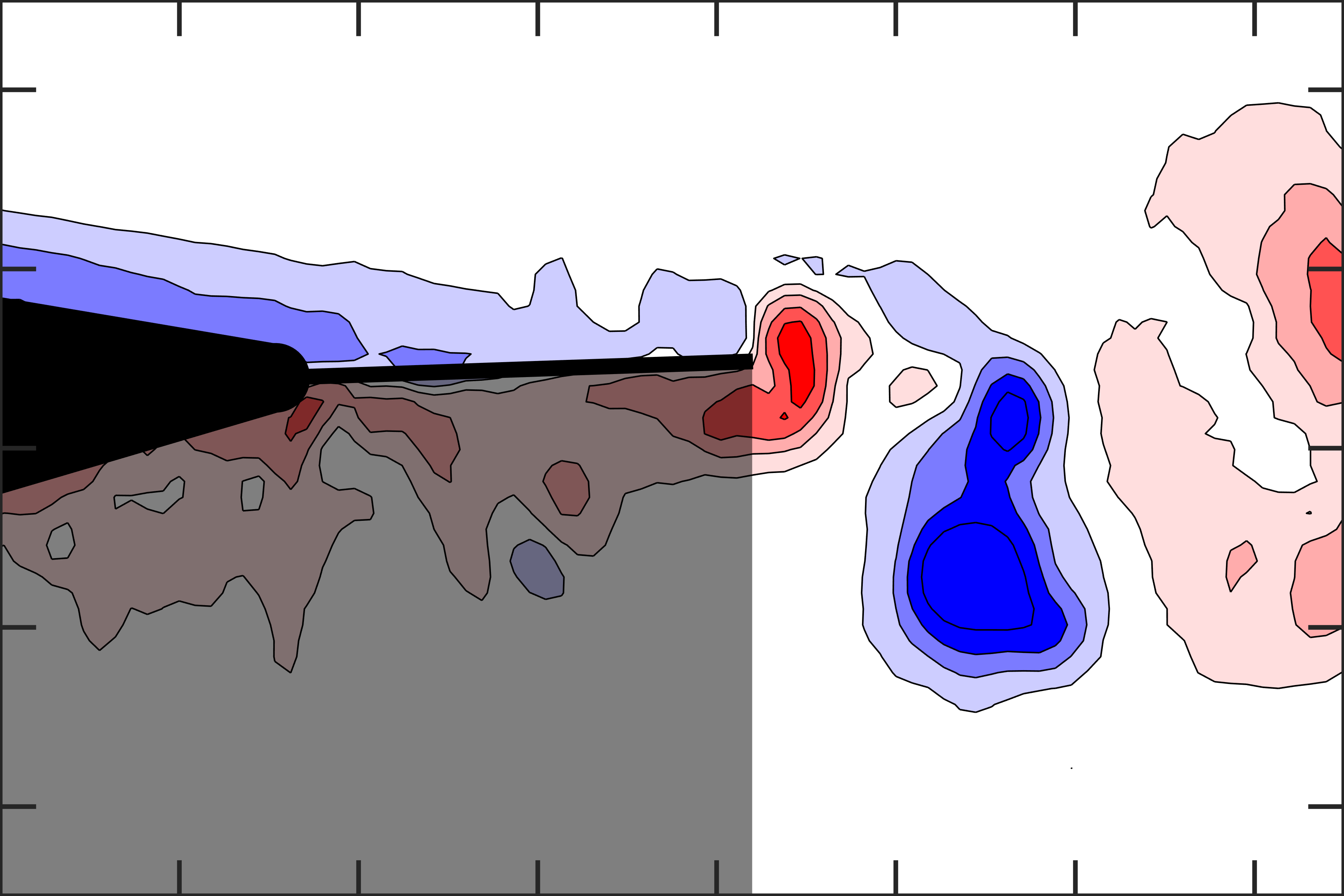

Supplement: Supplementary file 1 [file biomimetics-04-00067-s001.zip › Brooks_Green_Supplemental_Materials/Figures/TEVel_St0p37_T03p64_C00p00_p00mm_pActual14_pRaw08.png]

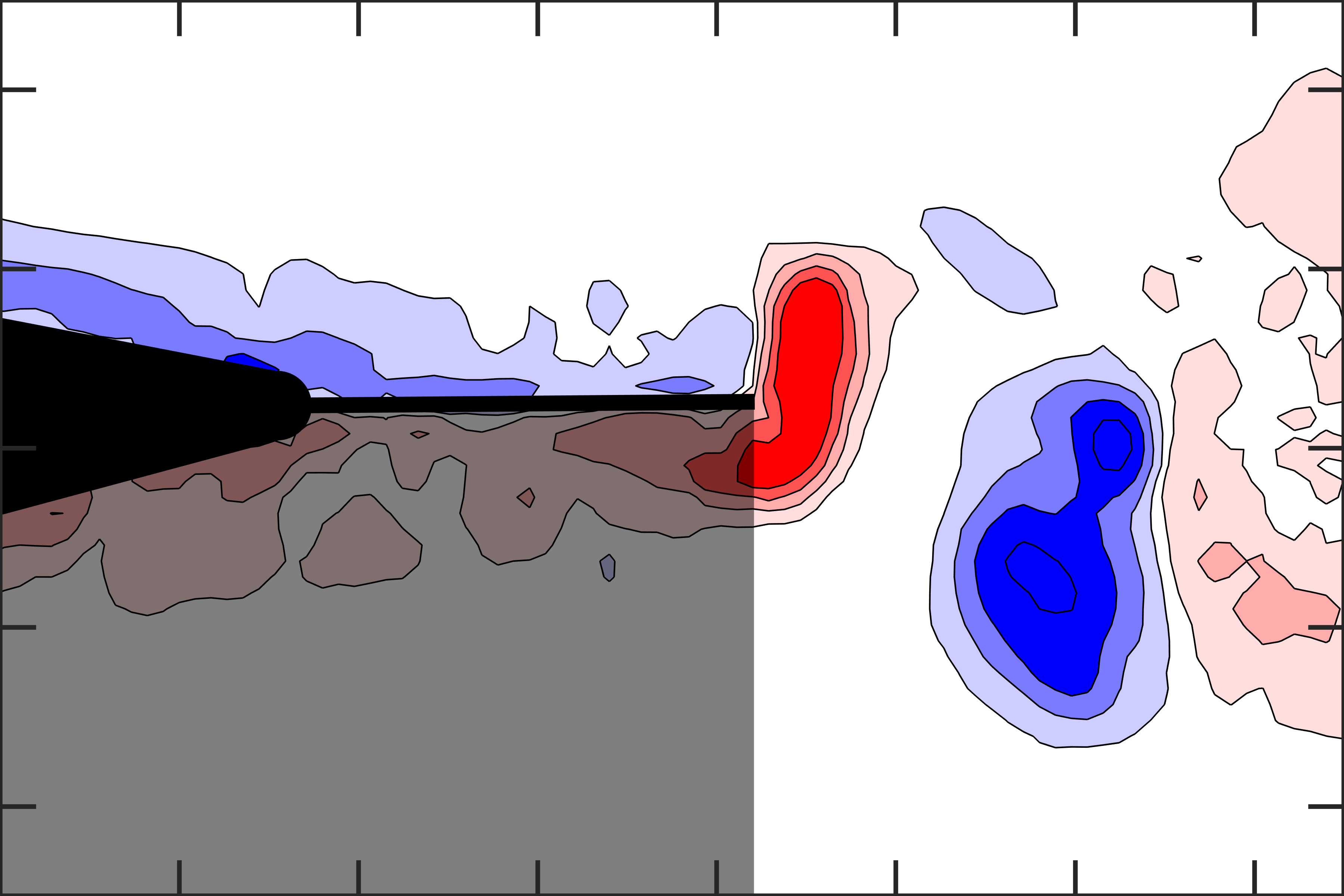

Supplement: Supplementary file 1 [file biomimetics-04-00067-s001.zip › Brooks_Green_Supplemental_Materials/Figures/TEVel_St0p37_T03p64_C00p00_p00mm_pActual17_pRaw11.png]

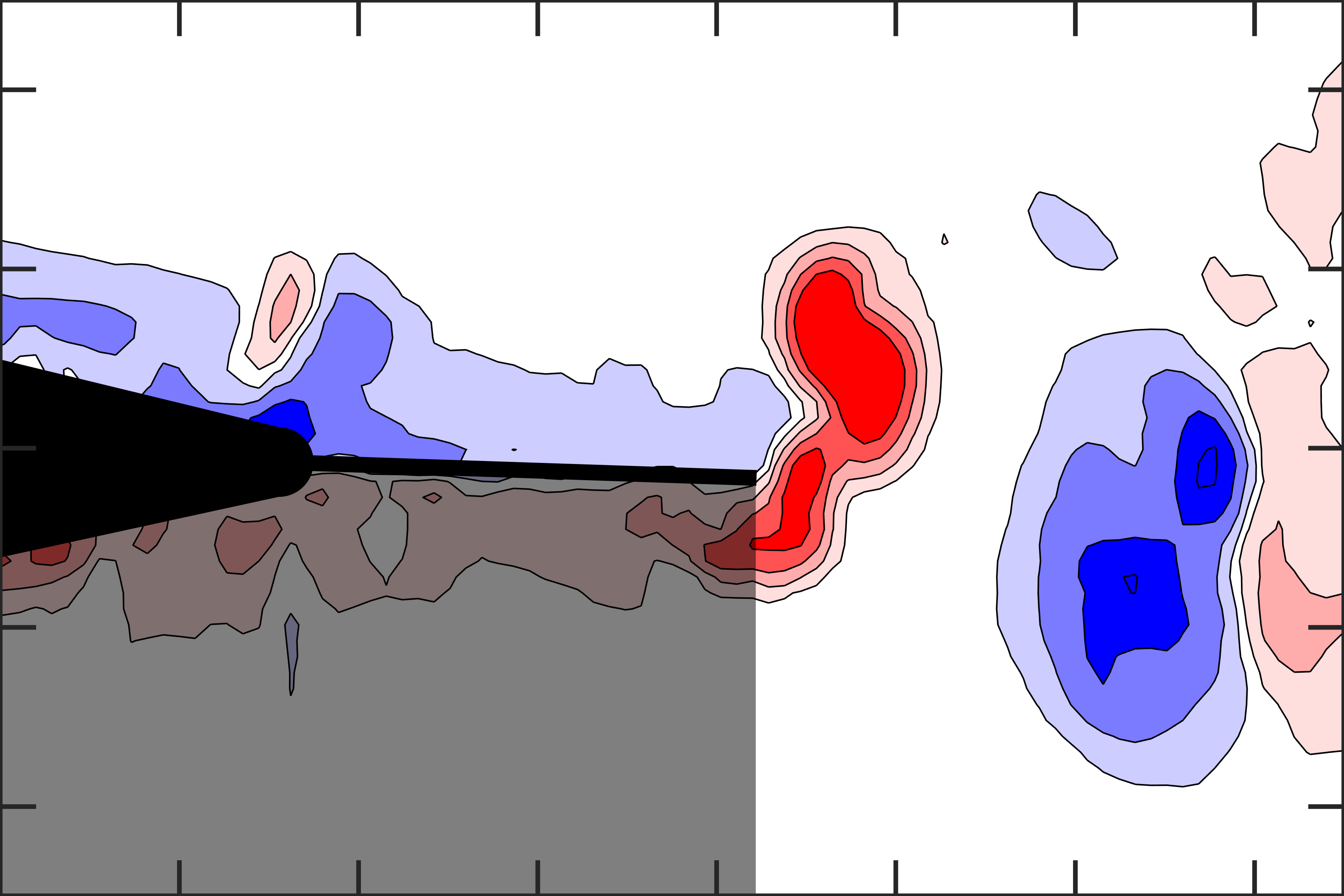

Supplement: Supplementary file 1 [file biomimetics-04-00067-s001.zip › Brooks_Green_Supplemental_Materials/Figures/TEVel_St0p37_T03p64_C00p00_p00mm_pActual20_pRaw14.png]

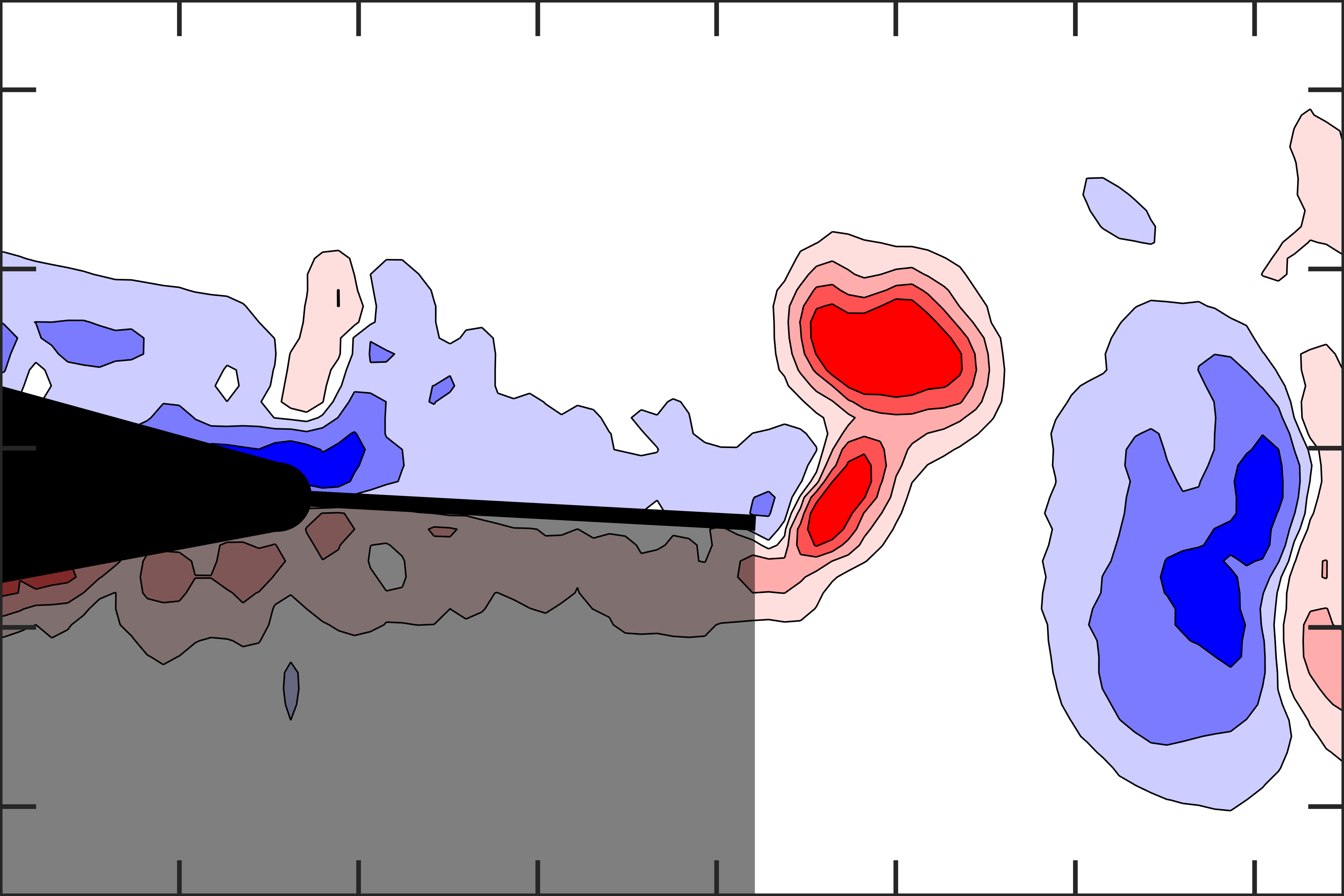

Supplement: Supplementary file 1 [file biomimetics-04-00067-s001.zip › Brooks_Green_Supplemental_Materials/Figures/TEVel_St0p37_T03p64_C00p00_p00mm_pActual22_pRaw16.png]

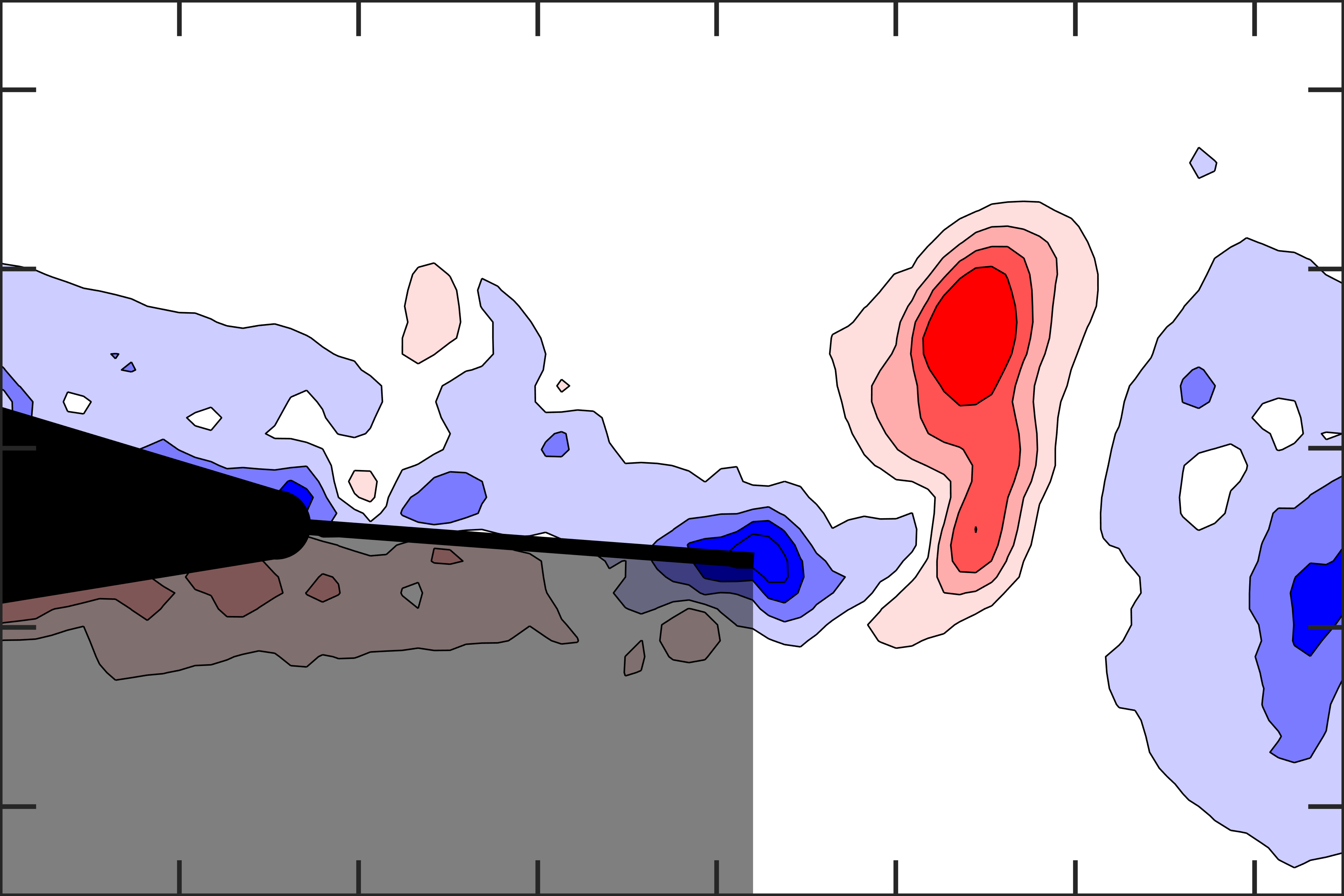

Supplement: Supplementary file 1 [file biomimetics-04-00067-s001.zip › Brooks_Green_Supplemental_Materials/Figures/TEVel_St0p37_T03p64_C00p00_p00mm_pActual26_pRaw20.png]

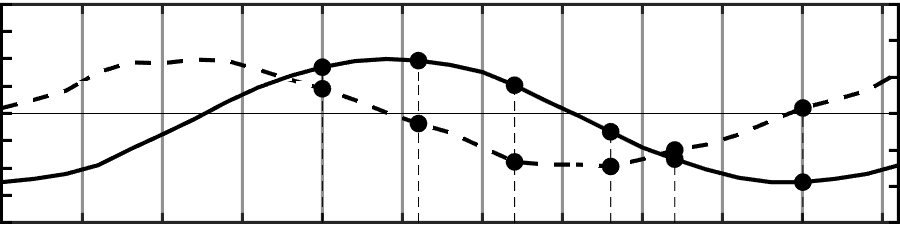

Supplement: Supplementary file 1 [file biomimetics-04-00067-s001.zip › Brooks_Green_Supplemental_Materials/Figures/TEVel_St0p37_T03p64_C00p00_p00mm_Velocity.png]

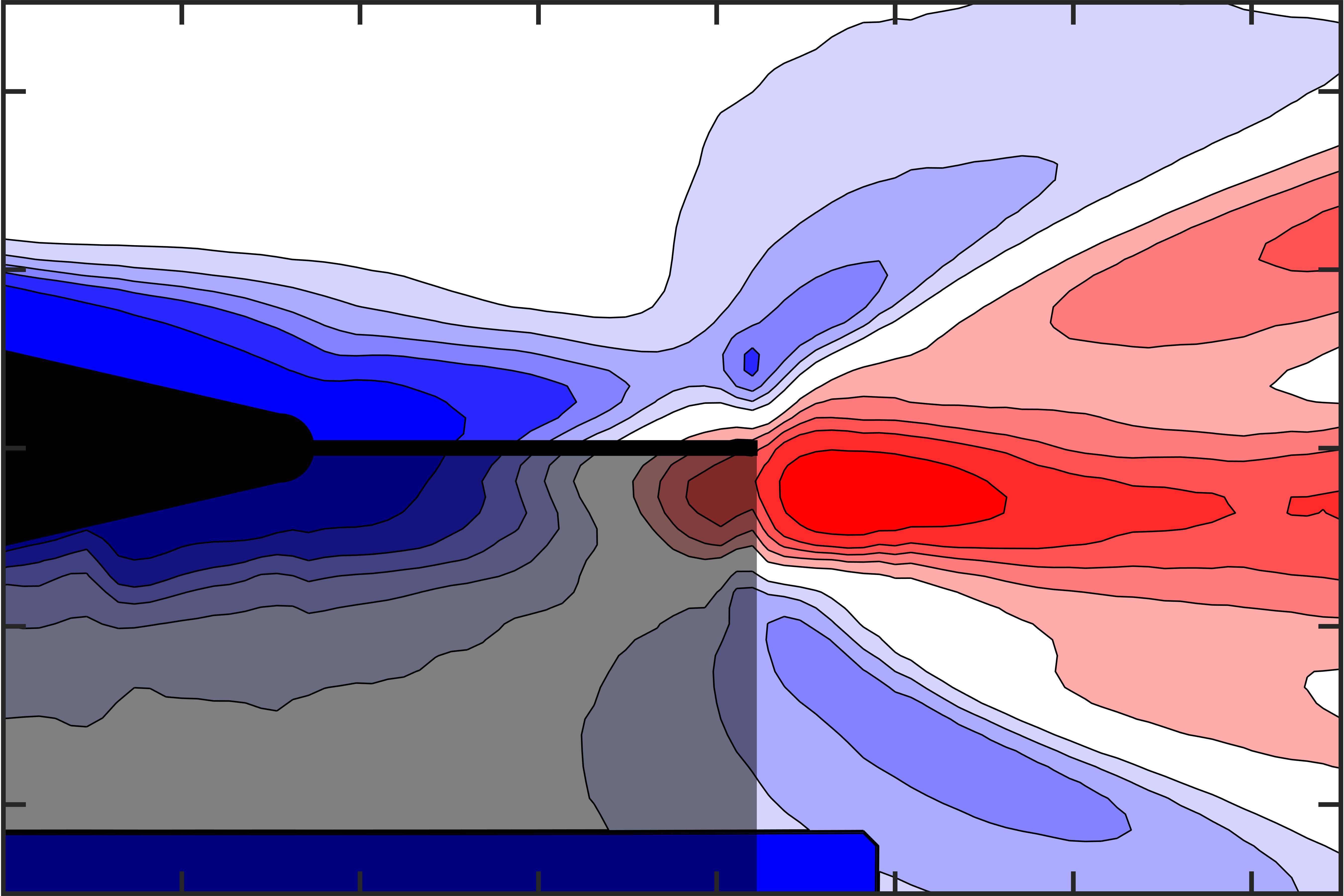

Supplement: Supplementary file 1 [file biomimetics-04-00067-s001.zip › Brooks_Green_Supplemental_Materials/Figures/WakeMom_St0p27_T00p00_C15p00_p00mm_uTA_NonDimensional.png]

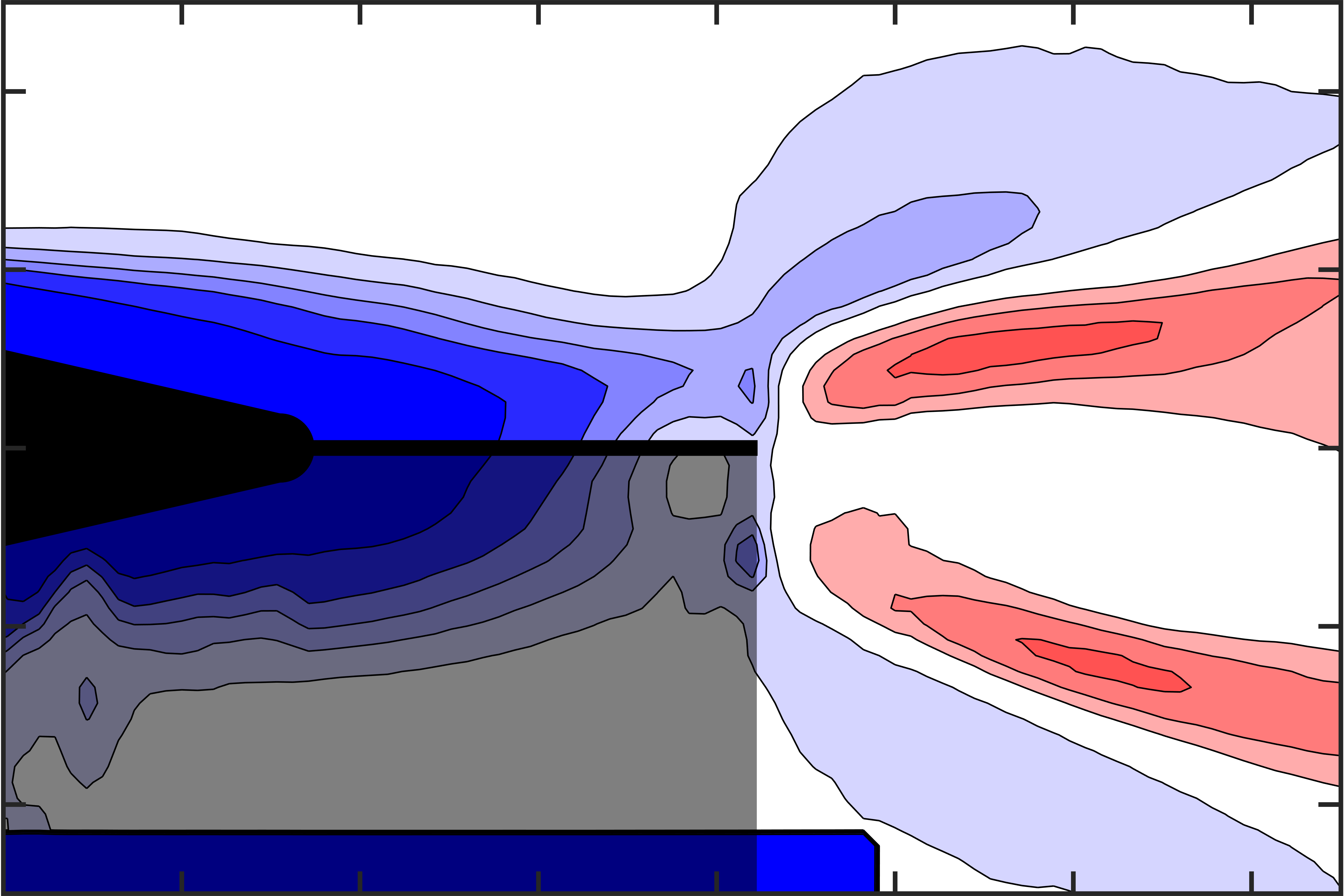

Supplement: Supplementary file 1 [file biomimetics-04-00067-s001.zip › Brooks_Green_Supplemental_Materials/Figures/WakeMom_St0p27_T01p99_C10p00_p00mm_uTA_NonDimensional.png]

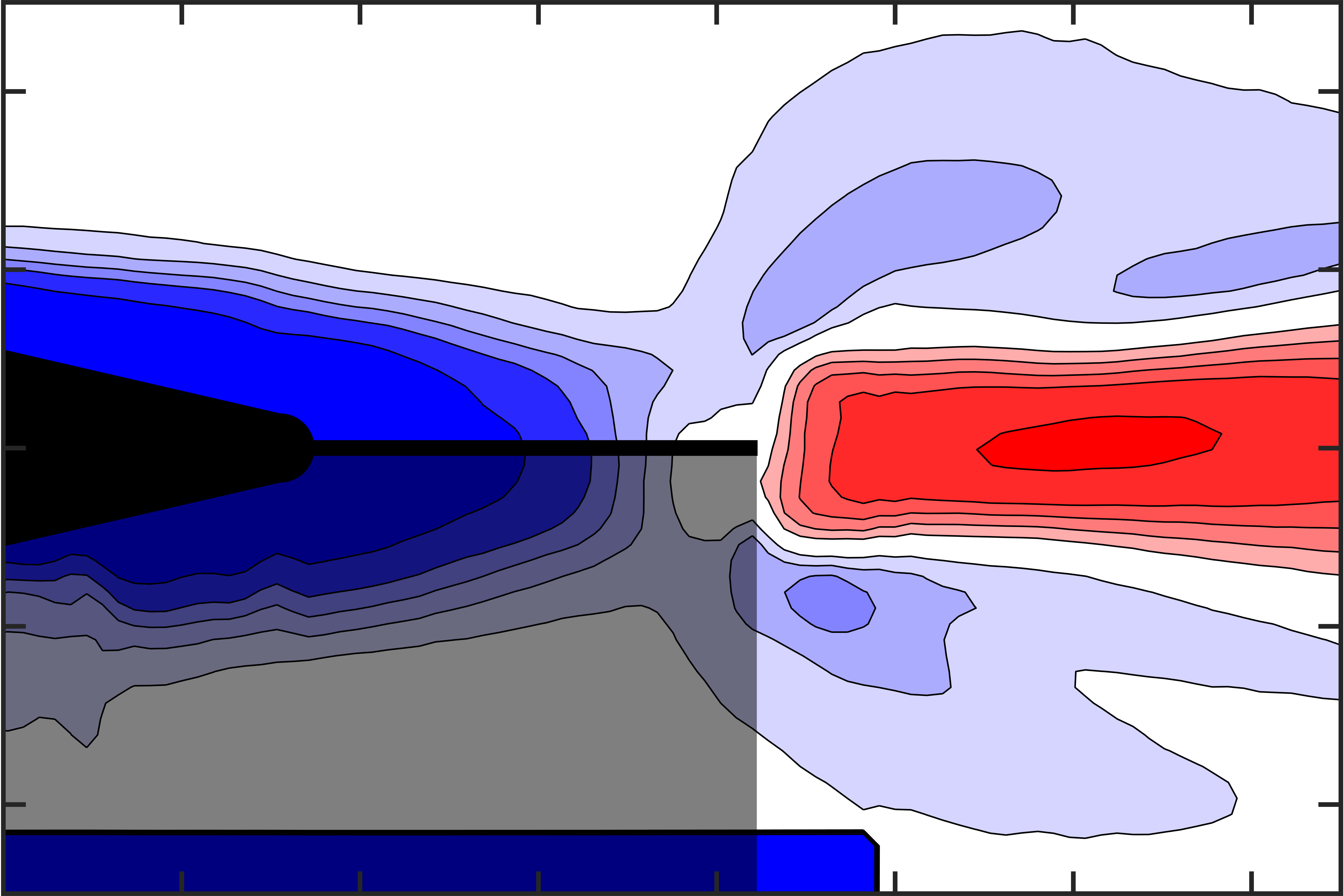

Supplement: Supplementary file 1 [file biomimetics-04-00067-s001.zip › Brooks_Green_Supplemental_Materials/Figures/WakeMom_St0p27_T03p03_C05p00_p00mm_uTA_NonDimensional.png]

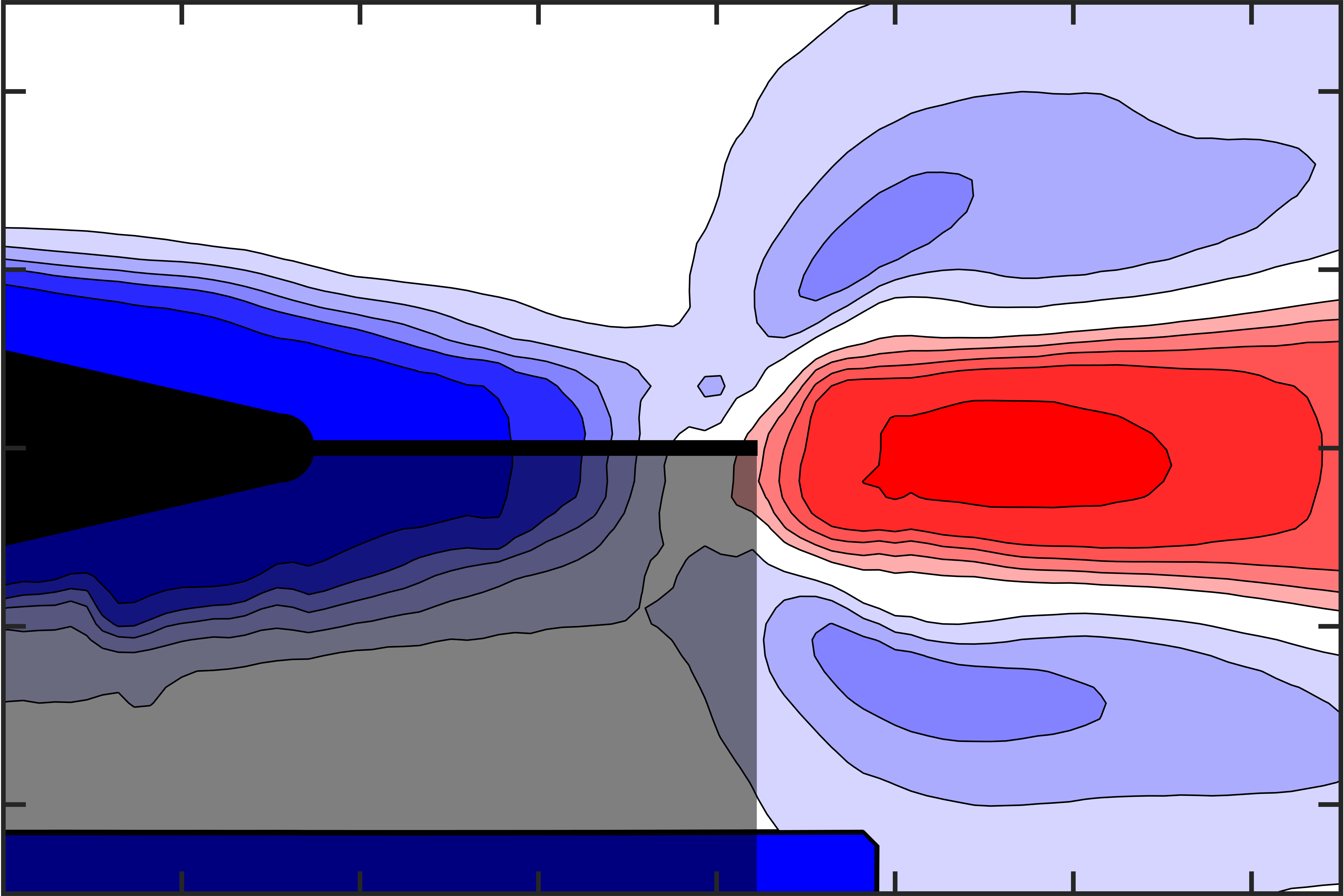

Supplement: Supplementary file 1 [file biomimetics-04-00067-s001.zip › Brooks_Green_Supplemental_Materials/Figures/WakeMom_St0p27_T03p64_C00p00_p00mm_uTA_NonDimensional.png]

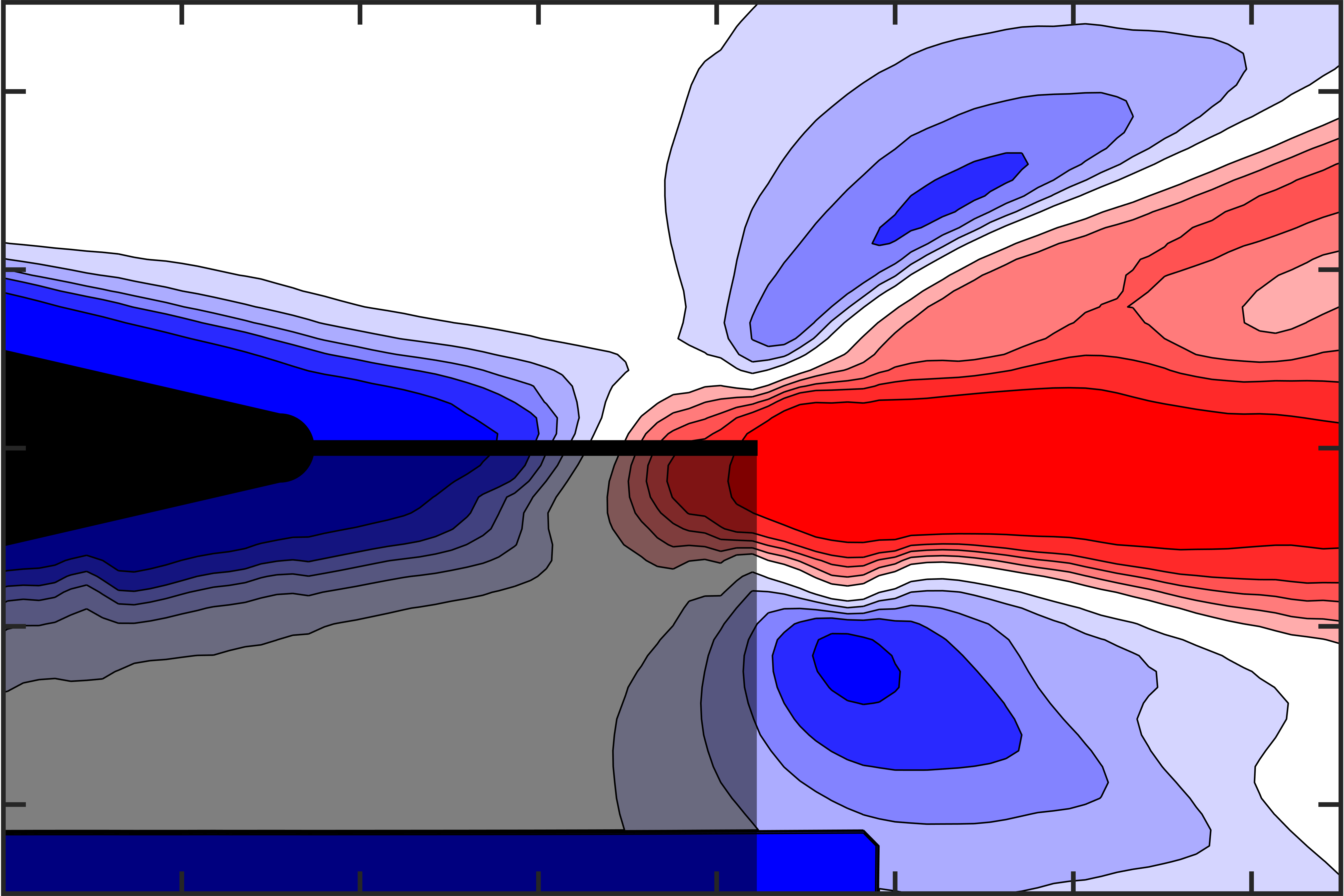

Supplement: Supplementary file 1 [file biomimetics-04-00067-s001.zip › Brooks_Green_Supplemental_Materials/Figures/WakeMom_St0p37_T00p00_C15p00_p00mm_uTA_NonDimensional.png]

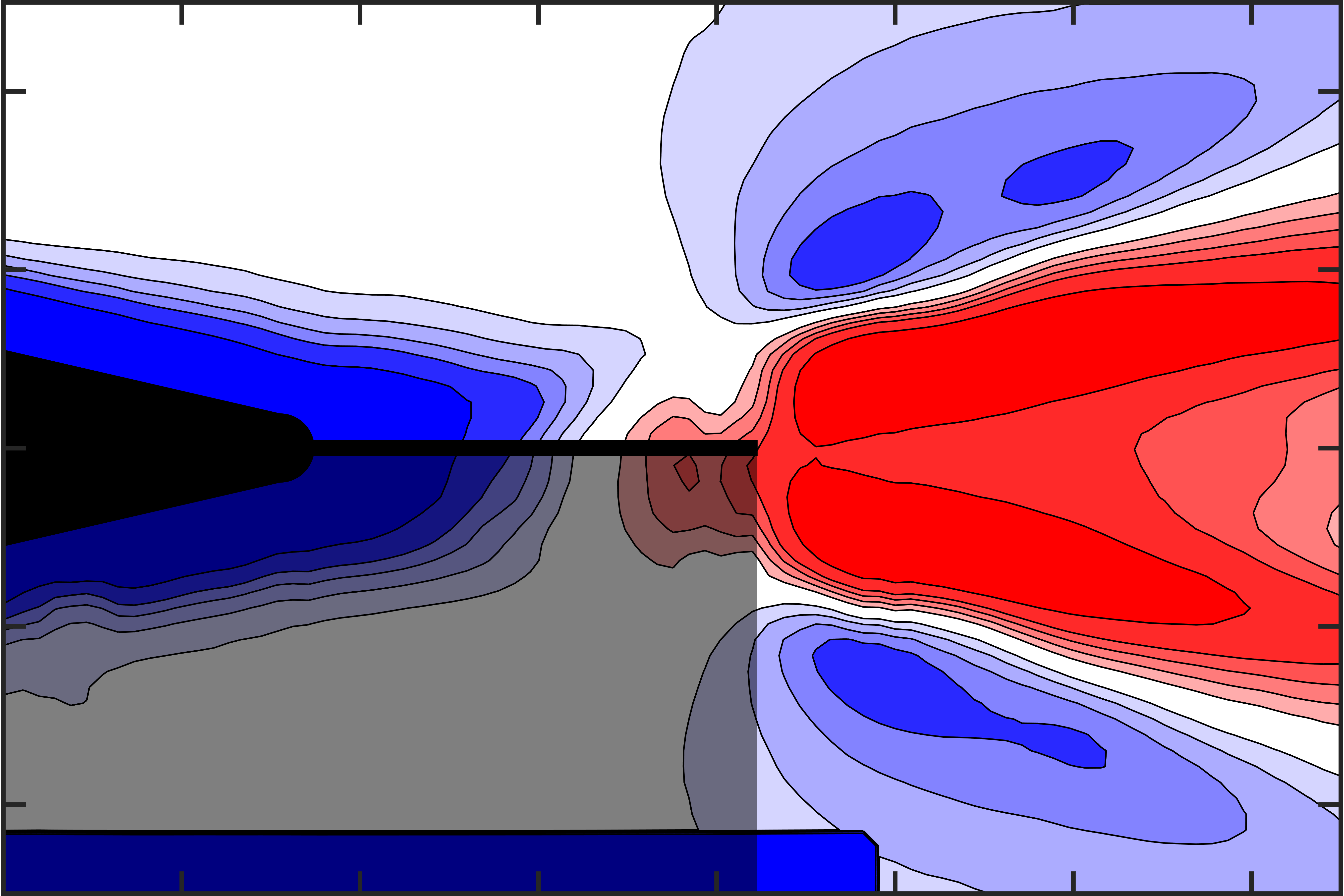

Supplement: Supplementary file 1 [file biomimetics-04-00067-s001.zip › Brooks_Green_Supplemental_Materials/Figures/WakeMom_St0p37_T01p99_C10p00_p00mm_uTA_NonDimensional.png]

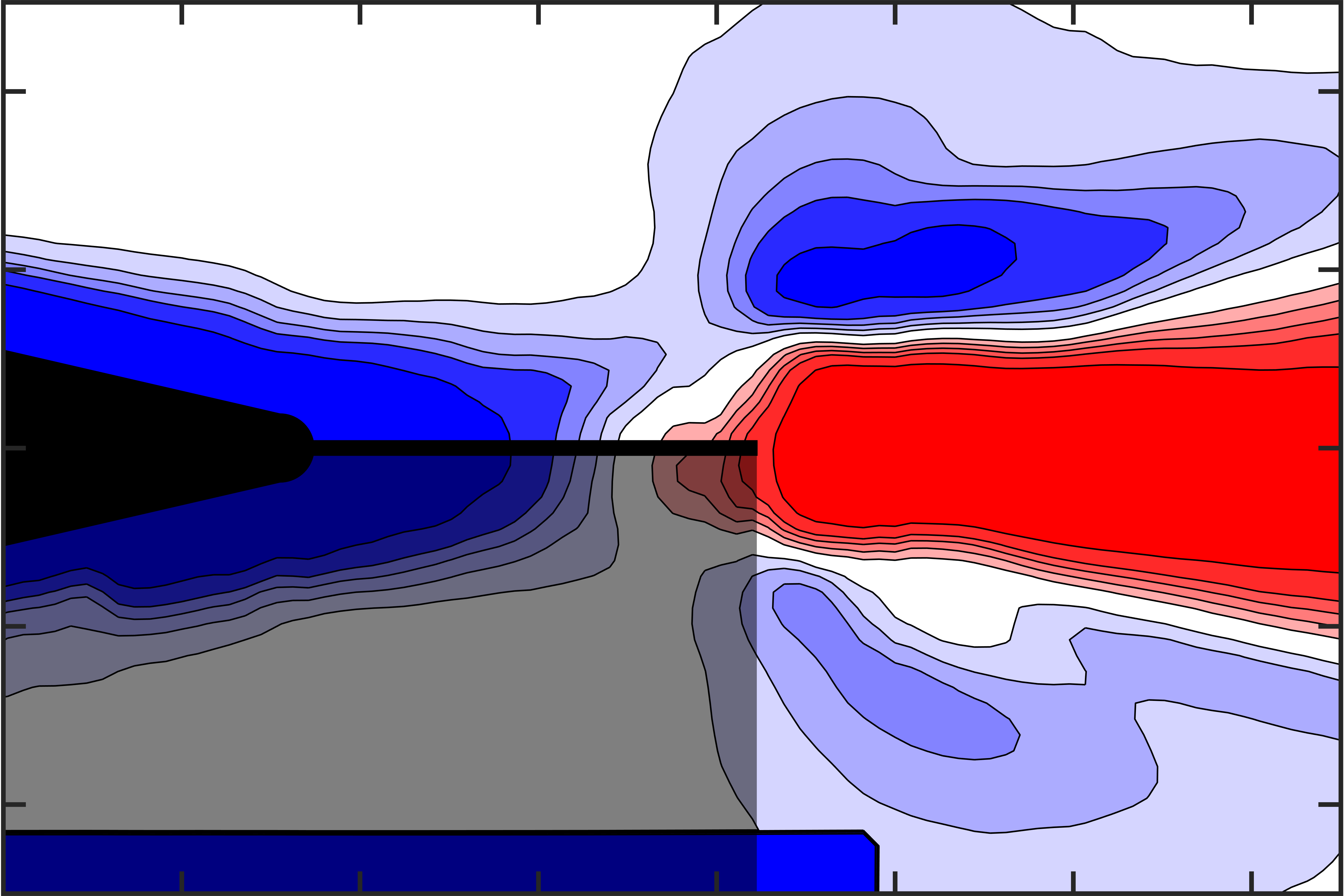

Supplement: Supplementary file 1 [file biomimetics-04-00067-s001.zip › Brooks_Green_Supplemental_Materials/Figures/WakeMom_St0p37_T03p03_C05p00_p00mm_uTA_NonDimensional.png]

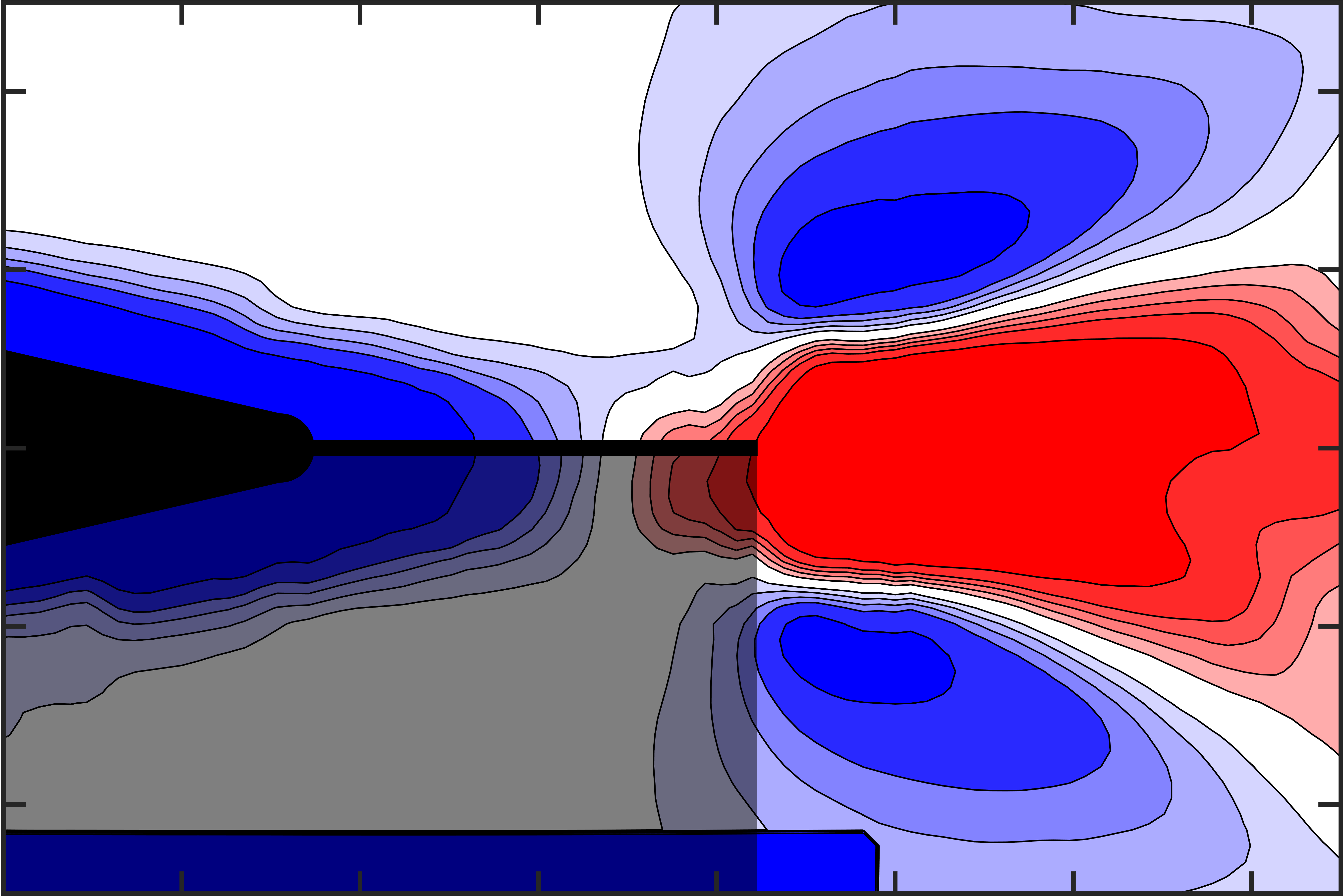

Supplement: Supplementary file 1 [file biomimetics-04-00067-s001.zip › Brooks_Green_Supplemental_Materials/Figures/WakeMom_St0p37_T03p64_C00p00_p00mm_uTA_NonDimensional.png]

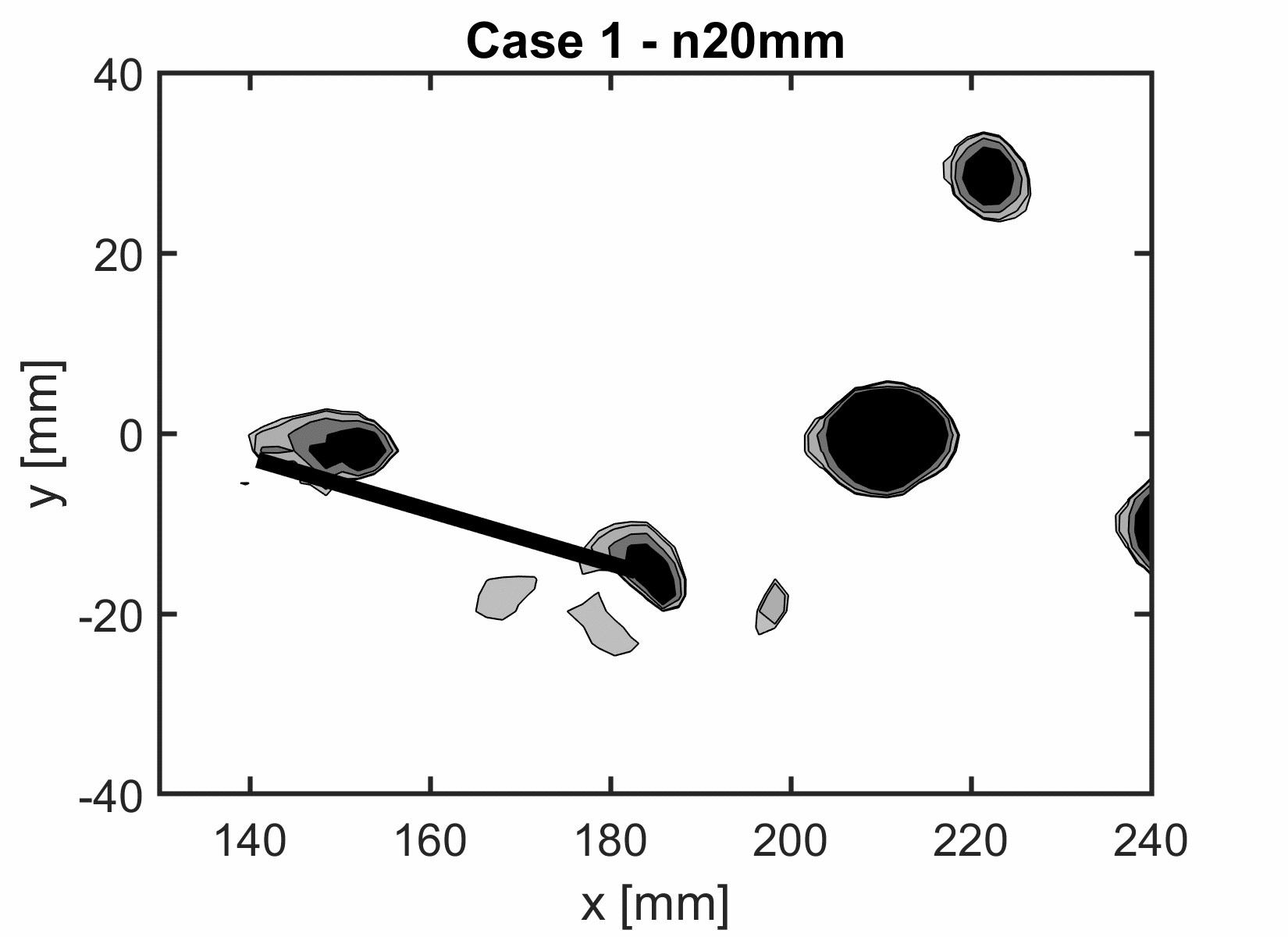

Supplement: Supplementary file 1 [file biomimetics-04-00067-s001.zip › Brooks_Green_Supplemental_Materials/GIFs/S1_Case1_n20mm.gif]

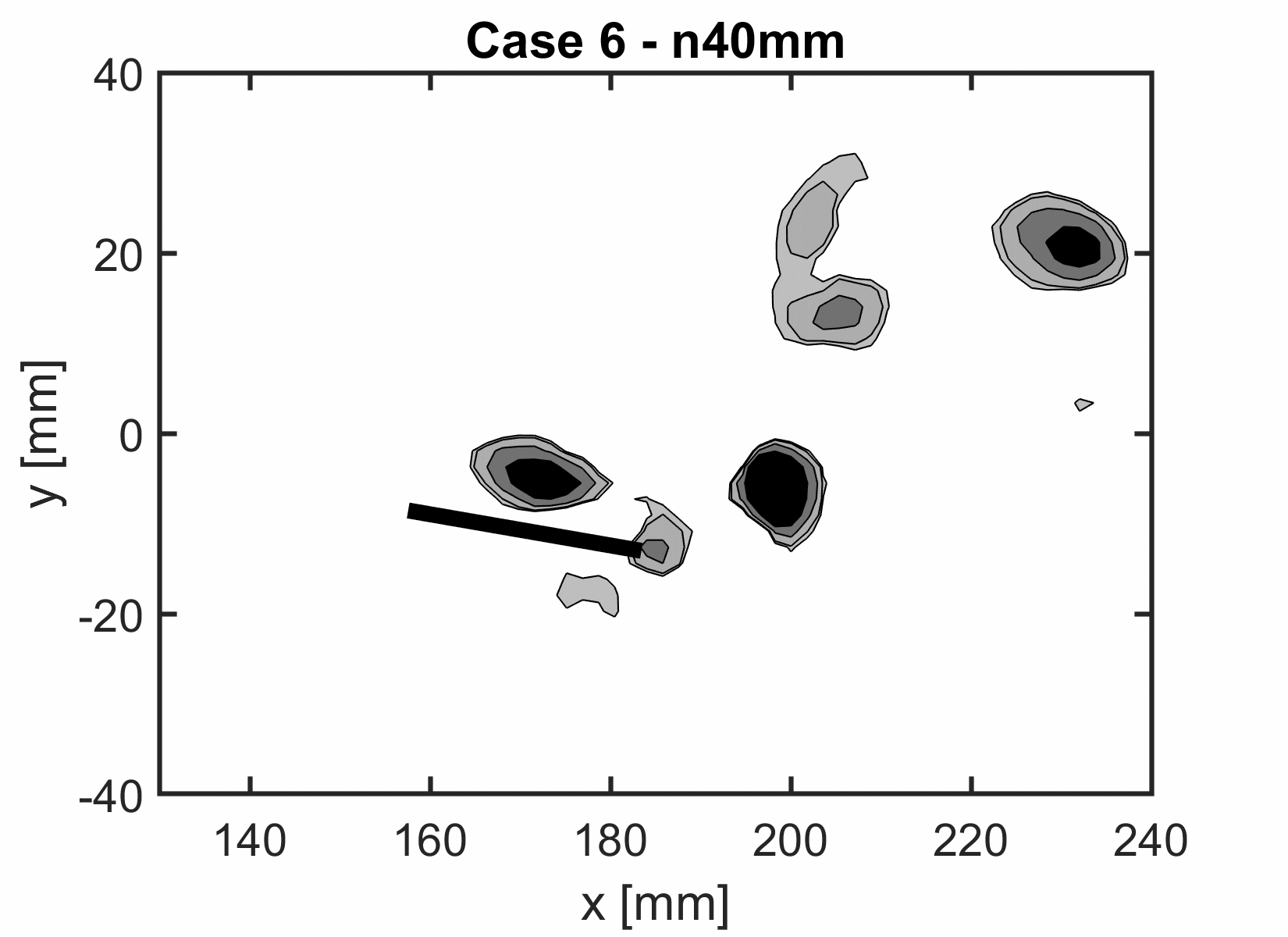

Supplement: Supplementary file 1 [file biomimetics-04-00067-s001.zip › Brooks_Green_Supplemental_Materials/GIFs/S10_Case6_n40mm.gif]

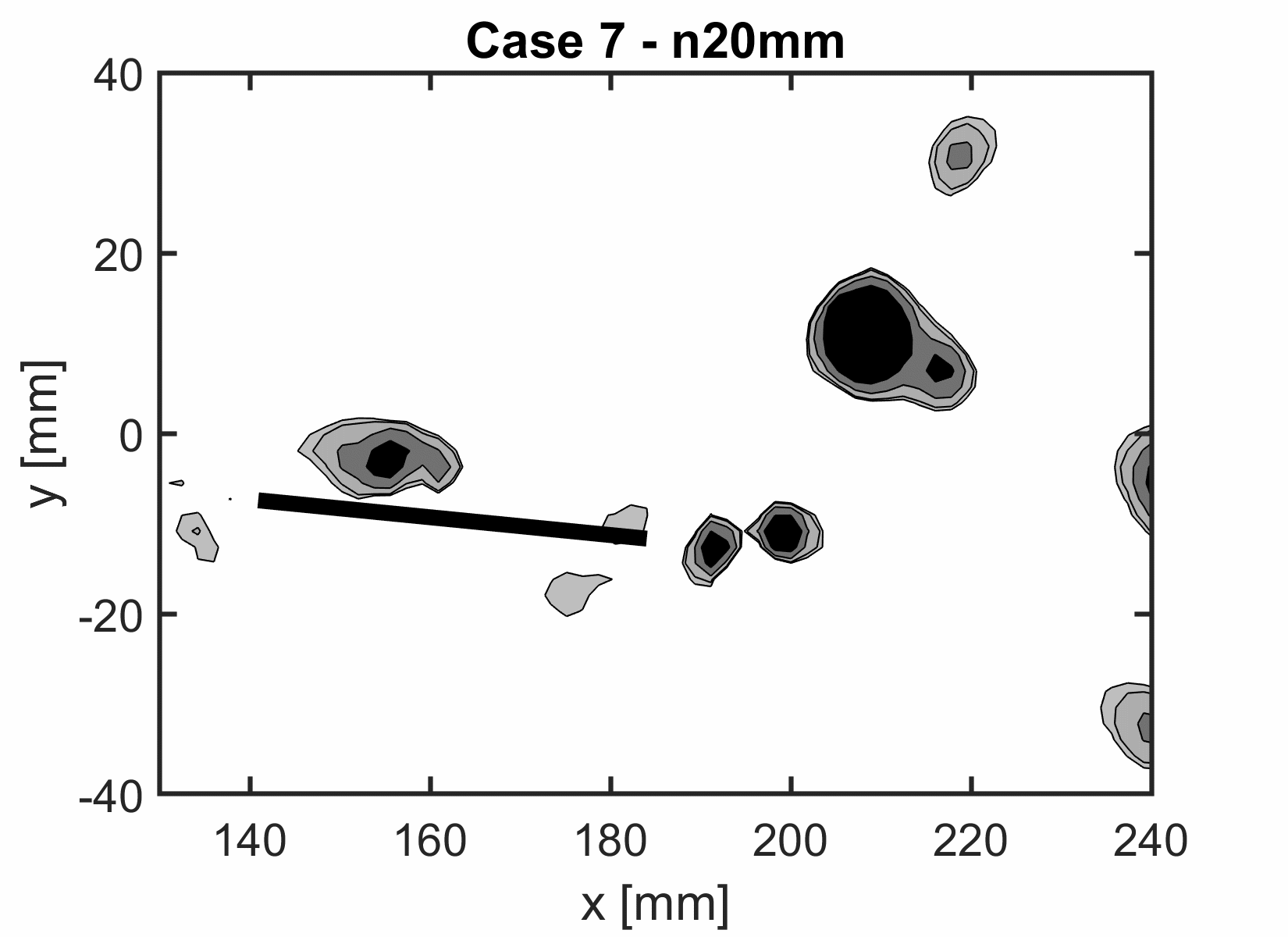

Supplement: Supplementary file 1 [file biomimetics-04-00067-s001.zip › Brooks_Green_Supplemental_Materials/GIFs/S11_Case7_n20mm.gif]

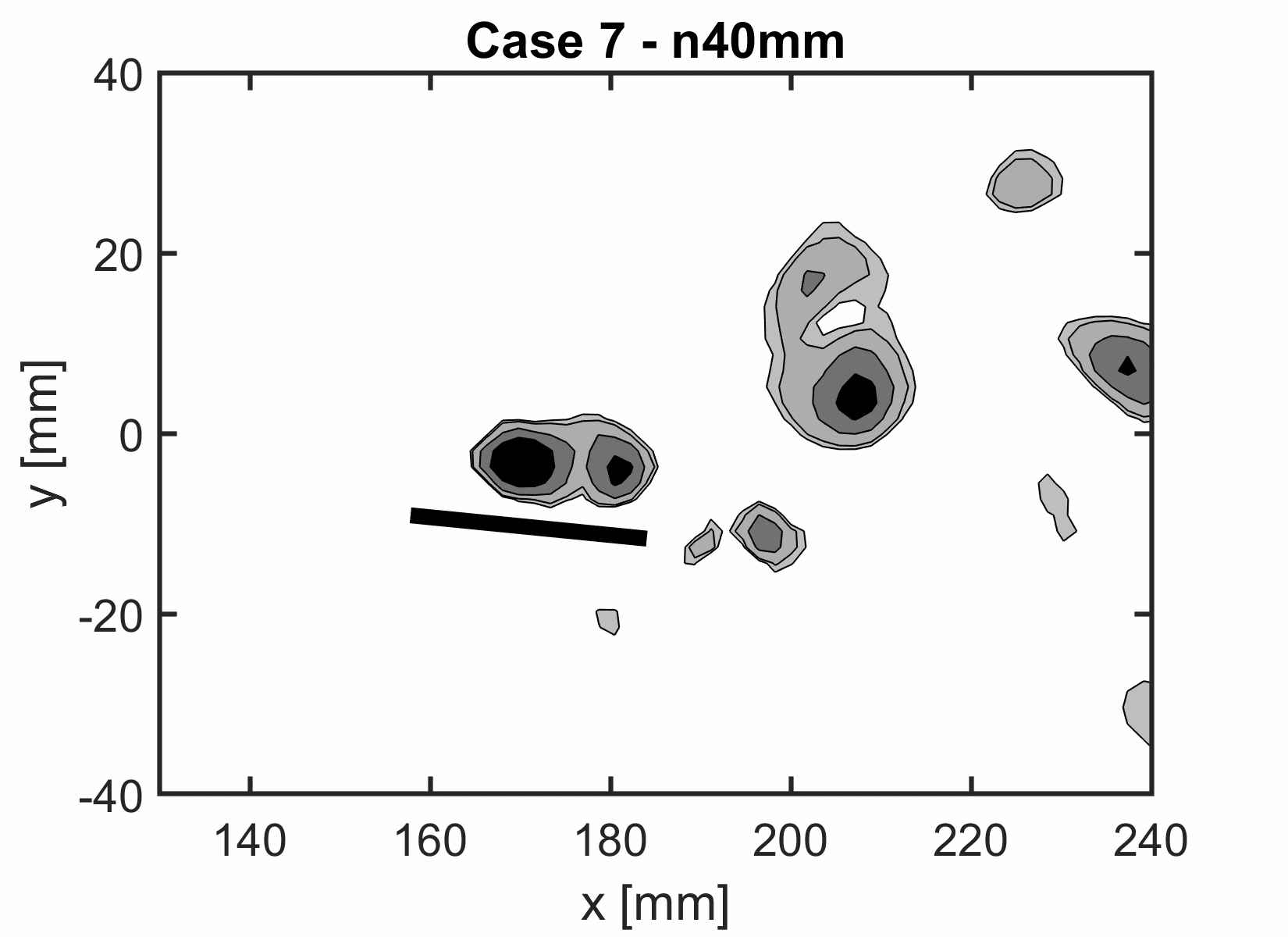

Supplement: Supplementary file 1 [file biomimetics-04-00067-s001.zip › Brooks_Green_Supplemental_Materials/GIFs/S12_Case7_n40mm.gif]

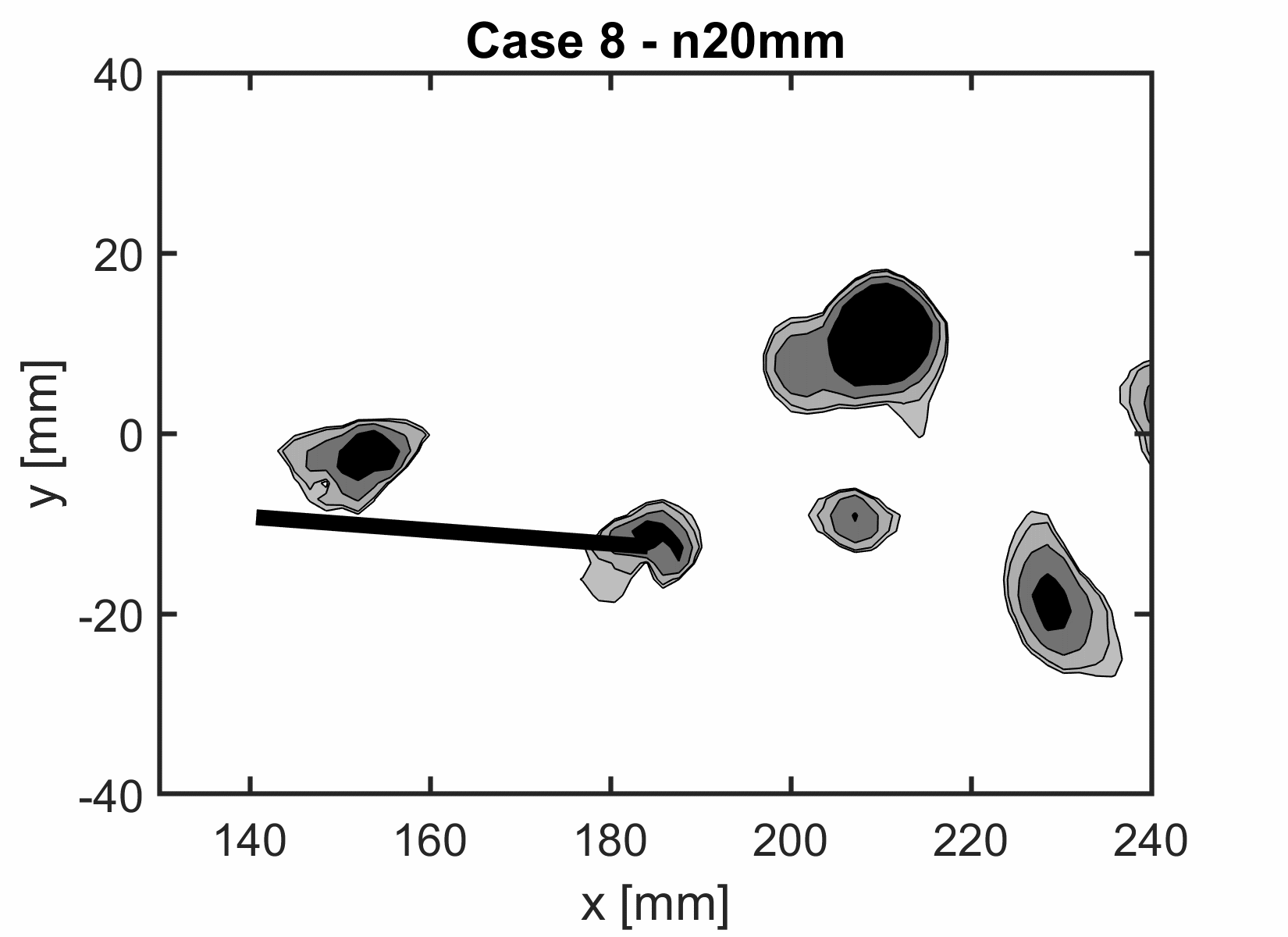

Supplement: Supplementary file 1 [file biomimetics-04-00067-s001.zip › Brooks_Green_Supplemental_Materials/GIFs/S13_Case8_n20mm.gif]

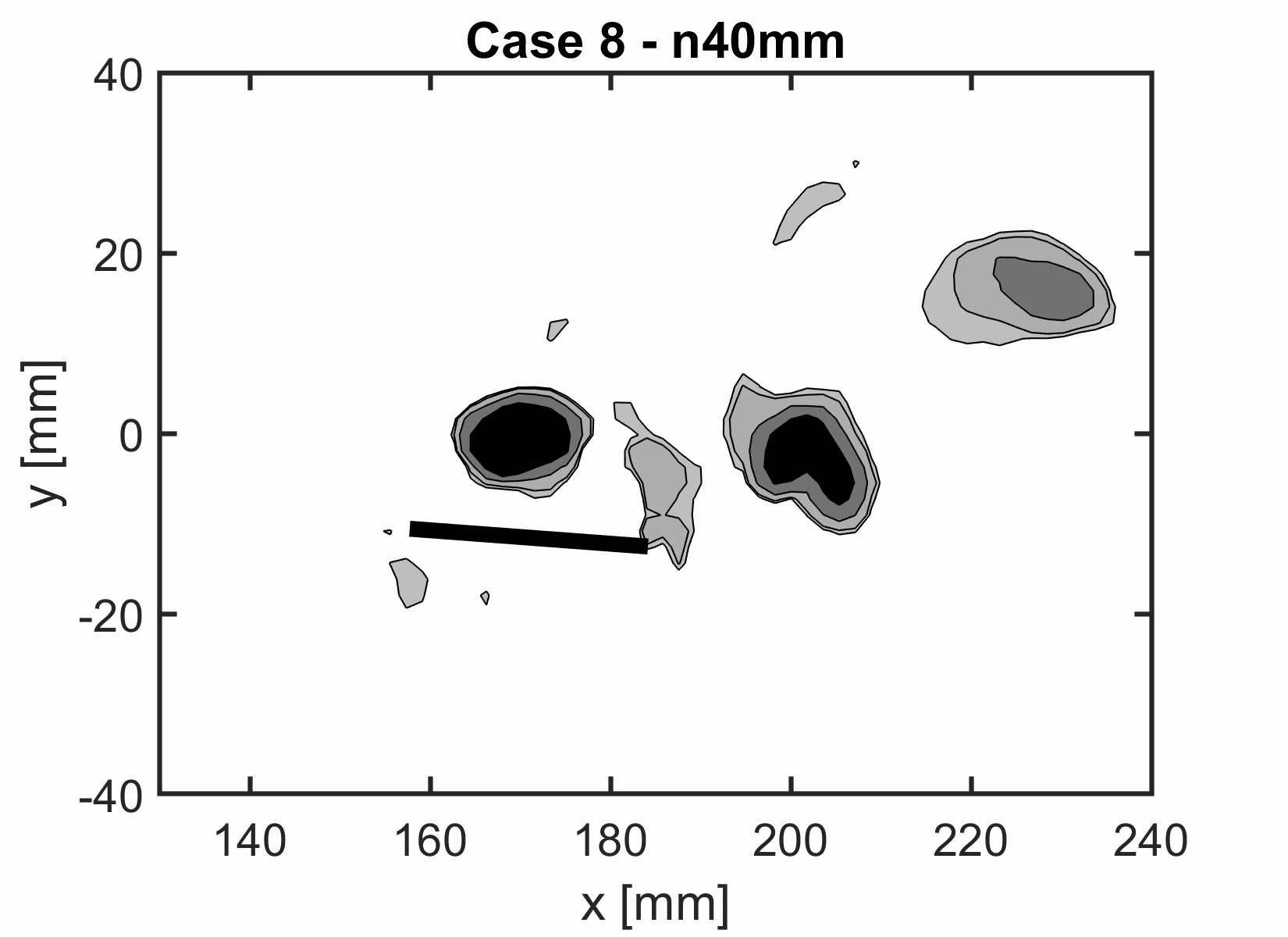

Supplement: Supplementary file 1 [file biomimetics-04-00067-s001.zip › Brooks_Green_Supplemental_Materials/GIFs/S14_Case8_n40mm.gif]

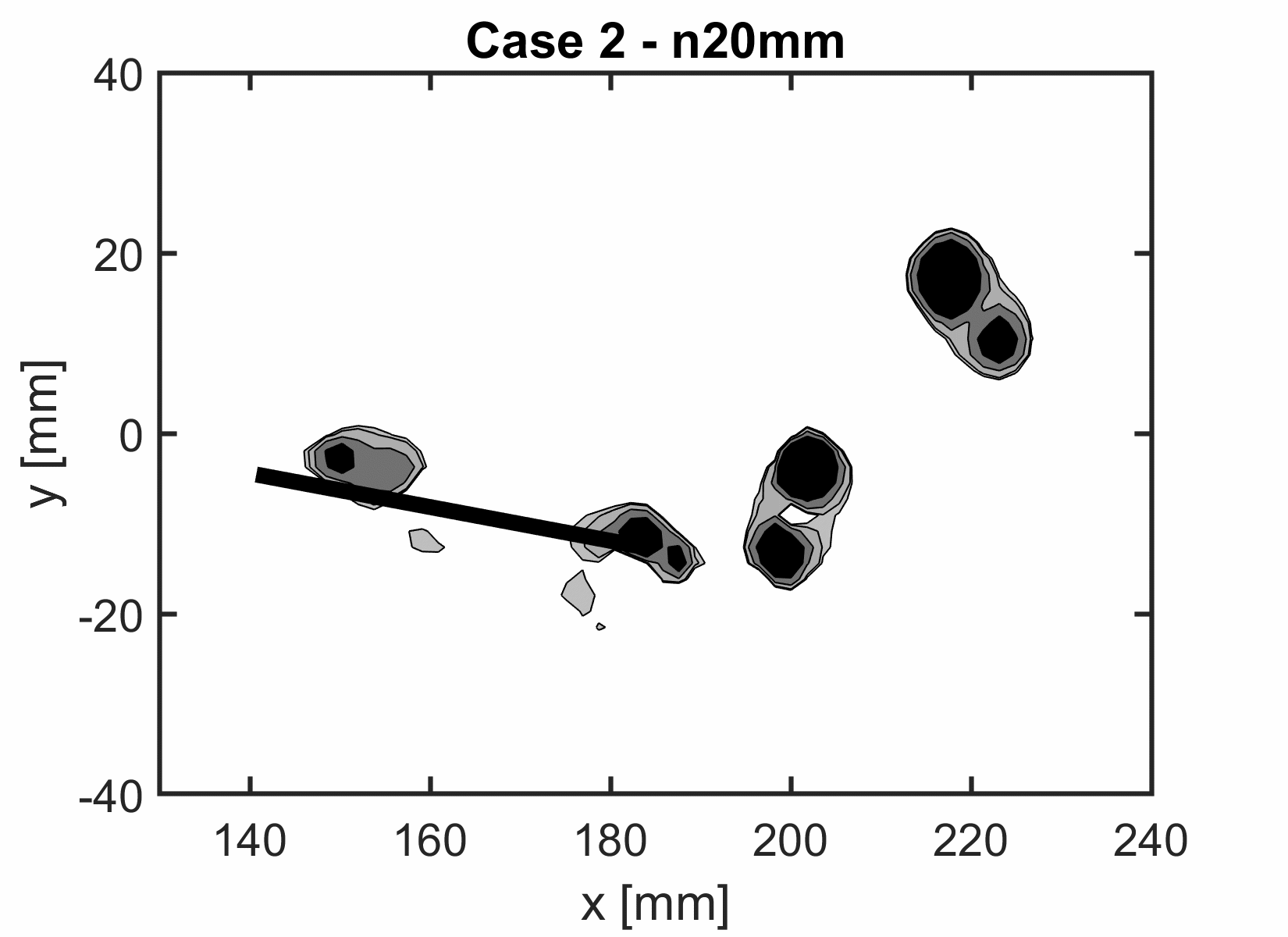

Supplement: Supplementary file 1 [file biomimetics-04-00067-s001.zip › Brooks_Green_Supplemental_Materials/GIFs/S2_Case2_n20mm.gif]

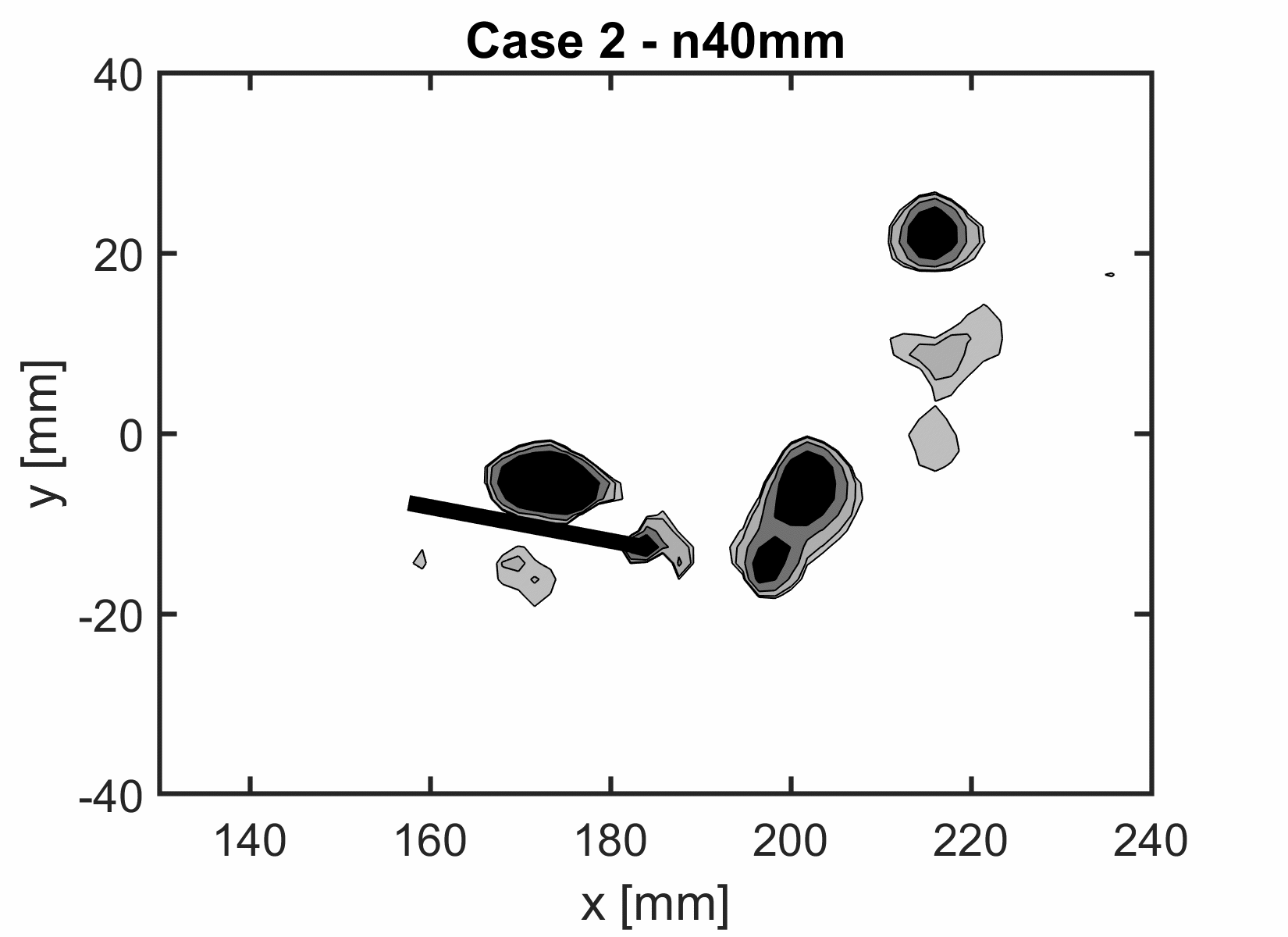

Supplement: Supplementary file 1 [file biomimetics-04-00067-s001.zip › Brooks_Green_Supplemental_Materials/GIFs/S3_Case2_n40mm.gif]

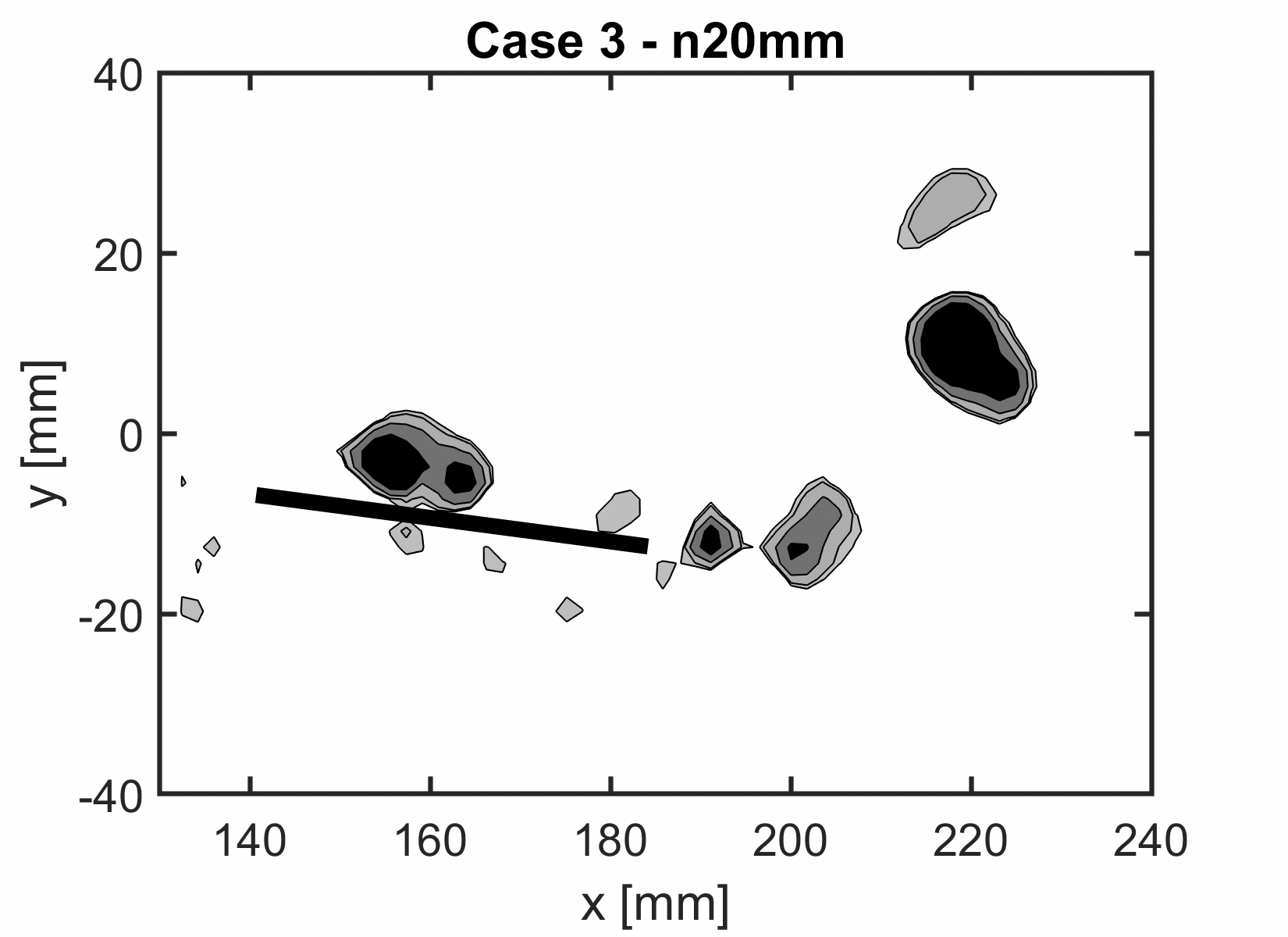

Supplement: Supplementary file 1 [file biomimetics-04-00067-s001.zip › Brooks_Green_Supplemental_Materials/GIFs/S4_Case3_n20mm.gif]

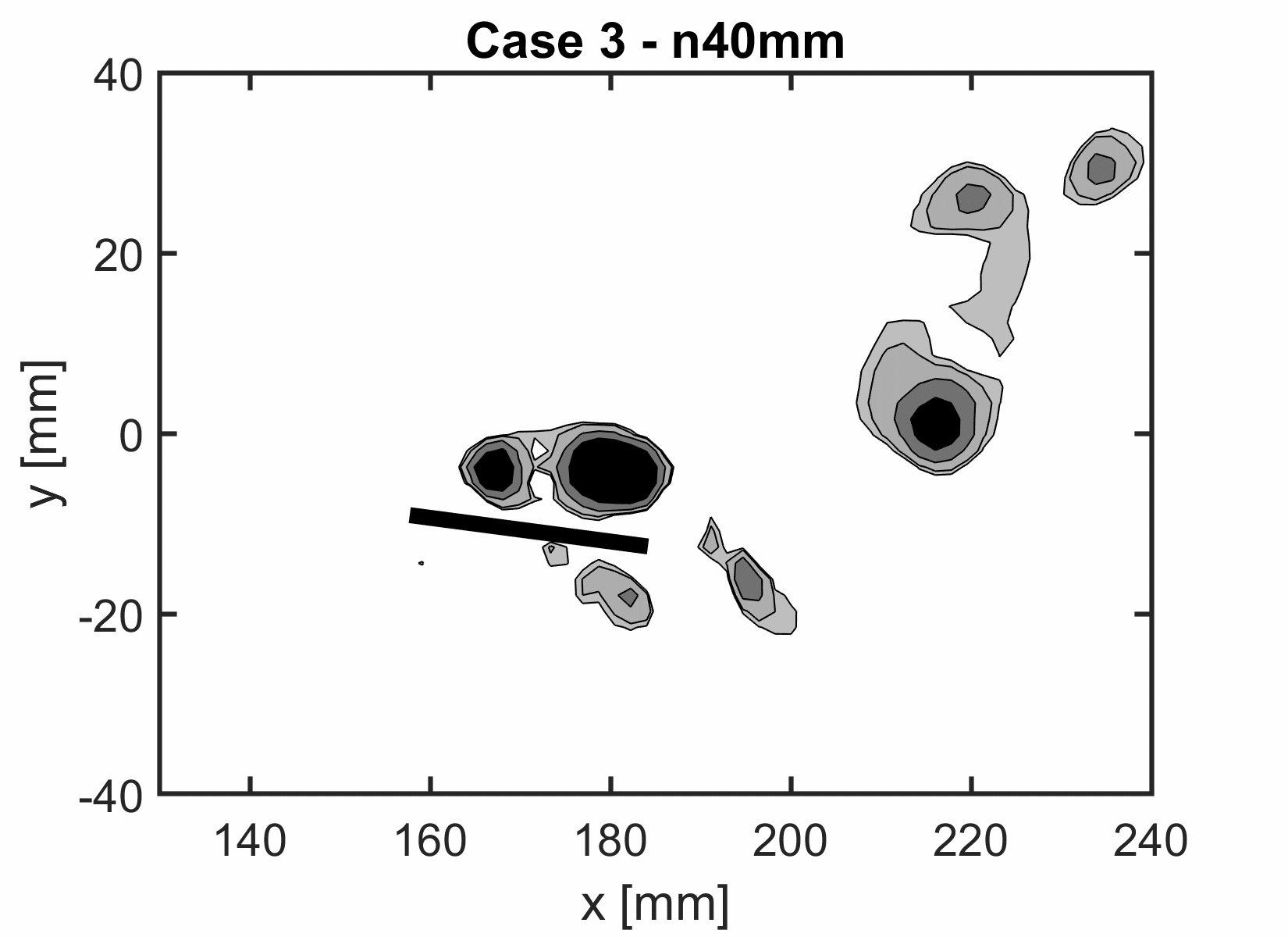

Supplement: Supplementary file 1 [file biomimetics-04-00067-s001.zip › Brooks_Green_Supplemental_Materials/GIFs/S5_Case3_n40mm.gif]

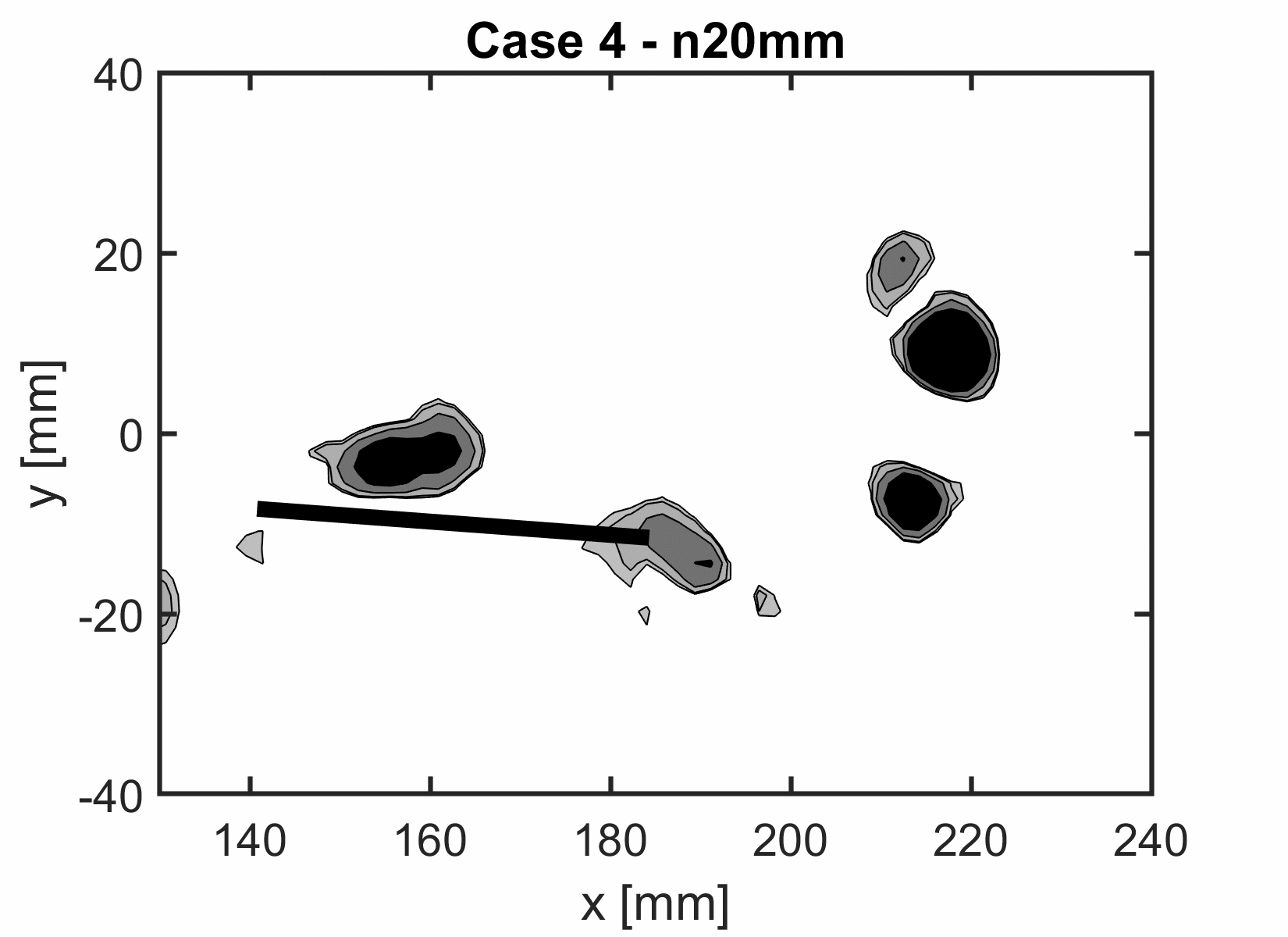

Supplement: Supplementary file 1 [file biomimetics-04-00067-s001.zip › Brooks_Green_Supplemental_Materials/GIFs/S6_Case4_n20mm.gif]

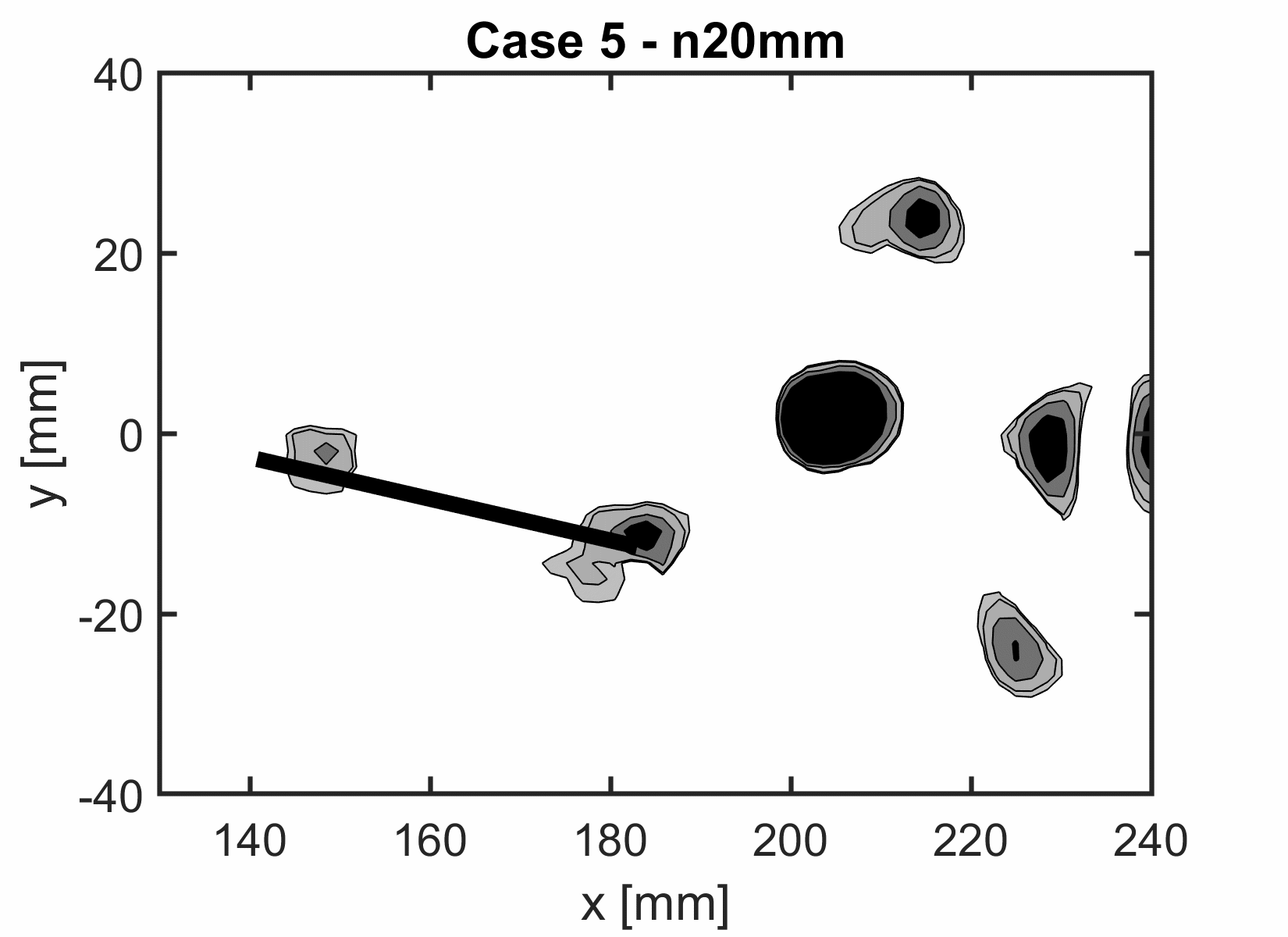

Supplement: Supplementary file 1 [file biomimetics-04-00067-s001.zip › Brooks_Green_Supplemental_Materials/GIFs/S7_Case5_n20mm.gif]

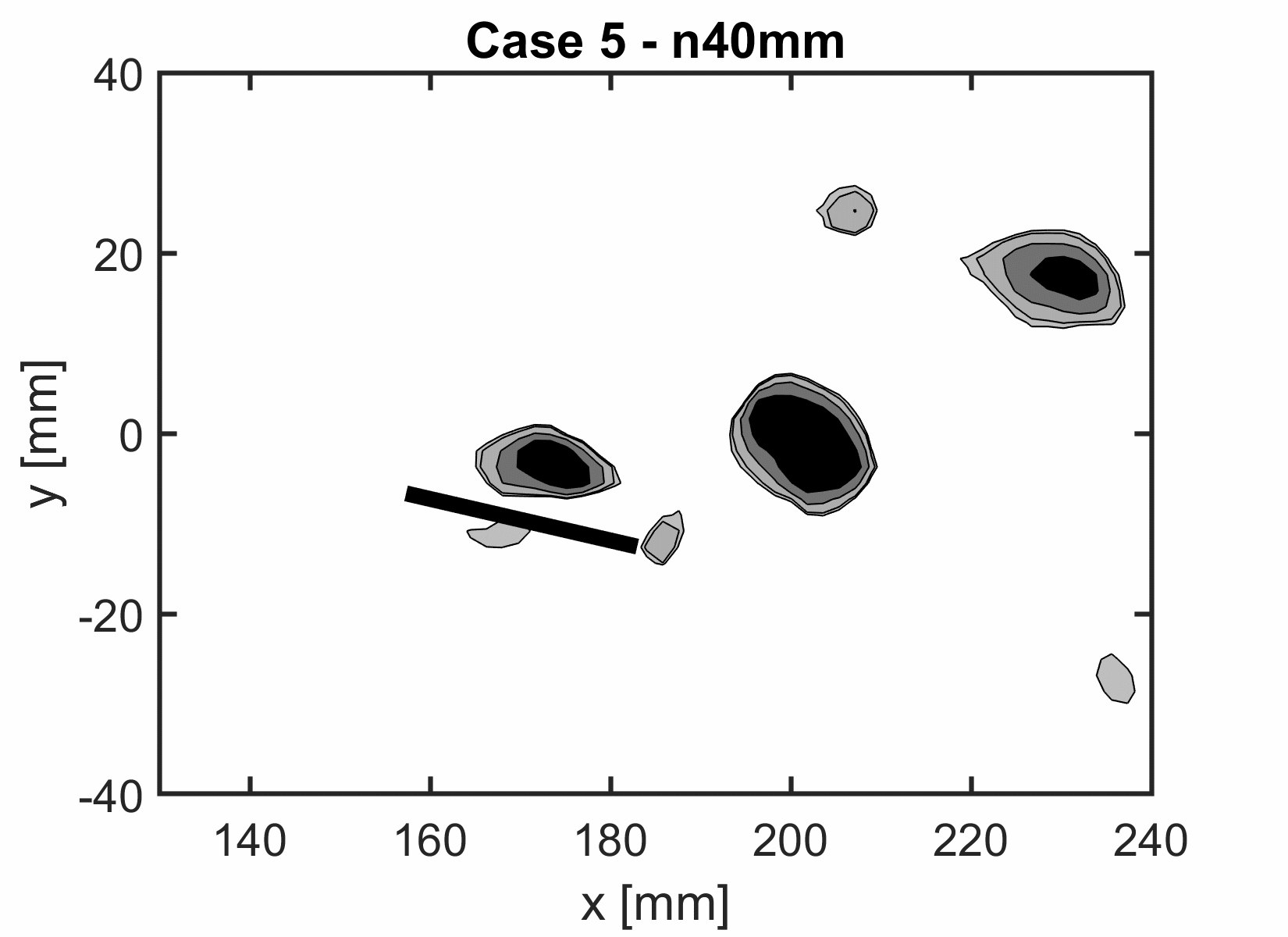

Supplement: Supplementary file 1 [file biomimetics-04-00067-s001.zip › Brooks_Green_Supplemental_Materials/GIFs/S8_Case5_n40mm.gif]

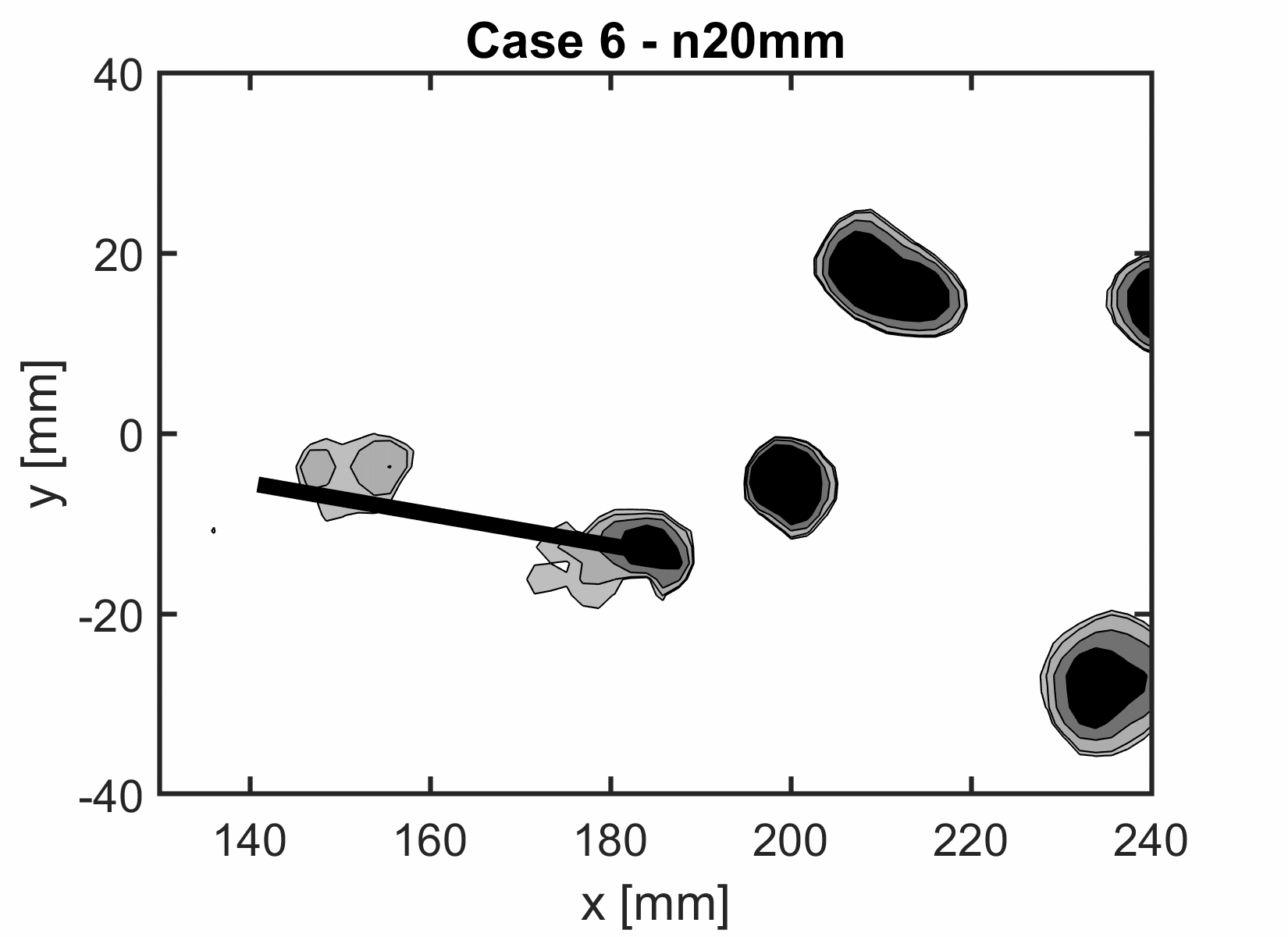

Supplement: Supplementary file 1 [file biomimetics-04-00067-s001.zip › Brooks_Green_Supplemental_Materials/GIFs/S9_Case6_n20mm.gif]
